# The chromosome-level genome assemblies of two rattans (*Calamus simplicifolius* and *Daemonorops jenkinsiana*)

**DOI:** 10.1093/gigascience/giy097

**Published:** 2018-08-07

**Authors:** Hansheng Zhao, Songbo Wang, Jiongliang Wang, Chunhai Chen, Shijie Hao, Lianfu Chen, Benhua Fei, Kai Han, Rongsheng Li, Chengcheng Shi, Huayu Sun, Sining Wang, Hao Xu, Kebin Yang, Xiurong Xu, Xuemeng Shan, Jingjing Shi, Aiqin Feng, Guangyi Fan, Xin Liu, Shancen Zhao, Chi Zhang, Qiang Gao, Zhimin Gao, Zehui Jiang

**Affiliations:** 1 State Forestry Administration Key Open Laboratory on the Science and Technology of Bamboo and Rattan, Institute of Gene Science for Bamboo and Rattan Resources, International Center for Bamboo and Rattan, Futongdong Rd, WangJing, Chaoyang District, Beijing 100102, China; 2 BGI Genomics, BGI-Shenzhen, Building No. 7, BGI Park, No. 21 Hongan 3rd Street, Yantian District, Shenzhen 518083, China; 3 BGI-Qingdao, No. 2877, Tuanjie Road, Sino-German Ecopark, Qingdao, Shandong 266555, China; 4 Research Institute of Tropical Forestry, Chinese Academy of Forestry, Guangshanyi Rd, Tianhe District, Guangzhou 510000, China; 5 State Key Laboratory of Agricultural Genomics, BGI-Shenzhen, No. 7, Pengfei Road, Dapeng District, Shenzhen 518120, China; 6 BGI-Fuyang, Floor 3, Jinshan Building, Qinghe East Road, Yingzhou District, Fuyang 236009, China

**Keywords:** rattan, Calamus simplicifolius, Daemonorops jenkinsiana, whole-genome sequencing, genome assembly, annotation

## Abstract

**Background:**

*Calamus simplicifolius* and *Daemonorops jenkinsiana* are two representative rattans, the most significant material sources for the rattan industry. However, the lack of reference genome sequences is a major obstacle for basic and applied biology on rattan.

**Findings:**

We produced two chromosome-level genome assemblies of *C. simplicifolius* and *D. jenkinsiana* using Illumina, Pacific Biosciences, and Hi-C sequencing data. A total of ∼730 Gb and ∼682 Gb of raw data covered the predicted genome lengths (∼1.98 Gb of *C. simplicifolius* and ∼1.61 Gb of *D. jenkinsiana*) to ∼372 × and ∼426 × read depths, respectively. The two *de novo* genome assemblies, ∼1.94 Gb and ∼1.58 Gb, were generated with scaffold N50s of ∼160 Mb and ∼119 Mb in *C. simplicifolius* and *D. jenkinsiana*, respectively. The *C. simplicifolius* and *D. jenkinsiana* genomes were predicted to harbor  51,235 and  53,342 intact protein-coding gene models, respectively. Benchmarking Universal Single-Copy Orthologs evaluation demonstrated that genome completeness reached 96.4% and 91.3% in the *C. simplicifolius* and *D. jenkinsiana* genomes, respectively. Genome evolution showed that four Arecaceae plants clustered together, and the divergence time between the two rattans was ∼19.3 million years ago. Additionally, we identified 193 and 172 genes involved in the lignin biosynthesis pathway in the *C. simplicifolius* and *D. jenkinsiana* genomes, respectively.

**Conclusions:**

We present the first *de novo* assemblies of two rattan genomes (*C. simplicifolius* and *D. jenkinsiana*). These data will not only provide a fundamental resource for functional genomics, particularly in promoting germplasm utilization for breeding, but also serve as reference genomes for comparative studies between and among different species.

## Background

Rattan is one of the world's most important nontimber forest products and represents a major lineage of climbing palms occurring naturally in the Old World [1]. A recent study indicates that rattan is classified into 11 genera within the tribe Calameae and subfamily Calamoideae of the family Arecaceae. Broadly, rattan consists of 631 species that occur in the same genera as climbing and nonclimbing palms [2]. Among all of these genera, *Calamus* (National Center for Biotechnology Information [NCBI] Taxon ID: 4711) and *Daemonorops* (NCBI Taxon ID: 93268) are the most diverse, accounting for ∼65% and ∼20% of rattan species [3], respectively. These two genera are also the most important material sources, providing more than 95% of the canes produced by the rattan industry. More than 5 million people depend economically on rattan, and approximately 7 billion US dollars per year are made in the rattan industry, including domestic industrial production, the international cane trade, cane splitting, plaiting materials, baskets, seats, and furniture [4]. Attention to the development of genetic breeding techniques in rattan is increasing, and the area of planted rattan is expected to gradually exceed that of natural rattans within a few years.

**Figure 1:**
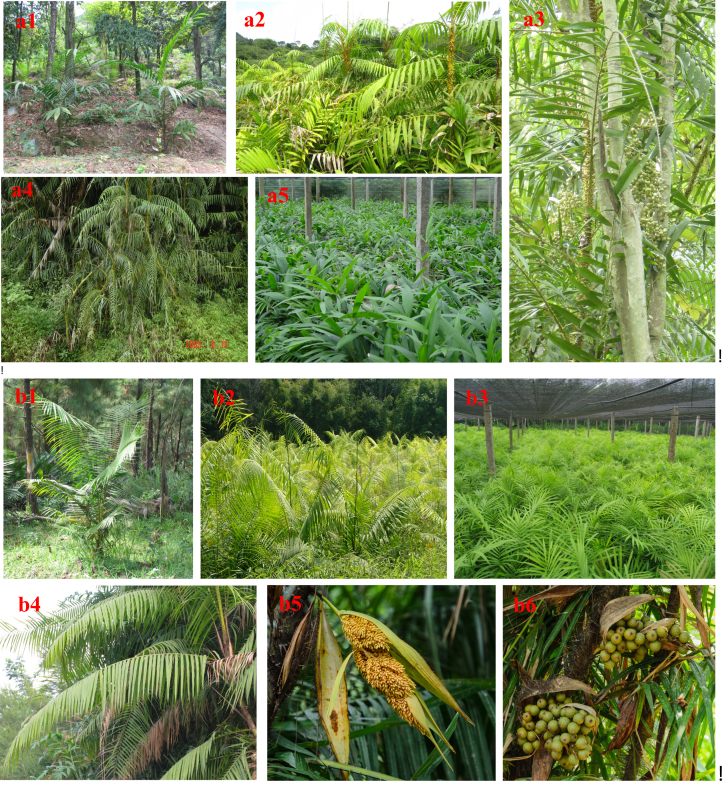
Morphological characteristics of *C. simplicifolius* and *D. jenkinsiana*. The pictures in series A and B display the different morphological characteristics of *C. simplicifolius* and *D. jenkinsiana*, respectively. **(a1)** A young *C. simplicifolius*; **(a2)** a developing *C. simplicifolius*; **(a3)** a climbing *C. simplicifolius*; **(a4)** a mature *C. simplicifolius*; **(a5)** a nursery of *C. simplicifolius*; **(b1)** a young *D. jenkinsiana*; **(b2)** a young forest of *D. jenkinsiana*; **(b3)** a nursery of *D. jenkinsiana*; **(b4)** leaves of *D. jenkinsiana*; **(b5)** inflorescences of *D. jenkinsiana*; **(b6)** young fruits of *D. jenkinsiana*. All the photos were taken by Prof Rongsheng Li.

*Calamus simplicifolius* (NCBI Taxon ID: 746888) is a deeply developed rattan species indigenous to China (Fig. 1a) that generally forms an open cluster of vigorous, unbranched stems up to 50 m long and ∼15 mm in diameter [5, 6]. An endemic rattan of Hainan Island, *C. simplicifolius* can produce high-quality canes of medium diameter for binding and weaving in the rattan industry [5]. Furthermore, *Daemonorops jenkinsiana* (NCBI Taxon ID: 1510057), a representative species of high-climbing evergreen rattan, is one of the rattan species in the *Daemonorops* genus (Fig. 1b) that naturally grows in lowland rain forests below 1,000 m above sea level, from Bangladesh, Bhutan, Cambodia, India, Laos, Myanmar, Nepal, Thailand, and Vietnam to Southeast China [2]. *Daemonorops jenkinsiana* produces a dense cluster of vigorous stems that can be up to 50 m long and ∼30 mm in diameter with internodes up to 40 cm long [6]. The two most productive rattan species, *C. simplicifolius* and *D. jenkinsiana*, are cultivated in areas with latitudes less than 23°30' N in China, i.e., Hainan Island, Guangdong, Guangxi, Yunnan, Fujian, and other areas of southern China. Their established planting areas have been estimated at more than 1,000 ha [5].

*Calamus simplicifolius* and *D. jenkinsiana* have various applications and enormous development potentials. These species are interesting mainly because of their canes, which have high pliability and remarkable durability. Molecular breeding technologies have been employed to meet the growing requirements for rattan quality and quantity. However, the lack of known genetic structure underlying the important traits of rattan has severely hampered a comprehensive understanding of its molecular biology for scientific research and actual production, as well as the in-depth performance of comparative genome analyses between and among related species. Thus, we report the two *de novo* genome assemblies of *C. simplicifolius* and *D. jenkinsiana* using the latest sequencing (Illumina and Pacific Biosciences [PacBio]) and mapping (Hi-C) technologies. With the availability of these two chromosome-level reference genomes in rattan, many comparative genome analyses and other downstream applications will become feasible, such as the development of biomarkers, the identification of functional genes, and molecular design breeding. Additionally, high-quality genome assemblies of rattan will facilitate genomic, transcriptomic, and metabolomic analyses of its material traits. As genes of possible specific interest for material improvement, members of gene families involved in lignin biosynthesis in rattan are identified here. These studies lay a foundation for future research on the utilization of these genes to improve rattan quality and diversity within rattan germplasm.

## Data Description

### DNA isolation, library construction, and sequencing

Young leaves at the vegetative growth stage were collected from *C. simplicifolius* and *D. jenkinsiana* in Spring 2015 at the Research Institute of Tropical Forestry of the Chinese Academy of Forestry in the city of Guangzhou, Guangdong Province, China (N: 23°11′29″, E: 113°22′40″, 87 m). Total DNA was isolated and extracted using DNeasy Plant Mini Kits (Qiagen) based on the manufacturer's instructions. Genomic DNA was purified according to the isolation protocol for high-molecular-weight nuclear DNA. Multiple DNA libraries were constructed [7] and sequenced on the Illumina HiSeq 4000 and PacBio Sequel platforms (Table 1). Briefly, we built three libraries with different insert sizes (270 bp, 500 bp, and 800 bp) for paired-end (PE) sequencing and four libraries with different insert sizes (2 kb, 5 kb, 10 kb, and 20 kb) for mate-pair (MP) sequencing, based on the standard Illumina protocol [8]. We also constructed five PacBio Sequel libraries with a 20-kb insert size, following the standard PacBio protocol. After data cleaning and data preprocessing, we obtained 494.08 Gb of clean data (322.3 Gb PE reads, 93.4 Gb MP reads, and 78.38 Gb PacBio data), representing 252 × coverage of the *C. simplicifolius* genome, and 426.17 Gb of clean data (244.58 Gb PE reads, 103.21 Gb MP reads, and 78.38 Gb PacBio data), representing 266 × coverage of the *D. jenkinsiana* genome.

**Table 1:** Statistics of the clean data of the *C. simplicifolius* and *D. jenkinsiana* genomes

		*C. simplicifolius*			*D. jenkinsiana*		
Sequencing platform	Insert size	Read length (bp)	Total data (Gb)	Sequence Depth (×)^a^	Read length (bp)	Total data (Gb)	Sequence depth (×)^a^
Illumina	270 bp	150	160.9	82.09	150	98.21	61.38
	500 bp	125	60.2	30.71	125	56.9	35.56
	800 bp	125	101.2	51.63	125	89.47	55.91
	2 Kb	49	22.8	11.63	49	33.08	20.67
	5 Kb	49	16.4	8.37	49	22.1	13.81
	10 Kb	49	26.8	13.67	49	32.63	20.39
	20 Kb	49	27.4	13.98	49	15.4	9.6
PacBio	20 Kb	9,079^b^	78.38	39.99	9,131^b^	78.38	48.75
Hi-C	N.A.	100	6.7	3.42	100	13.1	8.19
Total			500.78	255.5		439.27	274.26

^a^Read length for PacBio means the average length.

^b^Sequencing depth was calculated based on a 1.98 Gb *C. simplicifolius* genome and 1.61 Gb *D. jenkinsiana* genome.

As another analysis parallel to the library construction of Illumina and PacBio, two Hi-C libraries were constructed for *C. simplicifolius* and *D. jenkinsiana* using the same young leaves in BGI-Qingdao [9]. We used the *Mbo*I restriction enzyme to digest the DNA after its conformation was fixed by formaldehyde and then repaired the 5′ overhangs using biotinylated residues. Following the ligation of blunt-end fragments *in situ*, the isolated DNA was reverse-crosslinked, purified, and filtered for biotin-containing fragments. Subsequently, DNA fragment end repair, adaptor ligation, and polymerase chain reaction were performed, in that order. Then, the standard circularization step of BGISEQ-500 was carried out, and sequencing was performed using BGISEQ-500 sequencing with 100PE reads [10, 11]. Thus, we obtained ∼6.7 Gb and ∼13.1 Gb of valid data after ∼148 Gb and ∼154 Gb of raw data were evaluated and analyzed using HiC-Pro (version 2.8.0_devel) [12] in *C. simplicifolius* and *D. jenkinsiana*, respectively (Table 1).

### Genome survey

An understanding of the genomic characteristics of a given new species, i.e., genome size and heterozygosity, facilitates the development of a customized sequencing and assembly strategy. Thus, the genome size was estimated using four independent methods: a script of KmerSpectrumPlot.pl in ALLPATHS-LG (version r52488) [13], GCE (Genome Characteristics Estimation, released 7 Jan. 2015, [14]), JELLYFISH (version 2.0) [15], and flow cytometry (Supplementary Tables S1 and S2 and Figs. S1 and S2). In our genome survey, ∼98 Gb and ∼60 Gb of sequences were generated from short insert-size libraries for *C. simplicifolius* and *D. jenkinsiana*, respectively. During data preprocessing, low-quality reads (more than 40% of bases with Q<13 in a given read) were filtered out using NGS QC Toolkit (version 2.3.3) [16] with the default parameters. The combination (Supplementary Table S1) showed that the final predicted genome sizes were ∼1.98 Gb for *C. simplicifolius* and ∼1.61 Gb for *D. jenkinsiana*, and the related heterozygosity was estimated at 1.32%∼1.52% and 1.19∼1.31%, respectively. Thus, the genome survey suggested that these two rattan genomes might be suitable for a hybrid sequencing strategy using the Illumina and PacBio data.

### Hybrid *de novo* genome assembly using Illumina, PacBio, and Hi-C sequencing data

During preprocessing of the Illumina data, we filtered out low-quality reads and adaptor sequences. Thus, ∼416 Gb and ∼348 Gb of clean data were generated for *C. simplicifolius* and *D. jenkinsiana*, respectively. For the PacBio data, we used MECAT (released 27 June 2017) to correct errors [17] with the following parameters: -x 0 -i 0 -t 60 -r 0.8 -a 1000 -c 5 -l 2000. Thus, we obtained ∼52 Gb and ∼32 Gb of corrected PacBio data for *C. simplicifolius* and *D. jenkinsiana*, respectively. Subsequently, FALCON (version 0.3) [18] was chosen to perform the first assembly of the initial contigs of the two rattans. As shown in Supplementary Table S3, two assemblies using different parameters were generated for the *C. simplicifolius* genome: a 1.59 Gb assembly with a contig N50 of 67.2 kb (∼80% of the estimated genome size) and a 1.53 Gb assembly with a contig N50 of 66.7 kb (∼77% of the estimated genome size). Additionally, a 1.27 Gb assembly with a contig N50 of 81.5 kb (∼79% of estimated genome size) was obtained for *D. jenkinsiana*. The performance of MECAT for the two rattans was still not of sufficiently high quality. Thus, we considered that the incompleteness of the assembled scaffolds and low contig N50 might be due to high heterozygosity (1.32%∼1.52% for *C. simplicifolius* and 1.19∼1.31% for *D. jenkinsiana*), a high proportion of repeat sequences (54.15% for *C. simplicifolius* and 70% of *D. jenkinsiana*; see subsequent analysis for details), and inadequate sequencing depth, which was ∼26 × and ∼20 × of effective PacBio data after error correction, respectively. Therefore, taking the above findings into account, we conducted hybrid *de novo* genome assembly of *C. simplicifolius* and *D. jenkinsiana* using the Illumina and PacBio sequencing data. First, Platanus (version 1.2.4) [19], a *de novo* genome assembler for highly heterozygous data, was carried out to assemble the fragment PE reads into contigs by constructing De Bruijn graphs with an automatically optimized *k*-mer size. Second, the corrected PacBio reads and the assembled contigs were subjected to DBG2OLC (released 11 July 2015) [20] to construct scaffolds with the following parameters: DBG2OLC Contigs contig.fa LD 0 K 17 KmerCovTh 4 MinOverlap 25 AdaptiveTh 0.007 RemoveChimera 1 f scaffold.fa. Hence, we obtained ∼1.92 Gb and ∼1.56 Gb of initial assembly sequences for *C. simplicifolius* and *D. jenkinsiana*, respectively. Third, a polishing process before the SSPACE process was performed with reference to the consensus analysis of DBG2OLC (Supplementary Table S4); this step contributed to enhancing the quality of the genome assembly and reducing errors in the SSPACE process. Then, the assemblies were elongated by SSPACE (version 3.0) [21] using the MP reads, and some gaps were filled using the Illumina and PacBio data by GapCloser (version 1.12) [22] and PBJelly (released 24 Aug. 2015) [23]. Thus, we obtained an assembly of 1.96 Gb, containing 5,116 scaffolds with a contig N50 length of 107 kb and a scaffold N50 of 803 kb for *C. simplicifolius*, and we obtained an assembly of ∼1.60 Gb for *D. jenkinsiana* with N50 lengths of 108 kb and 784 kb for the contigs and scaffolds, respectively (Table 2).

**Table 2:** Metrics of the final assemblies of the *C. simplicifolius* and *D. jenkinsiana* genomes

		*C. simplicifolius*		*D. jenkinsiana*	
Items		Hybrid assembly^a^	Hi-C assembly	Hybrid assembly^a^	Hi-C assembly
Contig	Number	29,973	29,973	27,631	27,631
	Size (bp)	1,923,260,127	1,923,260,127	1,570,849,893	1,570,849,893
	N50 (bp)	99,304	99,304	89,562	89,562
	N90 (bp)	28,872	28,872	25,720	25,720
Scaffold	Number	29,775	5,283	27,146	5,126
	Size (bp)	1,923,287,712	1,935,533,712	1,570,878,714	1,581,888,714
	N50 (bp)	99,590	160,072,219	89,705	119,093,744
	N90 (bp)	28,922	93,668,489	25,828	61,330,142
Total number	>3 kb	29,767	5,275	27,137	5,117
	>5 kb	29,727	5,235	27,081	5,061
Longest sequence (bp)		877,470	219,145,773	1,422,351	162,635,149
Shortest sequence (bp)		1,286	1,286	719	719
Ratio of ambiguous bases (%)		0.0	0.6	0.0	0.7
GC ratio (%)		41.07	41.07	41.78	41.78

^a^Hybrid assembly means *de novo* assembly using Illumina and PacBio data in our study.

**Figure 2:**
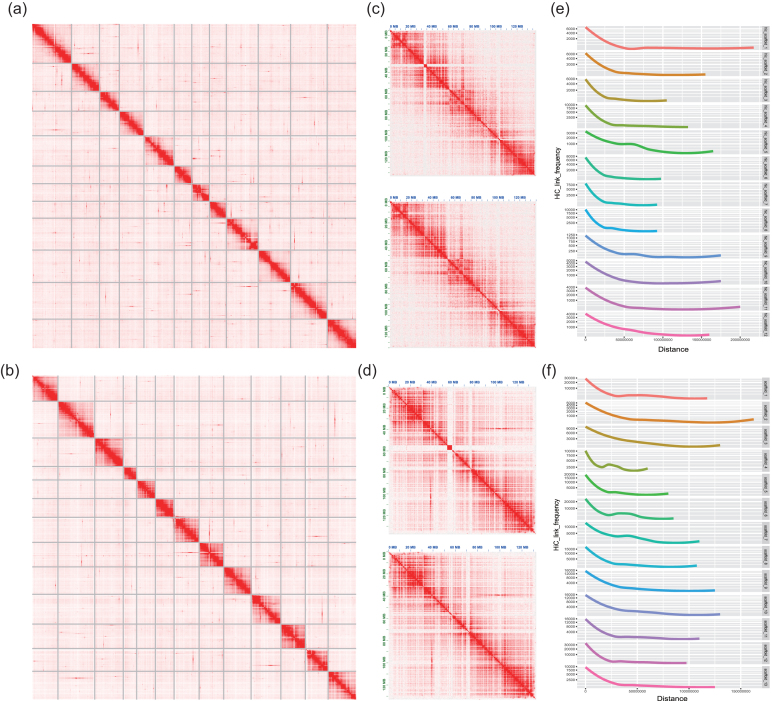
Hi-C contact map of the *C. simplicifolius***(a)** and *D. jenkinsiana* genomes **(b).(c) and (d)** The Hi-C links on hic_scaffold_4 of *C. simplicifolius* and hic_scaffold_10 of *D. jenkinsiana* before (top) and after (bottom) conflict resolution. **(e) and (f)** The distribution of Hi-C link decay along the genomic distance.

Subsequently, the valid Hi-C data together with the above assembly were processed by the 3D-DNA pipeline (version 170123) [24] to produce chromosome-level scaffolds. We obtained an explicit contact pattern, which implied a reasonably accurate chromosome-level assembly. As shown in Fig. 2, the contact maps were visualized by Juicebox (version 1.5.2) [25]. The lengths of the longest 12 chromosome-level scaffolds for the *C. simplicifolius* assembly and the 13 for the *D. jenkinsiana* assembly are presented in Supplementary Table S5. The total lengths of the pseudochromosomes accounted for 92.08% and 92.01% of the *C. simplicifolius* and *D. jenkinsiana* genomes, with scaffold N50 values of 169 Mb and 119 Mb, respectively.

### Genome evaluation

Three independent methods were used to evaluate the accuracy and completeness of the *C. simplicifolius* and *D. jenkinsiana* assemblies. First, two genome features were summarized: the percentage of ambiguous bases (Ns) and Guanine and Cytosine (GC) content. The results showed a low percentage of Ns (∼0.6% for *C. simplicifolius* and ∼0.7% for *D. jenkinsiana*) in each genome, and the overall GC contents (41.07% for *C. simplicifolius* and 41.78% for *D. jenkinsiana*) were similar to those of the related transcriptomic data (41.68% for *C. simplicifolius* and 41.89% for *D. jenkinsiana*). Then, the unigenes assembled from the RNA sequencing (RNA-seq) data were aligned to the assembly using the Basic Local Alignment Search Tool (BLAST)-like alignment tool (version 1.0) [26] with the default parameters. The alignment results showed that more than 90% of the sequences in one scaffold could be aligned with assembly (92.89% of *C. simplicifolius* and 81.81% of *D. jenkinsiana*) (Supplementary Table S6). Last, the completeness of the two rattan assemblies was evaluated using BUSCO (version 3.0) [27], which quantitatively assesses genome completeness using evolutionarily informed expectations of gene content from near-universal single-copy orthologs. The BUSCO results showed that 96.4% of conserved BUSCO proteins (embryophyta_odb9) were detected in the *C. simplicifolius* assembly, including 3.8% of fragment BUSCO proteins. Additionally, 87.3% and 4.0% of the conserved BUSCO proteins were identified as complete and fragment proteins in *D. jenkinsiana*, respectively (Supplementary Table S7).

### Repeat annotation

**Figure 3:**
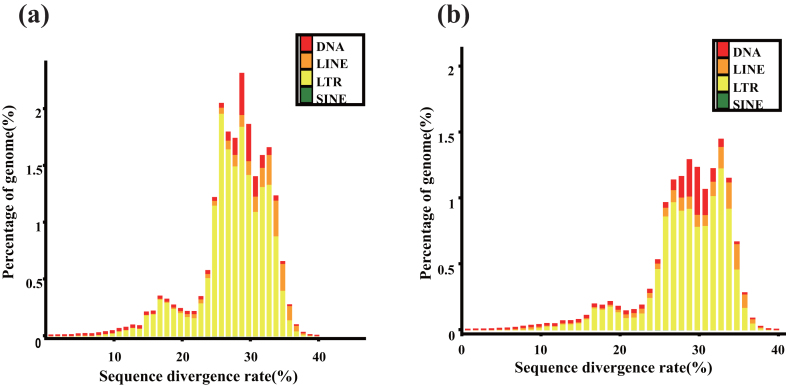
Distribution of the sequence divergence rates of different TE types in the *C. simplicifolius***(a)** and *D. jenkinsiana***(b)** genomes.

Before protein-coding gene model prediction, transposable elements (TEs) and tandem repeats were identified in the *C. simplicifolius* and *D. jenkinsiana* assemblies. We adopted two independent approaches to predict repetitive elements: homology-based annotation and *de novo* methods. In the homology-based annotation, TEs were identified using RepeatMasker (v4.0.5) and RepeatProteinMasker (v4.0.5) [28] via searching against the Repbase library (released 01 Dec. 2017) [29]. In the *de novo* annotation, a *de novo* repeat library was constructed using RepeatModeler (v1.0.8) [30] and LTR_FINDER [31] after eliminating contaminants and multicopy genes. Then, RepeatMasker was used to categorize the genome sequences against the *de novo* repeat library. Additionally, tandem repeat sequences were identified by Tandem Repeat Finder (version 4.09) [32] with the following parameters: “Match = 2, Mismatch = 7, Delta = 7, PM = 80, PI = 10, Minscore = 50 and MaxPeriod = 2000.” Overall, the results showed that long terminal repeat (LTR) was the most abundant repeat type and that short interspersed nuclear element and long interspersed nuclear element, two non-LTR retrotransposons, had the lowest proportions in the two rattan assemblies (Supplementary Table S8). TEs accounted for 54.15% and 70% of the *C. simplicifolius* and of *D. jenkinsiana* assemblies, respectively, and the sequence divergence of TEs indicated that the *de novo*-predicted repeats were more recently active than the Repbase-predicted repeats (Fig. 3).

### RNA sample collection, library construction, and transcriptome assembly

Four samples of the distal cirrus at three developmental stages were collected from *C. simplicifolius* and *D. jenkinsiana*. Each sample had three biological replicates (Supplementary Table S9). Because this experiment was a part of the rattan genome project, the location of RNA sampling was consistent with that of DNA sampling. Based on the manufacturer's instructions, RNA was isolated using TRIzol Reagent Solution (Invitrogen, Carlsbad, CA, USA), and the purity and concentration were determined with a NanoDrop 2000 spectrophotometer. Reverse transcription was conducted with a Reverse Transcription System (Promega, USA). The extracted RNA was treated with RNase-free DNase I for 30 minutes at 37°C to remove residual DNA, as described previously [33], and then, the pooled libraries were sequenced using the BGISEQ-500 platform with short 100PE reads. When preprocessing the transcriptomic data, adaptor sequences and low-quality reads were filtered using SOAPnuke (version 1.5.6) [34] with the following parameters: “-n 0.001 -l 20 -q 0.4 -Q 2.” The clean reads of all samples were assembled using Trinity (version 2.0.6) [35] with the following parameters: (1) group_pairs_distance 500, (2) min_contig_length 200, (3) min_kmer_cov 2, (4) min_glue 2, (5) bfly_opts -V 5, (6) edge-thr = 0.1, (7) stderr, and (8) SS_lib_type RF. Then, the outputs of Trinity were clustered to generate a single set of nonredundant references using TGI Clustering Tool (version v2.0.6) [36] with the following parameters: (1) a minimum of 95% identity between the contigs, (2) a minimum of 35 overlapping bases, (3) a minimum score of 35, and (4) a maximum of 20 unmatched overhanging bases at the sequence ends. Ultimately, the assembled transcripts were divided into two classes based on sequence similarity: clusters (prefixed with “CL”) and singletons (prefixed with “unigene”). In each cluster, the sequence similarity regions between the transcripts were more than 70%, and the transcripts were spliced isoforms from a gene or a paralogous gene. Additionally, all unigenes were used in subsequent analyses.

### Gene modeling and prediction

We performed an integrated prediction of intact protein-coding gene models using three independent approaches [7], i.e., *de novo* prediction, homology-based method, and RNA-seq approach. The repeat masked assembly was first annotated by AUGUSTUS (version 3.3) with the default parameters [37]; this program is a *de novo* predictor based on a self-trained model. After the training data were optimized and multiple trainings were performed,  85,246 and  87,613 gene models were predicted for *C. simplicifolius* and *D. jenkinsiana*, respectively. In the homology-based prediction, we used the following seven species as reference datasets: *Elaeis guineensis, Phoenix dactylifera, Brachypodium distachyon, Oryza sativa, Setaria italica, Sorghum bicolor*, and *Zea mays* (for individual genome versions, see Availability of Supporting Data). Their protein sequences were downloaded from the ENSEMBL database [38] and aligned to the *C. simplicifolius* and *D. jenkinsiana* assemblies using TBLASTN (version 2.2.26) [39] with an E-value cutoff of 1e-5. Then, splicing patterns were generated by GeneWise (version 2.0) [40]. In the RNA-seq analysis, HISAT2 (version 2.0.2) [41] was used to identify exon-intron splicing junctions and refine the alignment of the RNA-seq reads to the genome. We then used Cufflinks (version 2.2.1) [42] to define  56,024 and  58,134 protein-coding gene models in *C. simplicifolius* and *D. jenkinsiana*, respectively (Supplementary Table S10).

Last, we integrated the evidence from the three above independent predictions using MAKER (version 2) [43]. The final prediction results showed that  51,235 and  53,342 intact protein-coding gene models were predicted as consensus gene sets in *C. simplicifolius* and *D. jenkinsiana*, respectively.

### Annotation evaluation and gene function prediction

We evaluated the predicted annotations using two independent methods: gene function evaluation and completeness evaluation by BUSCO. In the gene function evaluation, we assessed the agreement of the predicted annotations with protein alignment searches for homologous proteins in closely related species and manual annotations. The results of alignments against five authoritative protein databases (Supplementary Table S11) indicated that 5.34% and 2.89% of the predicted gene models were identified as unannotated genes in *C. simplicifolius* and *D. jenkinsiana*, respectively. These protein databases included the NCBI nonredundant protein database (released 13 March 2018) [44], SWISS-PROT (released 1 Jan. 2018) [45], Gene Ontology (GO) (released 30 Oct. 2013) [46], Kyoto Encyclopedia of Genes and Genomes (KEGG) (dataset v81) [47], and InterPro (dataset v.53) [48]. Additionally, the BUSCO evaluation showed that 88.7% and 91.3% of conserved BUSCO proteins (embryophyta_odb9) were present in *C. simplicifolius* and *D. jenkinsiana*, respectively. Among the conserved BUSCO proteins, 76.2% and 81.2% were complete. Furthermore, the four types of noncoding RNA genes, i.e., tRNA, rRNA, miRNA, and snRNA, were also predicted (Supplementary Table S12).

### Gene family construction and rattan-specific gene families

**Figure 4:**
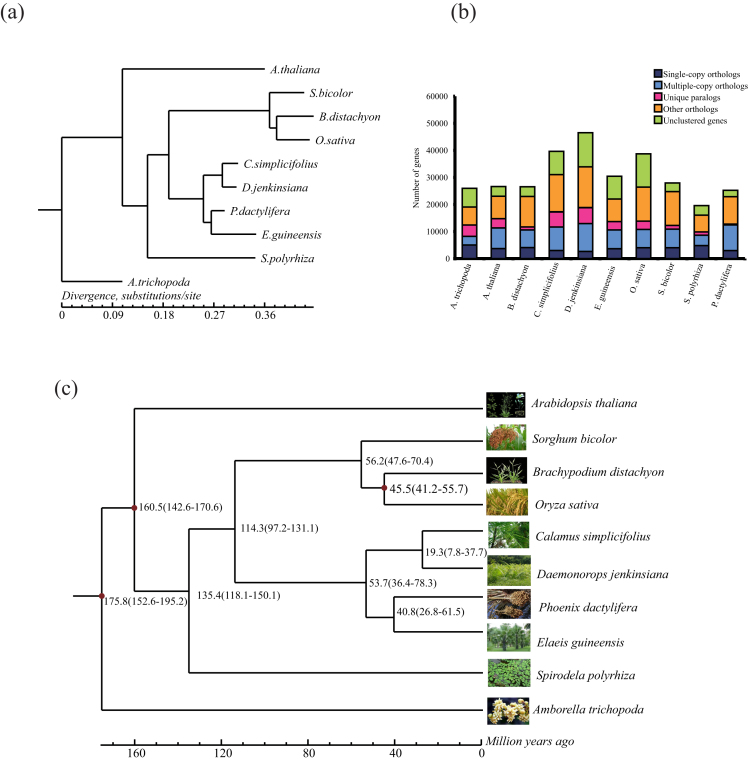
The phylogenetic tree, orthologous gene families, and divergence times among *C. simplicifolius, D. jenkinsiana*, and eight other plants. **(a)** The phylogenetic tree was constructed by RAxML using all single-copy genes in the 10 species, and the divergence times were estimated using the MCMCTree program in the PAML software package. **(b)** Clusters of orthologous and paralogous gene families in *C. simplicifolius, D. jenkinsiana*, and other eight fully sequenced plants using OrthoMCL. **(c)** The numbers on the nodes are divergence times, and the red nodes indicate the calibration times.

In our study, we performed a pairwise sequence comparison to predict orthologous genes at the genome level. This method is rapid and generally deals well with large amounts of data. A popular BLAST-based approach, OrthoMCL (version 2.0.9) [49], was used to identify orthologous genes in *C. simplicifolius* and *D. jenkinsiana* with an E-value cutoff of 1e-5 and a percent match cutoff of 80 (i.e., query and match were required to overlap on more than 80% of the query and match sequence length). Markov chain clustering was also used with a default inflation parameter in an all-to-all BLASTP analysis of entries for the other eight plants, i.e., *Amborella trichopoda, E. guineensis, A. thaliana, B. distachyon, O. sativa, Spirodela polyrhiza, P. dactylifera*, and *S. bicolor* (for individual genome versions, see Availability of Supporting Data). Among the  30,936 gene families identified in all 10 species,  44,700 and  44,537 orthologous genes were detected in the *C. simplicifolius* and *D. jenkinsiana* genomes, respectively. Approximately 6,132 (19.8%) gene families common to all 10 species as well as 2,366 and 2,707 specific gene families were detected in *C. simplicifolius* and *D. jenkinsiana*, respectively (Fig. 4b). Additionally, the results showed that 637 gene families were specific to the rattans. These rattan-specific gene families were enriched in gene ontology categories related to component membrane and transcription factor activity (Supplementary Table S13) and in KEGG pathways related to plant-pathogen interaction and plant hormone signal transduction (Supplementary Table S14).

### Phylogenetic analysis and divergence time

We obtained 962 single-copy orthologous genes derived from entire gene families that were conserved among the species to facilitate an understanding of the evolutionary relationships of rattans with other species. First, multiple alignments of protein sequences were conducted by MUSCLE (version 3.8.31) [50]; then, a coding DNA sequence (CDS) alignment was constructed based on the protein alignments. Subsequently, all aligned CDSs were concatenated to generate a supergene for each species using an in-house Perl script. Thus, we extracted the nucleotides at position 2 (phase 1) of each codon to construct the phylogenetic tree using RAxML (version 8.2.3) [51] with the model “GTRGAMMA.” The results showed that four species of Arecaceae were located in a cluster, which comprised two independent sister branches with one containing *C. simplicifolius* and *D. jenkinsiana* and the other containing *E. guineensis* and *P. dactylifera* (Fig. 4a).

Moreover, we used the MCMCTree program of PAML (version 4.5) [52] to estimate the divergence times among *C. simplicifolius, D. jenkinsiana*, and the other eight species with the following parameters: “-nsample 200000 -burnin 40000.” The calibration times were derived from published times for the divergences of the reference species [53]. The results indicated that the divergence time between the two rattans was ∼19.3 million years ago (Mya), and for the other two Arecaceae species, *P. dactylifera* separated from *E. guineensis* at ∼40.8 Mya (Fig. 4c).

### Genome-wide identification of gene families involved in the lignin biosynthesis pathway

Lignins are a class of complex aromatic heteropolymers of monolignols that encrust and interact with the cellulose/hemicellulose matrix of the secondary cell wall. The aromatic lignin polymers are commonly composed of three monolignols, i.e., *p*-hydroxyphenyl (H), vanillin (G), and syringaldehyde (S) [54]. Thus, we performed genome-wide identification of 13 gene families involved in the lignin biosynthesis pathway of rattan using eight genomes, i.e., *A. thaliana, B. distachyon, O. sativa, S. bicolor, Phyllostachys edulis, Populus trichocarpa, D. jenkinsiana*, and *C. simplicifolius*. Most genome sequences (*A. thaliana, B. distachyon, O. sativa, S. bicolor*, and *Po. trichocarpa*) were downloaded from the ENSEMBL database [55]. The genome sequence of *Ph. edulis* was downloaded from the Bamboo Genome Database [56]. Based on wide literature-based investigations, 140 genes involved in the lignin biosynthetic pathway were collected based on experimental validation in previous studies (Supplementary Table S15); then, these known genes were used as query sequences for further gene identification. A BLAST search and domain analysis, as described previously [55], were used in the genome-wide gene identification process. Briefly, we performed standard protein BLAST searches (version 2.2.26) against all genome sequences including those of the two rattans using the coding sequences of known genes with the following cutoff values: E-value <1e-10; identity >40%; and coverage rate >95% of query sequence. The filtered sequences were subsequently analyzed by hmmsearch (version 3.1b2) using the Pfam-A.hmm database (released 31 March 2017), and unclear sequences with incomplete domains were discarded by manual correction. The results showed that the expansion of most lignin-related gene families was detected in the two rattans (Table 3). Each gene family contained multiple members, with an average of ∼15 and ∼13 gene members per family in *C. simplicifolius* and *D. jenkinsiana*, respectively. The total numbers of genes in the lignin biosynthesis pathway were 193 and 172 genes in *C. simplicifolius* and *D. jenkinsiana*, respectively. Peroxidase, as the most common gene, was detected in both rattans. Among the least common genes, phenylalanine ammonia-lyase was identified in *C. simplicifolius*, and coumarate 3-hydroxylase and cinnamate 4-hydroxylase were detected in *D. jenkinsiana*. The observed expansion of lignin biosynthesis genes in rattan could be due to the occurrence of a whole-genome duplication (WGD) event, since a WGD could provide more gene copies, which facilitates the evolution of genes with new functions [57].

**Table 3:** Numbers of genes in gene families of the lignin biosynthesis pathway

Family	*C. simplicifolius*	*D. jenkinsiana*	*A. thaliana*	*B. distachyon*	*O. sativa*	*Ph. edulis*	*Po. trichocarpa*	*S. bicolor*	Total
4-coumarate CoA ligase	9	13	12	13	12	15	13	16	90
Coumarate 3-hydroxylase	3	2	3	1	1	3	3	2	15
Cinnamate 4-hydroxylase	3	2	1	2	3	6	2	2	19
Cinnamyl alcohol dehydrogenase	29	22	9	7	10	14	17	11	102
Caffeoyl-CoA 3-O-methyltransferase	16	5	4	7	6	9	5	5	52
Cinnamoyl-CoA reductase	6	6	3	9	12	17	10	11	64
Caffeic acid 3-O-methyltransferase	13	16	11	4	6	4	11	5	59
Ferulate 5-hydroxylase	7	6	1	4	5	16	17	11	50
Hydroxycinnamoyl-CoA	5	4	3	12	6	16	7	13	59
Laccase	29	29	16	22	20	41	47	21	178
Phenylalanine ammonia-lyase	2	7	4	9	8	12	5	10	52
Chalcone synthase	31	17	4	7	17	12	13	27	115
Peroxidase	40	43	45	44	37	77	56	42	328
Total	193	172	116	141	143	242	206	176	–

## Conclusion

Here, we report two chromosome-level reference genome sequences of rattan (*C. simplicifolius* and *D. jenkinsiana*) using multiple types of sequencing data and assembly technologies. These *C. simplicifolius* [58] and *D. jenkinsiana* [59] genomes should facilitate the *de novo* genome assembly and resequencing of other rattan species and serve as essential resources to identify regions that provide suitable resolution in the evolutionary landscape by performing comparative studies between and among different species. The availability of two high-quality rattan genomes simplifies the identification of critical genes involved in the lignin biosynthesis pathway, which have potential importance for rattan growth and development. Therefore, these data pave the way for additional genomic studies in rattan and related species.

## Availability of supporting data

The datasets and materials supporting the results presented in this article are available in the *Giga*DB repository [58-60]. All raw genomic sequence reads from the BGISEQ-500, Illumina, and PacBio platforms and the transcriptome reads derived from multiple tissues have been uploaded and deposited in the European Nucleotide Sequence Archive (EMBL-EBI) with the project accession numbers PRJEB24031 and PRJEB24829 for *C. simplicifolius* and *D. jenkinsiana*, respectively. Other data analyzed in this study included *A. trichopoda* (version 1.0), downloaded from the Amborella Genome Database (amborella.huck.psu.edu), and *E. guineensis* (version GCF_000 442705.1), downloaded from NCBI. The remaining genomes were downloaded from the ENSEMBL database, including *E. guineensis* (version GCF_000 442705.1), *Ph. dactylifera* (version 1.0), *B. distachyon* (version 3.1), *O. sativa* (version R498), *S. italica* (version 9.0), *S. bicolor* (version 3.1), *Z. mays* (version B73_RefGen_V4), *Ph. edulis* (version 2), *Po. trichocarpa* (JGI2.0.31), and *A. thaliana* (version: TAIR10).

## Additional files

**Additional Table S1:** Evaluation of genome sizes of *C. simplicifolius* and *D. jenkinsiana*

**Additional Table S2:** The 17-mer frequency method estimated genome sizes of *C. simplicifolius* and *D. jenkinsiana*

**Additional Table S3:** Statistics of the assemblies using different assembly strategies

**Additional Table S4:** BUSCO evaluation of the polishing process

**Additional Table S5:** The chromosome-level lengths of the Hi-C assemblies for *C. simplicifolius* and *D. jenkinsiana*

**Additional Table S6:** Statistics of the quality assessment of the *C. simplicifolius* and *D. jenkinsiana* genomes

**Additional Table S7:** BUSCO evaluation of the *C. simplicifolius* and *D. jenkinsiana* genomes

**Additional Table S8:** Statistics of the predicted repetitive sequences in the *C. simplicifolius* and *D. jenkinsiana* genomes

**Additional Table S9:** Statistics of RNA libraries in the transcriptome assemblies

**Additional Table S10:** Statistics of the predicted protein-coding genes in the *C. simplicifolius* and *D. jenkinsiana* genomes

**Additional Table S11:** Statistics of functional annotations of the *C. simplicifolius* and *D. jenkinsiana* genomes

**Additional Table S12:** Statistics of the predicted noncoding RNAs in the *C. simplicifolius* and *D. jenkinsiana* genomes

**Additional Table S13:** GO analysis of rattan-specific gene families

**Additional Table S14:** KEGG analysis of rattan-specific gene families

**Additional Table S15:** A total of 140 genes in the lignin biosynthetic pathway experimentally validated in previous studies

**Additional Figure S1:** Evaluation of the genome sizes of *C. simplicifolius* and *D. jenkinsiana* by K-mer

**Additional Figure S2:** Evaluation of the genome size of *C. simplicifolius* and *D. jenkinsiana* by flow cytometry

## Abbreviations

BLAST: Basic Local Alignment Search Tool; BUSCO: Benchmarking Universal Single-Copy Ortholog; CDS: coding DNA sequence; GC:Guanine and Cytosine; GO: Gene Ontology; KEGG: Kyoto Encyclopedia of Genes and Genomes; LINE: long interspersed nuclear element; LTR: long terminal repeat; MP: mate pair; Mya: million years ago; NCBI: National Center for Biotechnology Information; Ns: ambiguous base; PacBio: Pacific Biosciences; PE: paired end; RNA-Seq: RNA-sequencing; SINE: short interspersed nuclear element; TE: transposable element; WGD: whole-genome duplication.

## Competing interests

The authors have declared that they have no competing interests.

## Funding

This work was supported by the Sub-Project of the National Science and Technology Support Plan of the Twelfth Five-Year Plan in China (2015BAD04B03 and 2015BAD04B01), Fundamental Research Funds for the International Center for Bamboo and Rattan (1632017018), and the Science Technology and Innovation Committee of Shenzhen Municipality (JCYJ20160331190123578).

## Author contributions

H.S.Z. and R.S.L. collected the samples; J.L.W., H.Y.S., S.N.W., H.X., K.B.Y., X.R.X., X.M.S., and J.J.S. constructed libraries; and H.S.Z., S.B.W., C.H.C., L.F.C., A.Q.F., C.Z., and Q.G. performed the genome assembly. S.J.H., K.H., C.C.S., and G.Y.F. performed the Hi-C analysis. S.B.W. and L.F.C. performed the genome annotation and H.S.Z., S.B.W., L.F.C., and X.L. analyzed the genome data. H.S.Z. and S.B.W. wrote the manuscript and H.S.Z., S.B.W., X.L., Z.M.G., and Z.H.J. reviewed the manuscript. All of the above authors read and approved the final manuscript.

## Acknowledgements

As a part of the Genome Atlas of Bamboo and Rattan (GABR), we acknowledge the GABR Consortium members, partners, advisors, and supporters who have helped to make this project run smoothly.

## Supplementary Material

GIGA-D-18-00152_Original_Submission.pdf

GIGA-D-18-00152_Revision_1.pdf

GIGA-D-18-00152_Revision_2.pdf

Response_to_Reviewer_Comments_Original_Submission.pdf

Response_to_Reviewer_Comments_Revision1.pdf

Reviewer_1_Report_(Original_Submission) -- Yinping Jiao 5/17/2018 Reviewed

Reviewer_1_Report_(Revision_1) -- Yinping Jiao 06/22/2018 Reviewed

Reviewer_2_Report_(Original_Submission) -- Denis Murphy 05/29/2018 Reviewed

Reviewer_2_Report_(Revision_1) -- Denis Murphy 6/9/2018 Reviewed

Supplemental Files
