# Supplementary material for: The chromosome-level genome assemblies of two rattans (Calamus simplicifolius and Daemonorops jenkinsiana)
Source: Gigascience. 2018 Aug 7;7(9):giy097. doi: 10.1093/gigascience/giy097 (PMC6117794; doi:10.1093/gigascience/giy097)
Supplement: GIGA-D-18-00152_Original_Submission.pdf [file giy097_giga-d-18-00152_original_submission.pdf]

GigaScience

The chromosome-level genome assemblies of two rattans (Calamus simplicifolius and Daemonorops jenkinsiana)

--Manuscript Draft--

Manuscript Number:	GIGA-D-18-00152
Full Title:	The chromosome-level genome assemblies of two rattans (Calamus simplicifolius and Daemonorops jenkinsiana)
Article Type:	Data Note
Funding Information:	the Sub-Project of the National Science and Technology Support Plan of the Twelfth Five-Year Plan in China (2015BAD04B03) — Prof. Hansheng Zhao
Abstract:	Background: Calamus simplicifolius and Daemonorops jenkinsiana are two representative rattans, as the most significant material sources for the rattan industry. However, the lack of a reference genome sequence is a major obstacle for basic and applied biology on rattan.<br><br>Findings: We rendered the two chromosome-level genome assemblies of C. simplicifolius and D. jenkinsiana using the Illumina, PacBio, and Hi-C sequencing data. A total of ~730 Gb and ~682 Gb of raw data covered the predicted genome length (~1.98 Gb of C. simplicifolius and ~1.61 Gb of D. jenkinsiana) to ~ 372× and ~ 426× read depth, respectively. The two de novo genome assemblies of ~1.94 Gb and ~1.58 Gb are generated with the scaffold N50 of ~160 Mb and ~119 Mb in C. simplicifolius and D. jenkinsiana, respectively. The C. simplicifolius and D. jenkinsiana genome were predicted to harbor 51,235 and 53,342 intact protein-coding gene models, respectively. BUSCO evaluation demonstrated that the genome completeness reached 96.4% and 91.3% in the C. simplicifolius and D. jenkinsiana genome, respectively.Additionally, genome evolution revealed that the ancestor of rattans was separated from Elaeis guineensis at ~113 Mya.<br><br>Conclusions: To our knowledge, we present the first de novo assemblies of the two rattan genomes (C. simplicifolius and D. jenkinsiana) and performed a couple of basic analyses, including hybrid de novo assembly, genome annotation, and phylogenetic study. These essential data will not only provide a fundamental resource of functional genomics particularly in promoting germplasm utilization for breeding improved rattan varieties, but also serve as two reference genomes for performing comparative studies between and among different species.
Corresponding Author:	Hansheng Zhao<br>International Center for Bamboo and Rattan<br>Beijing, Beijing CHINA
Corresponding Author Secondary Information:	
Corresponding Author's Institution:	International Center for Bamboo and Rattan
Corresponding Author's Secondary Institution:	
First Author:	Hansheng Zhao
First Author Secondary Information:	
Order of Authors:	Hansheng Zhao
	Songbo Wang
	Jiongliang Wang
	Chuanhai Chen
	Shijie Hao

Powered by Editorial Manager® and ProduXion Manager® from Aries Systems Corporation

	Lianfu Chen
	Benhua Fei
	Kai Han
	Rongsheng Li
	Chengcheng Shi
	Huayu Sun
	Sining Wang
	Hao Xu
	Kebin Yang
	Xiurong Xu
	Xuemeng Shan
	Jingjing Shi
	Aiqin Feng
	Guangyi Fan
	Xin Liu
	Shancen Zhao
	Chi Zhang
	Qiang Gao
	Zhimin Gao
	Zehui Jiang
**Order of Authors Secondary Information:**	
**Additional Information:**	
**Question**	**Response**
Are you submitting this manuscript to a special series or article collection?	No
**Experimental design and statistics**<br><br>Full details of the experimental design and statistical methods used should be given in the Methods section, as detailed in our Minimum Standards Reporting Checklist. Information essential to interpreting the data presented should be made available in the figure legends.<br><br>Have you included all the information requested in your manuscript?	Yes
**Resources**<br><br>A description of all resources used, including antibodies, cell lines, animals and software tools, with enough information to allow them to be uniquely	Yes

Powered by Editorial Manager® and ProduXion Manager® from Aries Systems Corporation

identified, should be included in the Methods section. Authors are strongly encouraged to cite Research Resource Identifiers (RRIDs) for antibodies, model organisms and tools, where possible.<br><br>Have you included the information requested as detailed in our Minimum Standards Reporting Checklist?	
**Availability of data and materials**<br><br>All datasets and code on which the conclusions of the paper rely must be either included in your submission or deposited in publicly available repositories (where available and ethically appropriate), referencing such data using a unique identifier in the references and in the “Availability of Data and Materials” section of your manuscript.<br><br>Have you have met the above requirement as detailed in our Minimum Standards Reporting Checklist?	Yes

Powered by Editorial Manager® and ProduXion Manager® from Aries Systems Corporation

Manuscript
Click here to download Manuscript Manuscript_TwoRattans0508.docx

The chromosome-level genome assemblies of two rattans (*Calamus simplicifolius* and *Daemonorops jenkinsiana*)

Hansheng Zhao[1#], Songbo Wang[2#], Jiongliang Wang[1#], Chuanhai Chen[2#], Shijie Hao[3], Lianfu Chen[1], Benhua Fei[1], Kai Han[3], Rongsheng Li[4], Chengcheng Shi[3], Huayu Sun[1], Sining Wang[1], Hao Xu[1], Kebin Yang[1], Xiurong Xu[1], Xuemeng Shan[1], Jingjing Shi[1], Aiqin Feng[2], Guangyi Fan[3], Xin Liu[3], Shancen Zhao[2], Chi Zhang[2], Qiang Gao[2*], Zhimin Gao[1*], and Zehui Jiang[1*]

[1] State Forestry Administration Key Open Laboratory on the Science and Technology of Bamboo and Rattan, Institute of Gene Science for Bamboo and Rattan Resources, International Center for Bamboo and Rattan, Futongdong Rd, WangJing, Chaoyang District, Beijing 100102, China;

[2] BGI Genomics, BGI-Shenzhen, Building NO.7, BGI Park, No. 21 Hongan 3rd Street, Yantian District, Shenzhen 518083, China;

[3] BGI-Qingdao, No. 2877, Tuanjie Road, Sino-German Ecopark, Qingdao, Shandong 266555, China;

[4] Research Institute of Tropical Forestry, Chinese Academy of Forestry, Guangshanyi Rd, Tianhe District, Guangzhou 510000, China.

[#] These authors contributed equally to this work.

[*] To whom correspondence should be addressed: Qiang Gao (gaoqiang@bgi.com), Zhimin Gao (gaozhimin@icbr.ac.cn), and Zehui Jiang (jiangzehui@icbr.ac.cn)

Manuscript type: Data note

Abstract

Background: *Calamus simplicifolius* and *Daemonorops jenkinsiana* are two representative rattans, as the most significant material sources for the rattan industry. However, the lack of a reference genome sequence is a major obstacle for basic and applied biology on rattan.

Findings: We rendered the two chromosome-level genome assemblies of *C. simplicifolius* and *D. jenkinsiana* using the Illumina, PacBio, and Hi-C sequencing data. A total of ~730 Gb and ~682 Gb of raw data covered the predicted genome length (~1.98 Gb of *C. simplicifolius* and ~1.61 Gb of *D. jenkinsiana*) to ~ 372× and ~ 426× read depth, respectively. The two *de novo* genome assemblies of ~1.94 Gb and ~1.58 Gb are generated with the scaffold N50 of ~160 Mb and ~119 Mb in *C. simplicifolius* and *D. jenkinsiana*, respectively. The *C. simplicifolius* and *D. jenkinsiana* genome were predicted to harbor 51,235 and 53,342 intact protein-coding gene models, respectively. BUSCO evaluation demonstrated that the genome completeness reached 96.4% and 91.3% in the *C. simplicifolius* and *D. jenkinsiana* genome, respectively. Additionally, genome evolution revealed that the ancestor of rattans was separated from *Elaeis guineensis* at ~113 Mya.

Conclusions: To our knowledge, we present the first *de novo* assemblies of the two rattan genomes (*C. simplicifolius* and *D. jenkinsiana*). These essential data will not only provide a fundamental resource of functional genomics particularly in promoting germplasm utilization for breeding improved rattan varieties, but also serve as two reference genomes for performing comparative studies between and among different species.

Keywords: Rattan, *Calamus simplicifolius*, *Daemonorops jenkinsiana*, whole genome sequencing, genome assembly, annotation

Background

Rattan is one of the world's most important non-timber forest products and represents a major lineage of climbing palms occurring naturally in the Old World in a narrow sense [1]. A recent study indicates rattan is classified into 11 genera within the tribe of Calameae of the subfamily Calamoideae of the family Arecaceae in the world and consists of 631 species in a broad sense together with non-climbing palms in the same genera [2]. Among all genera, *Calamus* (NCBI Taxon ID:4711) and *Daemonorops* (NCBI Taxon ID:93268) were most diversified, accounting for ~65% and ~20% of rattan species [3], respectively. The two genera are also the most important material sources, producing more than 95% canes for the rattan industry. More than 5 million people depend economically on rattan and about 7 billion US dollars per year was made in the rattan industry, including domestic industry production and international trade of canes and their splitting, plaiting materials, baskets, seats and furniture [4]. With the increasing attention and the development of genetic and breeding techniques in rattan, the area of planting rattans is expected to gradually exceed that of natural rattans in recent years.

Calamus simplicifolius (NCBI Taxon ID:746888), is a deeply developed and indigenous rattan species in China, generally forming an open cluster of vigorous, unbranched stems with up to 50 m long and ~15 mm in diameter [5,6]. As a endemic rattan in Hainan Island, *C. simplicifolius* could produce the high-quality canes of medium diameter for binding and weaving in the rattan industry because of its canes with highly pliable and remarkably durable [5]. As a representative species of high climbing evergreen rattan, *Daemonorops jenkinsiana* (NCBI Taxon ID:1510057) is only one rattan species in *Daemonorops* genus and naturally grows in the lowland rain forests below 1000 m from Bangladesh, Bhutan, Cambodia, India, Laos, Myanmar, Nepal, Thailand, Vietnam to south-eastern China [2]. *D. jenkinsiana* could produce a dense cluster of vigorous stems that can be up to 50 m long and ~30 mm in diameter with internodes up to 40 cm long [6]. As the two most productive rattan species, *C. simplicifolius* and *D. jenkinsiana* are cultivated in areas with the latitude less than 23°30' N in China, i.e., Hainan island, Guangdong, Guangxi, Yunnan, Fujian and other areas of southern. Their established planting area estimate more than 1,000 ha [5] and their morphological characteristics are provided in Fig.1. However, the lack of genomic resources in rattan severely

hampered the comprehensive understanding of genetic and evolution of the rattan and in-depth performing comparative genome analyses between and among related species. Thus, we reported the two *de novo* genome assemblies of *C. simplicifolius* and *D. jenkinsiana* using the latest sequencing (Illumina and PacBio) and Hi-C mapping technology. With the available of the two chromosome-level reference genomes in rattan, many comparative genome analyses and other downstream applications will become feasible, such as the development of biomarkers, the analysis of functional genes, molecular design breeding and so on.

Data description

DNA isolation, library construction, and sequencing

The young leaves at the vegetative growth stage of *C. simplicifolius* and *D. jenkinsiana* were collected in Spring 2015 from the Research Institute of Tropical Forestry of the Chinese Academy of Forestry in the city of Guangzhou, Guangdong Province, China (N: 23º11′29″, E: 113º22′40″, 87 M). The total DNA was isolated and extracted using DNeasy Plant Mini kit (Qiagen) based on the manufacturer's instruction. Genomic DNA was purified based on the protocol of the isolation of high-molecular-weight nuclear DNA. Multiple DNA libraries were constructed as described previously [7] and were sequenced on Illumina HiSeq 4000 and PacBio Sequel platform (Table 1). Briefly, we built three libraries with different insert sizes (270 bp, 500 bp and 800 bp) for paired-end (PE) sequencing and four libraries with different insert sizes (2 kb, 5 kb, 10 kb and 20 kb) for mate-pair (MP) sequencing according to the standard Illumina protocol. We also constructed five PacBio Sequel libraries with 20 kb of insert size according to the standard PacBio protocol. After data cleaning and data preprocessing, we obtained 494.08 Gb of clean data (322.3 Gb PE reads, 93.4 Gb MP reads, and 78.38 Gb PacBio data), representing 252× coverage of the *C. simplicifolius* genome, and 426.17 Gb of clean data (244.58 Gb PE reads, 103.21 Gb MP reads, and 78.38 Gb PacBio data), representing 266 × coverage of the *D. jenkinsiana* genome, respectively.

Moreover, two Hi-C libraries were constructed for *C. simplicifolius* and *D. jenkinsiana*, respectively. We used Mbo I restriction enzyme to digest DNA after conformation fixing by

formaldehyde and repaired 5′ overhang using biotinylated residue. Following ligating blunt-end fragments in situ, the isolated DNA was reverse-crosslinked, purified and filtered for biotin-containing fragments. Subsequently, DNA fragment end repair, adaptor ligation and PCR were performed in order. Then, the standard circularization step of BGISEQ-500 was carried out and sequencing was performed on BGISEQ500 with PE100 reads. Thus, we obtained ~6.7 Gb and ~13.1 Gb of valid data after ~148 Gb and ~154 Gb of raw data were evaluated and qualified using HiC-Pro (version 2.8.0_devel) [8] in *C. simplicifolius* and *D. jenkinsiana*, respectively (Table 1).

Genome survey

The understanding of genome characteristics for a given new species, i.e., genome size and heterozygosity, facilitates customizing a specific sequencing and assembling strategy. Thus, a genome survey analysis was performed using the *k*-mer analysis described previously [9]. In our study, ~98 Gb and ~60 Gb of genome survey sequences were generated from short-insert size libraries in *C. simplicifolius* and *D. jenkinsiana*, respectively. In the data preprocessing, low-quality reads (the proportion of the base of Q<13 more than 40% in a given reads) were filtered using NGS QC Toolkit (version 2.3.3) [10] with default parameters. The FindErorrs, a script of ALLPATHS-LG (version r52488) [11], was used to predict the genome size and heterozygosity. The *k*-mer result (Fig. 2) showed the predicted genome sizes were ~1.98 Gb of *C. simplicifolius* and ~1.61 Gb of *D. jenkinsiana*, and the related heterozygosity was evaluated at 1.32%~1.52% and 1.19~1.31%, respectively. Thus, the genome survey suggested the two rattan genomes might be suitable for a hybrid sequencing strategy using Illumina and PacBio data.

Hybrid *de novo* genome assembly using Illumina, PacBio and Hi-C sequencing data

In the preprocessing of Illumina data, we filtered the low-quality reads and adaptor sequences. Thus, ~416 Gb and ~348 Gb of clean data were generated in *C. simplicifolius* and *D. jenkinsiana*, respectively. For the PacBio data, we used MECAT (release 20170627) to correct errors [12] with the following parameters: -x 0 -i 0 -t 60 -r 0.8 -a 1000 -c 5 -l 2000. Thus, we obtained ~52 Gb and ~32 Gb of corrected PacBio data in *C. simplicifolius* and *D. jenkinsiana*, respectively. Subsequently,

FALCON (version 0.3, https://github.com/PacificBiosciences/FALCON) was priority to perform assembling the initial contigs of the two rattans. As shown in Additional Table S1, the result showed two assemblies using different parameters were generated for the *C. simplicifolius* genome, i.e., 159 Mb of assembly size with 67.2 kb of contig N50 (~80% of the estimated genome size) and 153 Mb of assembly size with 66.7 kb of contig N50 (~77% of the estimated genome size). Additionally, 127 Mb of assembly size with 81.5kb of contig N50 (~79% of estimated genome size) was obtained for *D. jenkinsiana*. Thus, we assumed the incompleteness assembled scaffolds may due to inadequate sequencing depth, which contained ~26× and ~20× effective PacBio data after error correction, respectively. Therefore, according to the above investigations, we conducted hybrid *de novo* genome assembly of *C. simplicifolius* and *D. jenkinsiana* using Illumina and PacBio sequencing data. First, Platanus (version 1.2.4) [13], an *de novo* and high heterozygous genome assembler, was carried out to assemble the fragment PE reads into contigs by constructing De Bruijn Graphs with automatically optimized *k*-mer size. Second, the corrected PacBio reads and the assembled contigs were thrown into DBG2OLC (release 20150611) [14] to construct scaffolds with the parameters: DBG2OLC Contigs contig.fa LD 0 K 17 KmerCovTh 4 MinOverlap 25 AdaptiveTh 0.007 RemoveChimera 1 f scaffold.fa. Hence, we obtained ~1.92 Gb and ~1.56 Gb of initial assembly sequences in *C. simplicifolius* and *D. jenkinsiana*, respectively. Third, the assemblies were elongated by SSPACE (version 3.0) [15] using the MP reads and some gaps were filled using the Illumina and PacBio data by GapCloser (version 1.12) [16] and PBJelly (release 20150824) [17]. Thus, we obtained an assembly of 1.96 Gb, containing 5,116 scaffolds with a contig N50 length of 107 kb and a scaffold N50 of 803 kb in *C. simplicifolius*, and we also obtained another assembly of ~1.60 Gb in *D. jenkinsiana* and the N50 length was 108 kb and 784 kb for the contig and scaffold, respectively (Table 2).

Subsequently, the valid Hi-C data together with the above assembly were processed by 3D-DNA pipeline (version 170123) [18] to anchor the scaffolds onto chromosomes. Based on 4 iterations, we obtained an explicit contact pattern which inferred a pretty accurate assembly in each chromosome. As shown in Fig. 3, the contact maps were visualized by Juicerbox (version 1.5.2) [19]. The length of

the longest 12 chromosomes for the *C. simplicifolius* assembly and that of 13 chromosomes for the *D. jenkinsiana* assembly representing pseudo-chromosomes were presented in Additional Table S2. The total length of the pseudo-chromosomes accounted for 92.30% and 93.32% of *C. simplicifolius* and *D. jenkinsiana*, which with scaffold N50 of 169 Mb and 119 Mb, respectively.

Genome evaluation

Three independent methods were used to evaluate the accuracy and completeness of the *C. simplicifolius* and *D. jenkinsiana* assemblies. First, two genome features were summarized, i.e., the percentage of ambiguous bases (Ns) and GC content. The result showed a low percentage of Ns (~ 0.6% of *C. simplicifolius* and ~ 0.7% of *D. jenkinsiana*) was observed and overall GC contents (41.07% of *C. simplicifolius* and 41.78% of *D. jenkinsiana*) were similar to related transcriptomic data (41.68% of *C. simplicifolius* and 41.89% of *D. jenkinsiana*). Then, the unigenes assembled by RNA-Seq data were aligned to the assembly using BLAT (version 1.0) [20] with default parameters. The alignment results showed more than 90% sequences in one scaffold reach to a high proportion (92.89% of *C. simplicifolius* and 81.81% of *D. jenkinsiana*) (Additional Table S3). Last, the completeness of the two rattans assemblies was evaluated using BUSCO (version 3.0) [21], which quantitatively assesses genome completeness using evolutionarily informed expectations of gene content from near-universal single-copy orthologs. BUSCO results showed 96.4% of conserved BUSCO proteins (embryophyta_odb9) were detected in the *C. simplicifolius* assembly, including 3.8% of the fragment BUSCO proteins. Additionally, 87.3% and 4.0% of the conserved BUSCO proteins were identified as the complete and fragment in *D. jenkinsiana*, respectively (Additional Table S4).

Repeat annotation

Before the prediction of protein-coding gene models, transposable elements (TEs) and tandem repeats were identified in the *C. simplicifolius* and *D. jenkinsiana* assembly, respectively. We adopted two independent approaches to predict repetitive elements, i.e., homology-based annotation and *de novo* method. In the homology-based annotation, TEs were identified using RepeatMasker (v4.0.5) and

RepeatProteinMasker (v4.0.5) [22] via searching against Repbase library (release 201712) [23]. In *de novo* annotation, a *de novo* repeat library was constructed using RepeatModeler (v1.0.8, http://www.repeatmasker.org/RepeatModeler/) and LTR_FINDER [24] after eliminating contaminant and multi-copy genes. Then, RepeatMasker were performed to categorize the genome sequence against the *de novo* repeat library. Additionally, tandem repeat sequences were identified by Tandem Repeat Finder (version 4.09) [25] with the following parameters: "Match = 2, Mismatch = 7, Delta = 7, PM = 80, PI = 10, Minscore = 50 and MaxPerid = 2000". Thus, the common result showed that long terminal repeat (LTR) was the most abundant repeats as well as SINE and LINE, two non-LTR retrotransposons, were lowest proportions in the two rattan assemblies (Additional Table S5). TEs were accounted for 54.15% and 70% of the *C. simplicifolius* and of *D. jenkinsiana* assembly, respectively, and the sequence divergence of TEs indicated that the *de novo* predicted repeats were more recently-active than the Repbase predicted repeats (Fig.4).

RNA sample collection, library construction, and transcriptome assembly

The four samples of the distal cirrus with three developmental stages were collected in *C. simplicifolius* and *D. jenkinsiana*, respectively. Each sample has three biological replicates (Additional Table S6). As one part of the rattan genome project, the position of RNA sampling was consistent with that of DNA sampling. Based on the manufacturer's instructions, RNA was isolated using TRIzol Reagent Solution (Invitrogen, Carlsbad, CA, USA) and the purity and concentration were determined with a NanoDrop 2000 spectrophotometer. Reverse transcription was conducted with a Reverse Transcription System (Promega, USA). The extracted RNA was treated with RNase-free DNase I for 30 min at 37 ºC to remove the residual DNA as described previously [9] and then the pooled libraries were sequenced using the BGISEQ500 platform with 100 short PE reads. In the preprocessing of the transcriptomic data, adaptor sequences and low-quality reads were filtered using SOAPnuke (version 1.5.6, https://github.com/BGI-flexlab/SOAPnuke) with the following parameters: -n 0.001 -l 20 -q 0.4 -Q 2. The clean reads of all samples were together assembled using Trinity (version 2.0.6)[26] with the following parameters: (1) group_pairs_distance 500, (2) min_contig_length 200, (3) min_kmer_cov 2, (4) min_glue 2, (5) bfly_opts -V 5, (6) edge-thr=0.1, (7)

stderr, and (8) SS_lib_type RF. Then, the outputs of Trinity were clustered to generate a single set of non-redundant references using TGI Clustering Tool (version v2.0.6) [27] with the following parameters: (1) a minimum of 95% identity between the contigs, (2) a minimum of 35 overlapping bases, (3) a minimum of 35 scores, and (4) a maximum of 20 unmatched overhanging bases at the sequence ends. Ultimately, the assembled transcripts were divided into the two classes based on sequence similarity: clusters (prefixed with 'CL') and singletons (prefixed with 'unigene'). In each cluster, the sequence similarity regions between the transcripts were more than 70% and the transcripts were spliced isoforms from a gene or a paralogous gene. Additionally, all unigenes were used in subsequent analyses.

Gene modeling and prediction

We performed a considerate prediction of intact protein-coding gene models using three independent approaches [7], i.e., *de novo* prediction, homology-based method, and RNA-Seq approach. The repeat masked assembly was firstly annotated by AUGUSTUS (version 3.3) with default parameters [28], which was a *de novo* predictor based on the self-trained model. Via optimizing training data and multiple trainings, the results showed 85,246 and 87,613 gene models were predicted in *C. simplicifolius* and *D. jenkinsiana*, respectively. In the homology-based prediction, we used seven species as reference datasets, i.e., *Elaeis guineensis*, *Phoenix dactylifera*, *Brachypodium distachyon*, *Oryza sativa*, *Setaria italic*, *Sorghum bicolor*, and *Zea mays* (see Availability of supporting data for genome versions). Their protein sequences were downloaded for ENSEMBL database [29] and were aligned to the *C. simplicifolius* and *D. jenkinsiana* assembly using TBLASTN (version 2.2.26) [30] with a cutoff of e-value<=$1e^{-5}$, respectively. Then, the splicing patterns were generated by GeneWise (version 2.0) [31]. In the RNA-Seq analysis, HISAT2 (version 2.0.2) [32] was used to identify exon-intron splicing junctions and refine the alignment of the RNA-Seq reads to the genome. Then, we used Cufflinks (version 2.2.1) [33] to define 56,024 and 58,134 protein-coding gene models in *C. simplicifolius* and *D. jenkinsiana*, respectively (Additional Table S7).

Lastly, we integrated the evidences from the three above independent predictions using MARKER (version 2) [34]. The final prediction results showed 51,235 and 53,342 intact protein-coding gene models were predicted as a consensus gene set in *C. simplicifolius* and *D. jenkinsiana*, respectively.

Annotation evaluation and gene function prediction

We evaluated the predicted annotations using two independent methods, i.e., gene function evaluation and completeness evaluation by BUSCO. In the gene function evaluation, we assessed the agreement of predicted annotations with aligned protein evidences or on homologous proteins to closely related species by manual annotations. The result of aligned against five authoritative protein databases (Additional Table S8) indicated 5.34% and 2.89% of the predicted gene models were identified as unannotated genes in *C. simplicifolius* and *D. jenkinsiana*, respectively. These protein databases included non-redundant NCBI protein database (release 20180313) [35], SWISS-PROT (release 201801) [36], GO (release 20131030) [37], KEGG (dataset v81) [38], and InterPro (dataset v.53) [39]. Additionally, BUSCO evaluation showed 88.7% and 91.3% of conserved BUSCO proteins (embryophyta_odb9) were present in *C. simplicifolius* and *D. jenkinsiana*, respectively. Among the conserved BUSCO proteins, 76.2% and 81.2% were complete. Furthermore, the four types of non-coding RNA genes, i.e., tRNA, rRNA, miRNA and snRNA, were also predicted (Additional Table S9).

Gene family construction

OrthoMCL (version 2.0.9) [40] was used to identified orthologous genes in *C. simplicifolius*, *D. jenkinsiana* and other 7 plants, i.e., *Amborella trichopoda*, *E. guineensis*, *A. thaliana*, *B. distachyon*, *O. sativa*, *S. polyrhiza*, and *S. bicolor* (see Availability of supporting data). Among 29,240 gene families identified in entire 9 species, 40,932 and 4,1188 orthologous genes were detected in the *C. simplicifolius* and *D. jenkinsiana* genome, respectively. Approximately 6,532 (22.3%) gene families confined entire 9 species as well as 1,601 and 2,638 specific gene families were detected in *C. simplicifolius* and *D. jenkinsiana*, respectively (Fig 5).

Phylogenetic analysis

We obtained 1,244 single-copy orthologous genes derived from entire gene families, which are conserved among species to facilitate understanding the evolutionary relationship of rattan with other species. First, multiple alignments of protein sequences were conducted by MUSCLE (version 3.8.31) [41], and then CDS alignment was constructed based on the protein alignments. Subsequent, all aligned CDS sequences were concatenated to generate a super gene for each species using an in-house Perl script. Thus, we extracted the nucleotides at position 2 (phase1) of each codon to construct the phylogenetic tree using RAxML (version 8.2.3) [42] with the model "GTRGAMMA". The results showed that *C. simplicifolius* and *D. jenkinsiana* were clustered as a sister branch, which close to *E. guineensis* (Fig. 6a).

Divergence time

We used the MCMCTree program of PAML (version 4.5) [43] to estimate the divergence time among *C. simplicifolius, D. jenkinsiana* and the other 7 plants with the following parameters: "-nsample 200000 -burnin 40000". The calibration times of *O. sativa-B. distachyon, A. thaliana-S. polyrhiza* and *A. trichopoda-E. guineensis* were derived from Timetree (http://www.timetree.org). The divergence time between *D. jenkinsiana* and *E. guineensis* was ~24.2 Mya and the ancestor of rattans was separated from *E. guineensis* at ~113 Mya (Fig. 6b).

Conclusion

We reported the two chromosome-level reference genome sequences in rattan (*C. simplicifolius* and *D. jenkinsiana*) using the multiple types of sequencing data and assembly technologies. The availability of the *C. simplicifolius* and *D. jenkinsiana* genome could facilitate *de novo* genome assembling and resequencing of other species in rattan, and server as essential resources to identify regions providing a suitable resolution in an evolutionary landscape by performing comparative studies between and among different species. The two high-quality rattan genomes become easier to identify the genes involved in metabolite pathways that have potential developmental importance. Therefore, these data pave the way for extra-genomic studies in rattan and related plants.

Availability of supporting data

The datasets supporting the results of this article are available in the *Giga*DB repository [44]. All raw genomic sequence reads from BIGSEQ500, Illumina and PacBio platform, and transcriptome reads derived from multiple tissues have been uploaded and deposited in the European Nucleotide Sequence Archive (EMBL-EBI) with the project accession No. PRJEB24031 and PRJEB24829 for *C. simplicifolius* and *D. jenkinsiana*, respectively. Other analytical data in this study included *A. trichopoda* (version 1.0) downloaded from Amborella Genome Database (amborella.huck.psu.edu) and *E. guineensis* (version GCF_000442705.1) downloaded from NCBI database. Except for the above two species, other genomes were downloaded from ENSEMBL database, including *E. guineensis* (version GCF_000442705.1), *Ph. dactylifera* (version 1.0), *B. distachyon* (version 3.1), *O. sativa* (version R498), *S. italic* (version 9.0), *S. bicolor* (version 3.1), *Z. mays* (version B73_RefGen_V4), and *A. thaliana* (version: TAIR10).

Abbreviations

NCBI: National Center for Biotechnology Information; SRA: Sequence Read Archive (SRA); RNA-Seq: RNA-sequencing; BUSCO: Benchmarking Universal Single-Copy Ortholog; GO: Gene Ontology; TE: transposable element; GABR: Genome Atlas of Bamboo and Rattan; PE: paired-end; MP: mate-pair; LTR: long terminal repeat;

Additional files

Additional Table S1: Statistics of the assemblies using different assembling strategies

Additional Table S2: The chromosome-level length of Hi-C assembly in *C. simplicifolius* and *D. jenkinsiana*

Additional Table S3: Statistics of the quality assessment of the *C. simplicifolius* and *D. jenkinsiana* genomes

Additional Table S4: BUSCO evaluation of the *C. simplicifolius* and *D. jenkinsiana* genomes

Additional Table S5: Statistics of the predicted repetitive sequences in the *C. simplicifolius* and *D. jenkinsiana* genomes

Additional Table S6: Statistics of RNA libraries in transcriptome assembly

Additional Table S7: Statistics of the predicted protein-coding genes in the *C. simplicifolius* and *D. jenkinsiana* genomes

Additional Table S8: Statistics of functional annotations of the *C. simplicifolius* and *D. jenkinsiana* genomes

Additional Table S9: Statistics of the predicted non-coding RNAs in the *C. simplicifolius* and *D. jenkinsiana* genomes

Competing interests

The authors have declared that there are financial and non-financial competing interests in this study.

Funding

This work was supported by the Sub-Project of the National Science and Technology Support Plan of the Twelfth Five-Year Plan in China (No. 2015BAD04B03 and 2015BAD04B01) and Fundamental Research Funds for the International Center for Bamboo and Rattan (No. 1632017018).

Author contributions

HSZ and RSL collected the samples; JLW, HYS, SNW, HX, KBY, XRX, XMS, and JJS constructed libraries; HSZ, SBW, CHC, LFC, AQF, CZ, and QG performed the genome assembly. SJH, KH, CCS, and GYF performed the Hi-C analysis. SBW and LFC performed the genome annotation; HSZ, SBW, LFC, and XL analyzed the genome data. HSZ and SBW wrote the manuscript; HSZ, SBW, XL, ZMG and ZHJ reviewed the manuscript. All above authors have read and approved the final manuscript.

Acknowledgements

As a part of Genome Atlas of Bamboo and Rattan (GABR), we wish to acknowledge the GABR Consortium members, partners, advisors, and supporters who have helped this project run smoothly.

Reference

1. Jiang Z. Bamboo and Rattan in the World. Beijing: China Forestry Publishing House 2007.

2. International Network for Bamboo and Rattan. World Checklist of Bamboo and Rattans. Beijing: International Network of Bamboo and Rattan; 2017.

3. Larsen K. Genera Palmarum. A classification of palms based on the work of Harold E. Moore Jr. Nordic Journal of Botany. 1989;9:62–2.

4. Kumar HNK, Preethi SD, Chauhan JB. Studies on the *in vitro* propagation of Calamus travancoricus. Asian Journal of Plant Science and Research. 2012;:1–7.

5. Li R-S, Yin G-T, Yang J-C, Zou W-T. Rattan sector in Hainan Island, China: a case study. Journal of Forestry Research. 2007;18:153–6.

6. Internet EPOT. eFloras Published on the Internet. Missouri Botanical Garden, St. Louis, MO, & Harvard University Herbaria, Cambridge, MA. 2008. http://www.eoras.org. Accessed 20 Apr 2018.

7. Peng Z, Lu Y, Li L, Zhao Q, Feng Q, Gao Z, et al. The draft genome of the fast-growing non-timber forest species moso bamboo (*Phyllostachys heterocycla*). Nature Genetics. 2013;45:456–61.

8. Servant N, Varoquaux N, Lajoie BR, Viara E, Chen C-J, Vert J-P, et al. HiC-Pro: an optimized and flexible pipeline for Hi-C data processing. Genome Biology. 2015;16:259.

9. Zhao H, Sun H, Li L, Lou Y, Li R, Qi L, et al. Transcriptome-based investigation of cirrus development and identifying microsatellite markers in rattan (*Daemonorops jenkinsiana*). Scientific Reports. 2017;7:46107.

10. Patel RK, Jain M. NGS QC Toolkit: A Toolkit for Quality Control of Next Generation Sequencing Data. PloS one. 2012;7:e30619.

11. Maccallum I, Przybylski D, Gnerre S, Burton J, Shlyakhter I, Gnirke A, et al. ALLPATHS 2: small genomes assembled accurately and with high continuity from short paired reads. Genome Biology. 2009;10:R103.

12. Xiao C-L, Chen Y, Xie S-Q, Chen K-N, Wang Y, Han Y, et al. MECAT: fast mapping, error correction, and de novo assembly for single-molecule sequencing reads. Nature Methods. 2017;14:1072–4.

13. Kajitani R, Toshimoto K, Noguchi H, Toyoda A, Ogura Y, Okuno M, et al. Efficient *de novo* assembly of highly heterozygous genomes from whole-genome shotgun short reads. Genome research. 2014;24:1384–95.

14. Ye C, Hill CM, Wu S, Ruan J, Ma ZS. DBG2OLC: Efficient Assembly of Large Genomes Using Long Erroneous Reads of the Third Generation Sequencing Technologies. Scientific Reports. 2016;6:31900.

15. Hunt M, Newbold C, Berriman M, Otto TD. A comprehensive evaluation of assembly scaffolding tools. Genome Biology. 2014;15:R42.

16. Luo R, Liu B, Xie Y, Li Z, Huang W, Yuan J, et al. Erratum: SOAPdenovo2: an empirically improved memory-efficient short-read *de novo* assembler. GigaScience. 2015;4:30.

17. English AC, Richards S, Han Y, Wang M, Vee V, Qu J, et al. Mind the gap: upgrading genomes with Pacific Biosciences RS long-read sequencing technology. Liu Z, editor. PloS one. 2012;7:e47768.

18. Dudchenko O, Batra SS, Omer AD, Nyquist SK, Hoeger M, Durand NC, et al. *De novo* assembly of the *Aedes aegypti* genome using Hi-C yields chromosome-length scaffolds. Science. 2017;356:92–5.

19. Zhao H, Dong L, Sun H, Li L, Lou Y, Wang L, et al. Comprehensive analysis of multi-tissue transcriptome data and the genome-wide investigation of GRAS family in *Phyllostachys edulis*. Scientific Reports. 2016;6:27640.

20. Kent WJ. BLAT--the BLAST-like alignment tool. Genome research. 2002;12:656–64.

21. Simão FA, Waterhouse RM, Ioannidis P, Kriventseva EV, Zdobnov EM. BUSCO: assessing genome assembly and annotation completeness with single-copy orthologs. Bioinformatics. 2015;31:3210–2.

22. Tarailo-Graovac M, Chen N. Using RepeatMasker to identify repetitive elements in genomic sequences. Current Protocols in Bioinformatics. 2009;Chapter 4:Unit4.10–4.10.14.

23. Bao W, Kojima KK, Kohany O. Repbase Update, a database of repetitive elements in eukaryotic genomes. Mobile DNA. 2015;6:11.

24. Xu Z, Wang H. LTR_FINDER: an efficient tool for the prediction of full-length LTR retrotransposons. Nucleic Acids Research. 2007;35:W265–8.

25. Benson G. Tandem repeats finder: a program to analyze DNA sequences. Nucleic Acids Research. 1999;27:573–80.

26. Haas BJ, Papanicolaou A, Yassour M, Grabherr M, Blood PD, Bowden J, et al. *De novo* transcript sequence reconstruction from RNA-seq using the Trinity platform for reference generation and analysis. Nature Protocol. 2013;8:1494–512.

27. Pertea G, Huang X, Liang F, Antonescu V, Sultana R, Karamycheva S, et al. TIGR Gene Indices clustering tools (TGICL): a software system for fast clustering of large EST datasets. Bioinformatics. 2003;19:651–2.

28. Stanke M, Morgenstern B. AUGUSTUS: a web server for gene prediction in eukaryotes that allows user-defined constraints. Nucleic Acids Research. 2005;33:W465–7.

29. Zerbino DR, Johnson N, Juetteman T, Sheppard D, Wilder SP, Lavidas I, et al. Ensembl regulation resources. Database (Oxford). 2016;2016:bav119.

30. Mount DW. Using the Basic Local Alignment Search Tool (BLAST). CSH Protocol. 2007;2007:pdb.top17.

31. Birney E, Durbin R. Using GeneWise in the Drosophila annotation experiment. Genome research. 2000;10:547–8.

32. Kim D, Langmead B, Salzberg SL. HISAT: a fast spliced aligner with low memory requirements. Nature Methods. 2015;12:357–60.

33. Ghosh S, Chan C-KK. Analysis of RNA-Seq Data Using TopHat and Cufflinks. Methods in Molecular Biology. 2016;1374:339–61.

34. Holt C, Yandell M. MAKER2: an annotation pipeline and genome-database management tool for second-generation genome projects. BMC Bioinformatics. 2011;12:491.

35. O'Leary NA, Wright MW, Brister JR, Ciufo S, Haddad D, McVeigh R, et al. Reference sequence (RefSeq) database at NCBI: current status, taxonomic expansion, and functional annotation. Nucleic Acids Research. 2016;44:D733–45.

36. Boutet E, Lieberherr D, Tognolli M, Schneider M, Bansal P, Bridge AJ, et al. UniProtKB/Swiss-Prot, the Manually Annotated Section of the UniProt KnowledgeBase: How to Use the Entry View. Methods in Molecular Biology. 2016;1374:23–54.

37. Gene Ontology Consortium. The Gene Ontology (GO) database and informatics resource. Nucleic Acids Research. 2004;32:258D–261.

38. Kanehisa M, Furumichi M, Tanabe M, Sato Y, Morishima K. KEGG: new perspectives on genomes, pathways, diseases and drugs. Nucleic Acids Research. 2017;45:D353–61.

39. Finn RD, Attwood TK, Babbitt PC, Bateman A, Bork P, Bridge AJ, et al. InterPro in 2017-beyond protein family and domain annotations. Nucleic Acids Research. 2017;45:D190–9.

40. Chen F, Mackey AJ, Stoeckert CJ, Roos DS. OrthoMCL-DB: querying a comprehensive multi-species collection of ortholog groups. Nucleic Acids Research. 2006;34:D363–8.

41. Edgar RC. MUSCLE: multiple sequence alignment with high accuracy and high throughput. Nucleic Acids Research. 2004;32:1792–7.

42. Stamatakis A. RAxML version 8: a tool for phylogenetic analysis and post-analysis of large phylogenies. Bioinformatics. 2014;30:1312–3.

43. Yang Z. PAML 4: phylogenetic analysis by maximum likelihood. Molecular Biology and Evolution. 2007;24:1586–91.

44. Sneddon TP, Li P, Edmunds SC. GigaDB: announcing the GigaScience database. GigaScience. 2012;1:11.

Figure legends

Figure 1. Morphological characteristics of *C. simplicifolius* and *D. jenkinsiana*

The series pictures of A and B displayed different morphological characteristics of *C. simplicifolius* and *D. jenkinsiana*, respectively. (a1) a young *C. simplicifolius*; (a2) a middle-aged *C. simplicifolius*; (a3) a climbing *C. simplicifolius*; (a4) a mature *C. simplicifolius*; (a5) a nursery of *C. simplicifolius*; (b1) a young *D. jenkinsiana*; (b2) a young forest of *D. jenkinsiana*; (b3) a nursery of *D. jenkinsiana*; (b4) leaves of *D. jenkinsiana*; (b5) inflorescences of *D. jenkinsiana*; (b6) young fruits of *D. jenkinsiana*. All the pictures were taken by Prof. Rongsheng Li.

Figure 2. Evaluation of the genome size of *C. simplicifolius* and *D. jenkinsiana* by 17-mer analyses

The distribution of 17-mer depth of high-quality reads. Approximately 98 Gb and 60 Gb of sequencing reads from short-insert size libraries in *C. simplicifolius* (red line) and *D. jenkinsiana* (blue line), respectively, were split into 17 bp in length (17-mers) to plot the frequency (depth) of those 17-mers. The X-axis represents the sequencing depth and the Y-axis represents the frequency of those 17-mers at a given sequencing depth. Genome size was estimated according to the distribution. The frequency exhibits a bi-modality due to the heterozygosity.

Figure 3. The Hi-C contact map of the *C. simplicifolius* (a) and *D. jenkinsiana* genome (b)

Figure 4. The distribution of the sequence divergence rate of different TE types in the *C. simplicifolius* (a) and *D. jenkinsiana* (b) genome

Figure 5. Clusters of the orthologous and paralogous gene families in *C. simplicifolius*, *D. jenkinsiana* and other 7 full-sequenced plants using OrthoMCL

Figure 6. The phylogenetic tree and divergence times among *C. simplicifolius*, *D. jenkinsiana*, and other 7 plants

(a). The phylogenetic tree was constructed by RAxML using all single-copy genes in the 9 species and the divergence time was estimated using the MCMCTree programmer in the PAML software package. (b). The number on the nodes are divergence times and the red nodes indicate the calibration times.

Table 1: Statistics of the clean data of the *C. simplicifolius* and *D. jenkinsiana* genomes

Sequencing Platform	Insert Size	*C. simplicifolius*			*D. jenkinsiana*		
		Reads Length (bp)	Total Data (Gb)	Sequence Depth (X)*	Reads Length (bp)	Total Data (Gb)	Sequence Depth (X)*
Illumina	270 bp	150	160.9	82.09	150	98.21	61.38
	500 bp	125	60.2	30.71	125	56.9	35.56
	800 bp	125	101.2	51.63	125	89.47	55.91
	2 Kb	49	22.8	11.63	49	33.08	20.67
	5 Kb	49	16.4	8.37	49	22.1	13.81
	10 Kb	49	26.8	13.67	49	32.63	20.39
	20 Kb	49	27.4	13.98	49	15.4	9.6
PacBio	20 Kb	9,079**	78.38	39.99	9,131**	78.38	48.75
Hi-C	N.A.	100	6.7	3.42	100	13.1	8.19
Total			500.78	255.5		439.27	274.26

*Read length of PacBio means an average length

**Sequencing depth was calculated based on 1.98 Gb of the *C. simplicifolius* genome and 1.61 Gb of the *D. jenkinsiana* genome

Table 2: Statistics of the final assembly of the *C. simplicifolius* and *D. jenkinsiana* genomes

**Items**		***C. simplicifolius***		***D. jenkinsiana***	
		**hybrid assembly***	**Hi-C assembly**	**hybrid assembly***	**Hi-C assembly**
**Contig**	Number	28,010	29,973	4,398	27,631
	Size (bp)	1,893,606,800	1,923,260,127	1,550,219,532	1,570.849,893
	N50 (bp)	107,998	99,304	108,975	59,562
	N90 (bp)	29,880	28,872	30,941	25,720
**Scaffold**	Number	5,116	5,189	2,136	4,862
	Size (bp)	1,960,809,755	1,935,580,712	1,608,994,761	1,582,020,714
	N50 (bp)	803,014	160,072,219	784,425	119,093,744
	N90 (bp)	212,786	93,668,489	206,315	61,330,142
**Total number**	>3 kb	5,116	5,181	4,397	4,853
	>5 kb	5,106	5,141	4,387	4,797
**The longest sequence in scaffolds (bp)**		4,035,697	220,588,937	4,970,540	162,635,149
**The shortest sequence in scaffolds (bp)**		3,071	1,286	2,171	719
**Ns ratio (%)**		3.4	0.6	3.6	0.7
**GC ratio (%)**		39.41	41.07	39.83	41.78

*hybrid assembly means hybrid *de novo* assembly using Illumina and PacBio data

Fig.1

Click here to download Figure Fig1.Morphlogical features.pdf

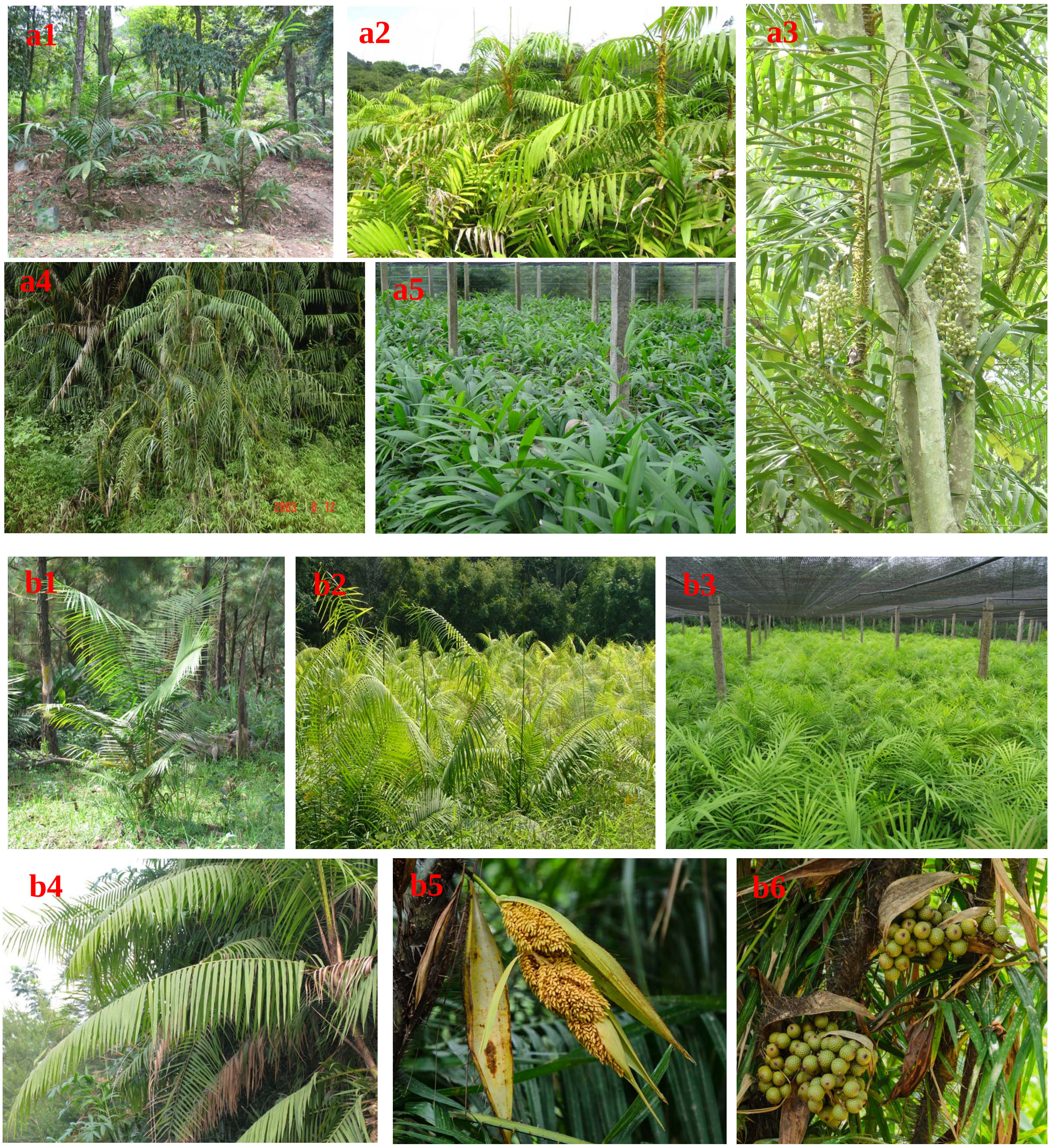

Click here to download Figure Fig2.K-mer frequence distribution.pdf

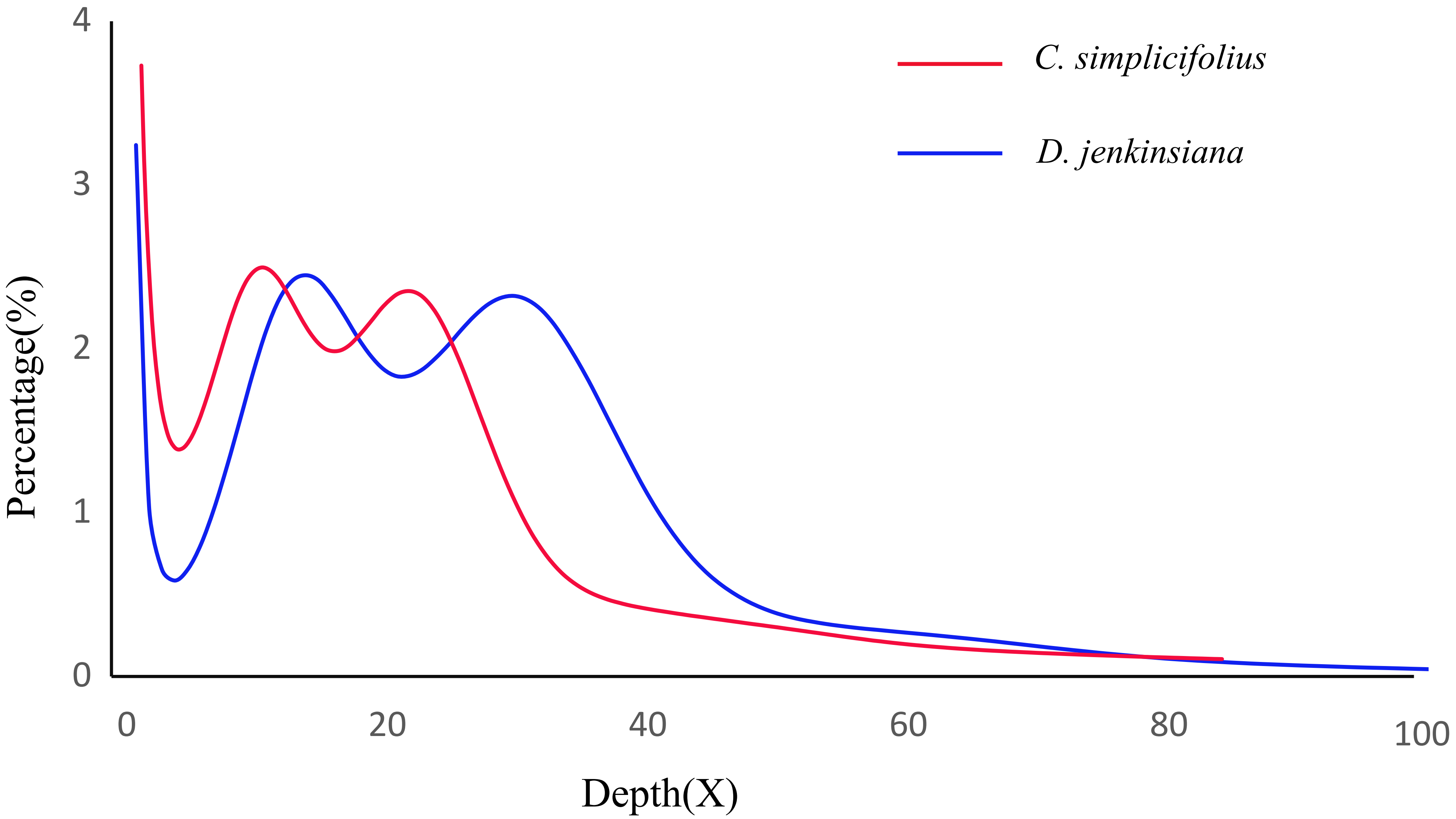

Fig.3

Click here to download Figure Fig3.Hi-Cpdf.pdf

a

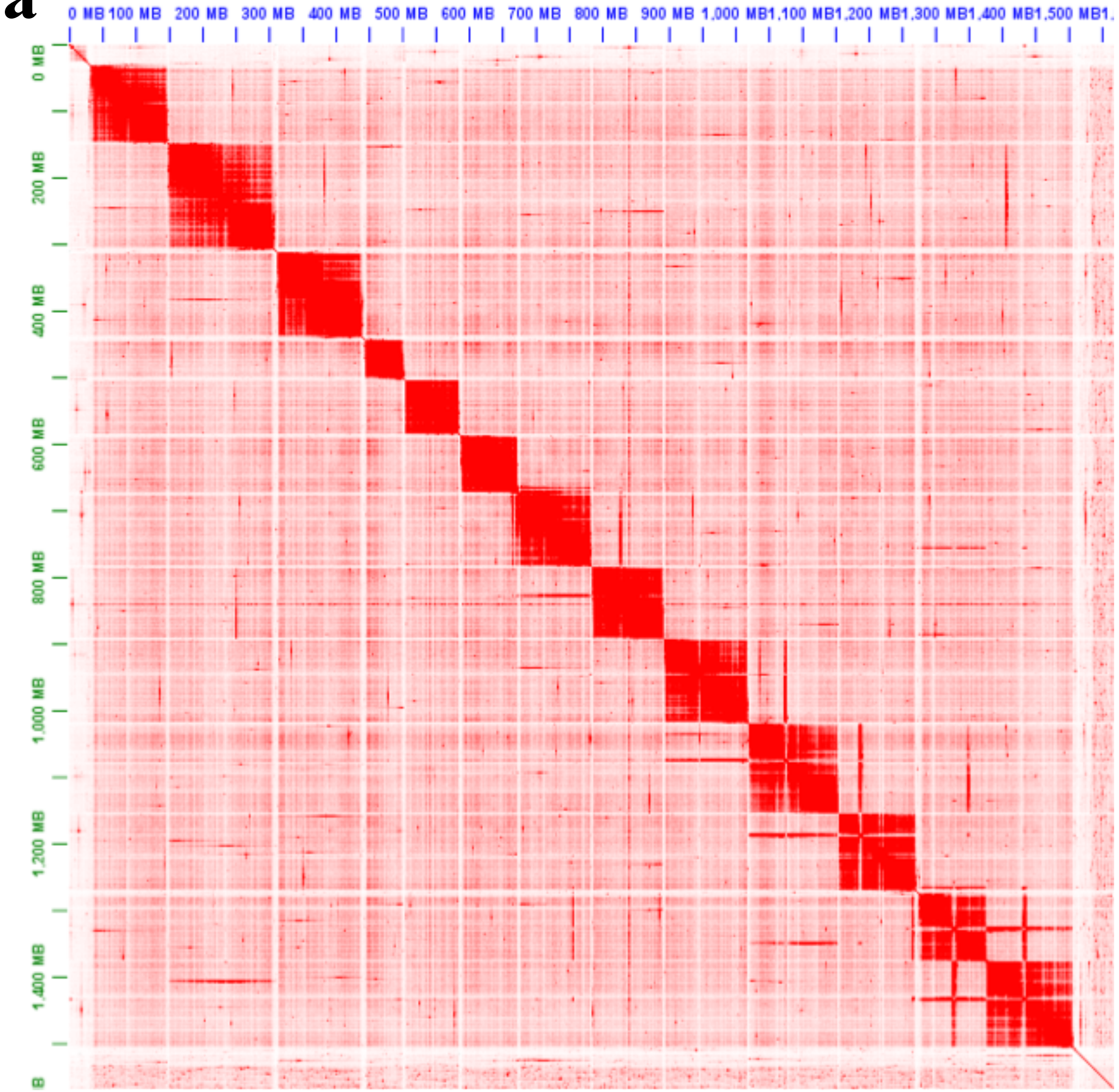

b

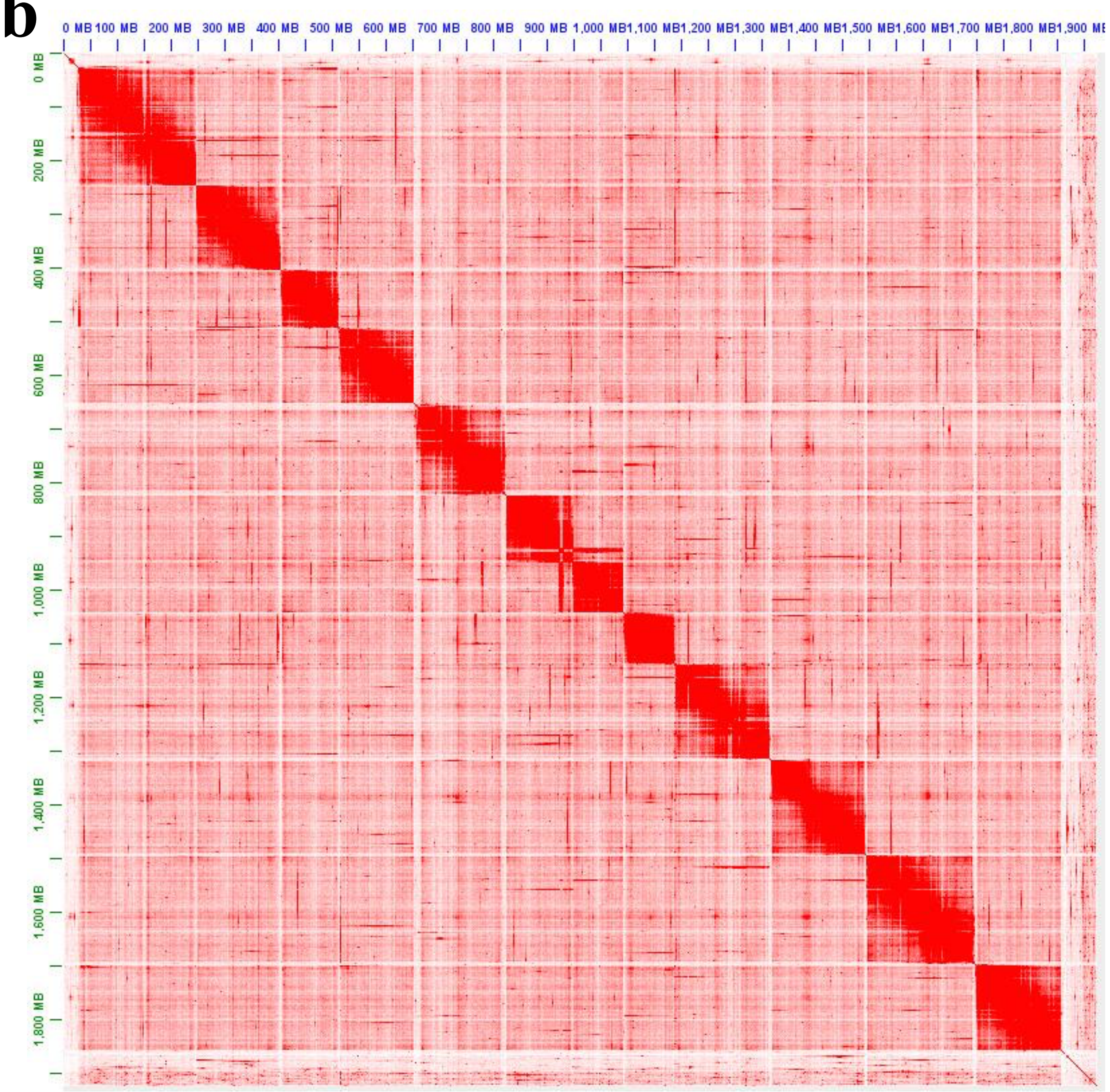

Fig.4

Click here to download Figure Fig4.Divergence rate distribution of different types of TE.pdf

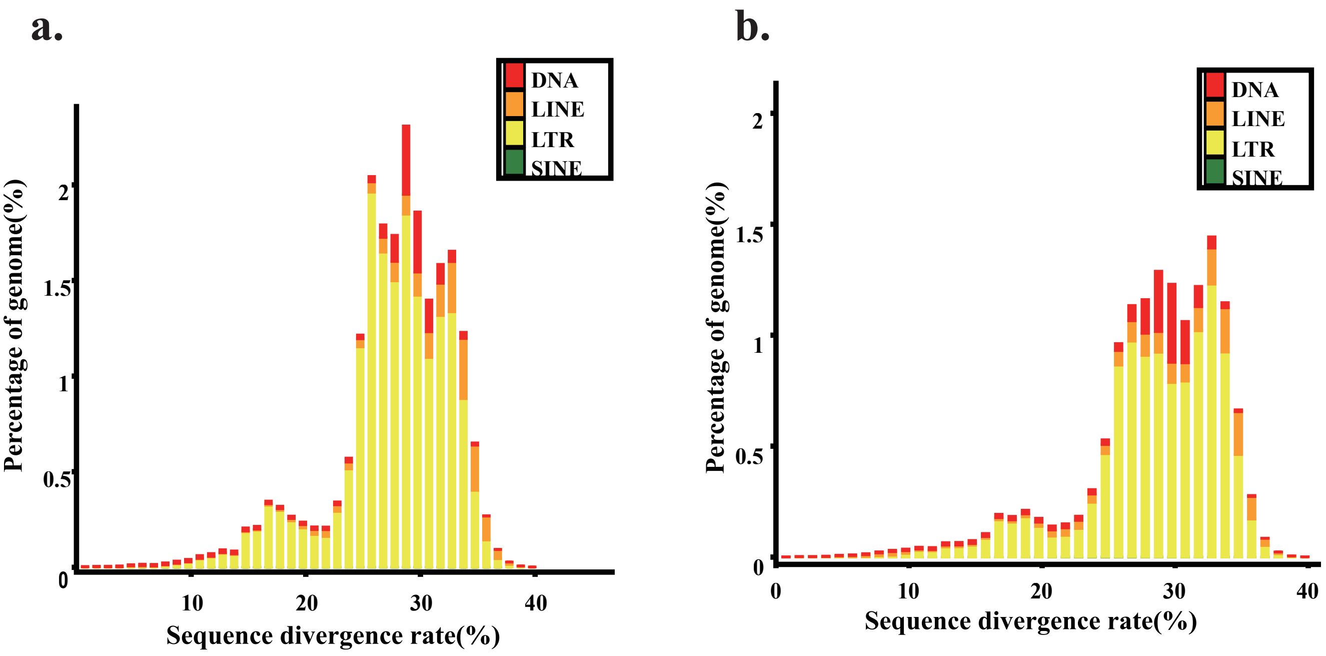

Fig.5

Click here to download Figure Fig5.ortholog.genefamily.pdf

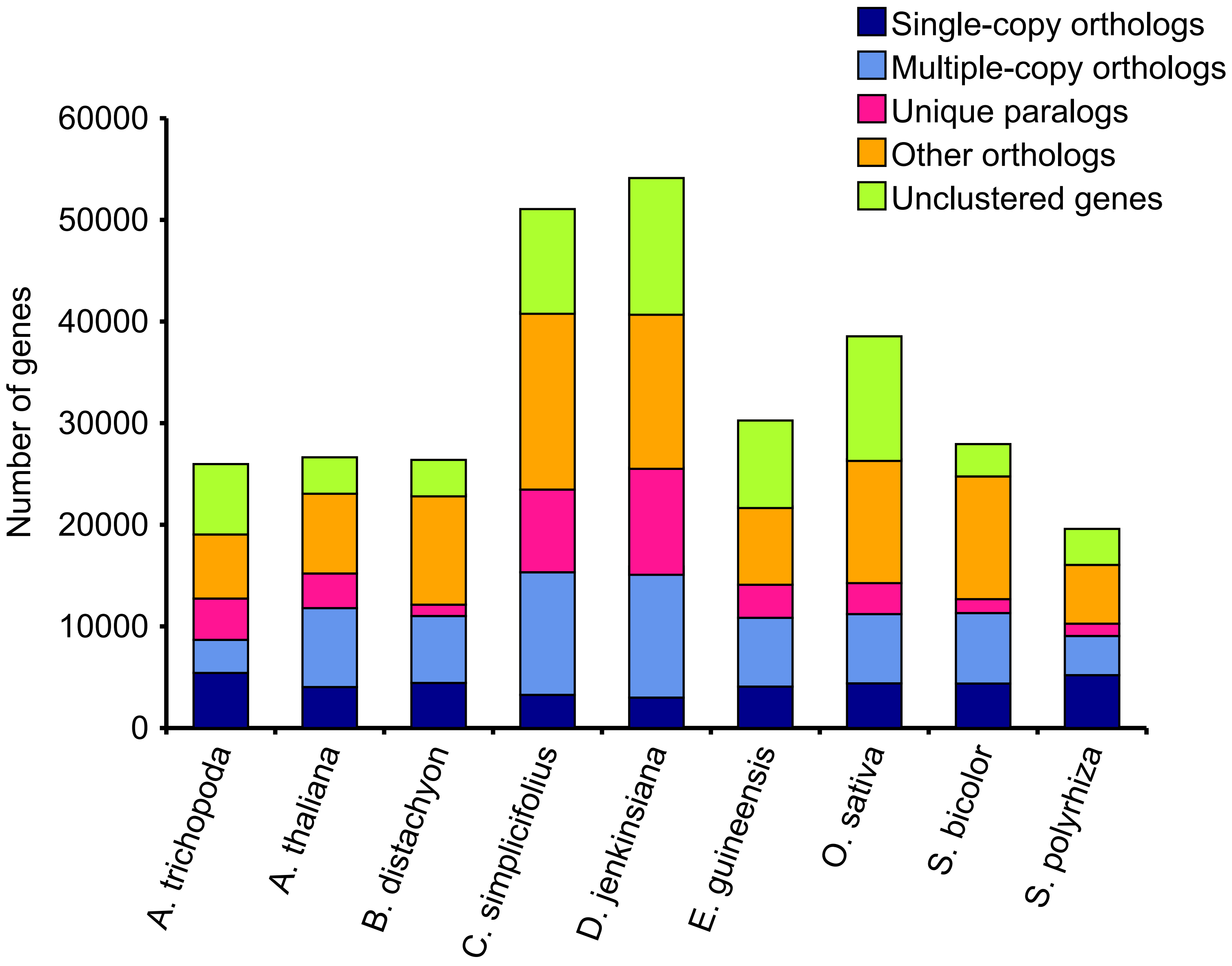

Fig.6

Click here to download Figure Fig6.phylogenetic and divergence time.pdf

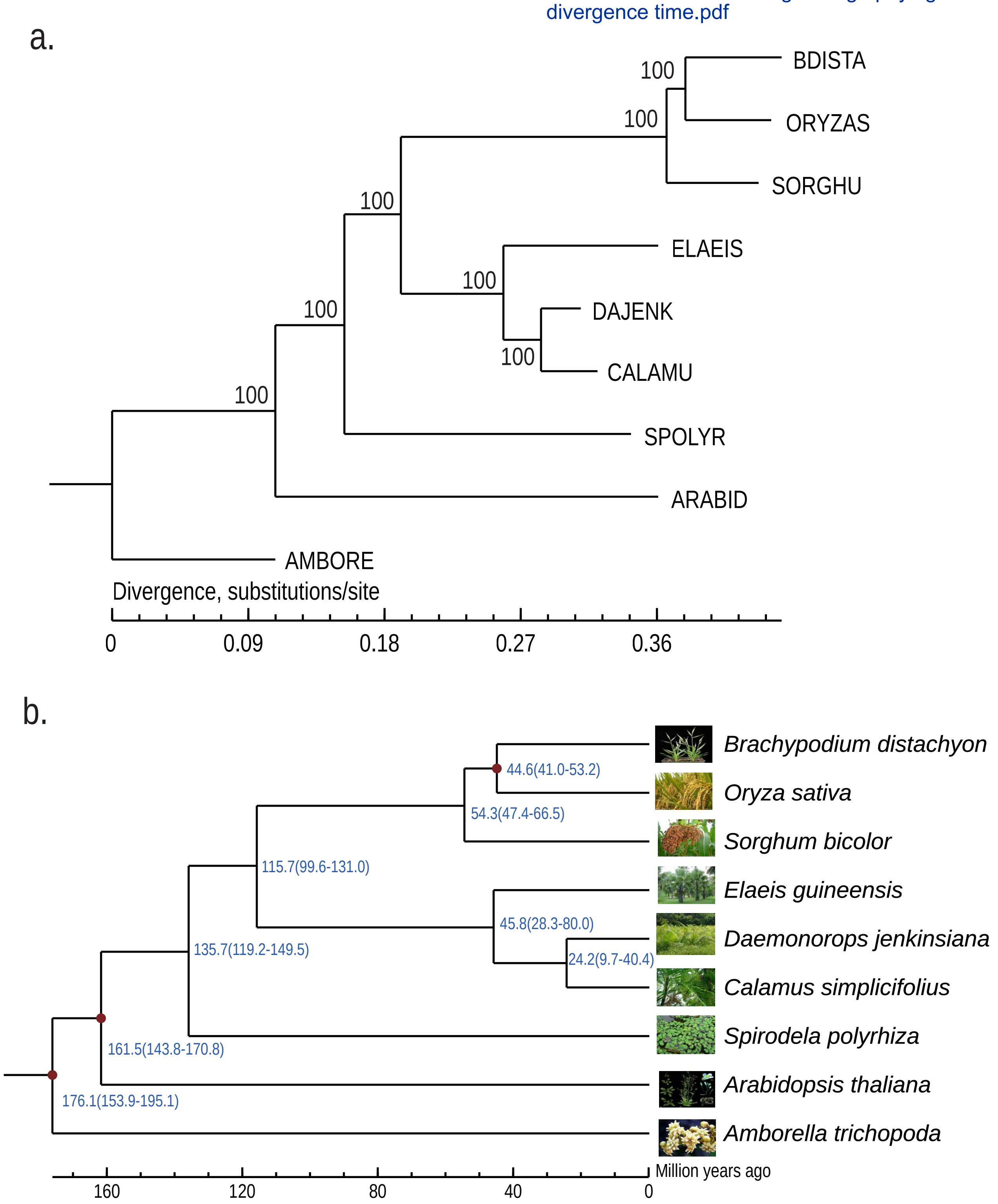

Additional File

Click here to access/download
Supplementary Material
Additional Files.xlsx

Click here to access/download
Supplementary Material
Response Letter-rattan-0508.docx
