# Supplementary material for: The chromosome-level genome assemblies of two rattans (Calamus simplicifolius and Daemonorops jenkinsiana)
Source: Gigascience. 2018 Aug 7;7(9):giy097. doi: 10.1093/gigascience/giy097 (PMC6117794; doi:10.1093/gigascience/giy097)
Supplement: GIGA-D-18-00152_Revision_1.pdf [file giy097_giga-d-18-00152_revision_1.pdf]

GigaScience

The chromosome-level genome assemblies of two rattans (Calamus simplicifolius and Daemonorops jenkinsiana)

--Manuscript Draft--

**Manuscript Number:**	GIGA-D-18-00152R1
**Full Title:**	The chromosome-level genome assemblies of two rattans (Calamus simplicifolius and Daemonorops jenkinsiana)
**Article Type:**	Data Note
**Funding Information:**	the Sub-Project of the National Science and Technology Support Plan of the Twelfth Five-Year Plan in China (2015BAD04B03) — Prof. Hansheng Zhao
**Abstract:**	Background<br><br>Calamus simplicifolius and Daemonorops jenkinsiana are two representative rattans, as the most significant material sources for the rattan industry. However, the lack of reference genome sequences is a major obstacle for basic and applied biology on rattan.<br><br>Findings<br><br>We rendered the two chromosome-level genome assemblies of C. simplicifolius and D. jenkinsiana using the Illumina, PacBio, and Hi-C sequencing data. A total of ~730 Gb and ~682 Gb of raw data covered the predicted genome length (~1.98 Gb of C. simplicifolius and ~1.61 Gb of D. jenkinsiana) to ~ 372× and ~ 426× read depth, respectively. The two de novo genome assemblies of ~1.94 Gb and ~1.58 Gb are generated with the scaffold N50 of ~160 Mb and ~119 Mb in C. simplicifolius and D. jenkinsiana, respectively. The C. simplicifolius and D. jenkinsiana genome were predicted to harbor 51,235 and 53,342 intact protein-coding gene models, respectively. BUSCO evaluation demonstrated that the genome completeness reached 96.4% and 91.3% in the C. simplicifolius and D. jenkinsiana genome, respectively. Genome evolution showed that four Arecaceae plants were located in a cluster and the divergence time between the two rattans was ~19.3 Mya. Additionally, we genome-widely identified 193 and 172 genes involved in the lignin biosynthesis pathway in C. simplicifolius and D. jenkinsiana, respectively.<br><br>Conclusions<br><br>We present the first de novo assemblies of the two rattan genomes (C. simplicifolius and D. jenkinsiana). These essential data will not only provide a fundamental resource of functional genomics particularly in promoting germplasm utilization for breeding improved rattan material property, but also will serve as two reference genomes for performing comparative studies between and among different species.
**Corresponding Author:**	Hansheng Zhao<br>International Center for Bamboo and Rattan<br>Beijing, Beijing CHINA
**Corresponding Author Secondary Information:**	
**Corresponding Author's Institution:**	International Center for Bamboo and Rattan
**Corresponding Author's Secondary Institution:**	
**First Author:**	Hansheng Zhao
**First Author Secondary Information:**	
**Order of Authors:**	Hansheng Zhao

	Songbo Wang
	Jiongliang Wang
	Chuanhai Chen
	Shijie Hao
	Lianfu Chen
	Benhua Fei
	Kai Han
	Rongsheng Li
	Chengcheng Shi
	Huayu Sun
	Sining Wang
	Hao Xu
	Kebin Yang
	Xiurong Xu
	Xuemeng Shan
	Jingjing Shi
	Aiqin Feng
	Guangyi Fan
	Xin Liu
	Shancen Zhao
	Chi Zhang
	Qiang Gao
	Zhimin Gao
	Zehui Jiang
**Order of Authors Secondary Information:**	
**Response to Reviewers:**	Scott Edmunds<br>Executive Editor<br>GigaScience<br><br>3 Jun 2018<br><br>Dear Dr. Scott,<br><br>Re: Manuscript reference No. GIGA-D-18-00152R1<br>Please find attached a revised version of our manuscript “The chromosome-level genome assemblies of two rattans (Calamus simplicifolius and Daemonorops jenkinsiana)”, which we would like to resubmit for publication as a research article in GigaScience.<br><br>The comments of two Reviewer were highly insightful and enabled us to greatly improve the quality of our manuscript. In the following pages are our point-by-point responses to each of the comments and suggestions of the Reviewer.<br><br>Revisions in the text are shown using red highlight. In accordance with the two reviewers’ suggestions, we carefully revised our manuscript, e.g., re-assembling the two genomes and adding the analyses of GO and lignin biosynthesis pathway. We hope that the new submission and our accompanying responses will be sufficient to make our manuscript suitable for publication in GigaScience.

Powered by Editorial Manager® and ProduXion Manager® from Aries Systems Corporation

We shall look forward to hearing from you at your earliest convenience.

Yours sincerely,

Prof. Hansheng Zhao
Address: No. 8, Fu Tong Dong Da Jie, Chaoyang District, Beijing 100102, P.R. China
Tel: +86-010-8478 9804
Fax: +86-010-8478 9802
E-mail: zhaohansheng@icbr.ac.cn

Responses to the comments of Reviewer #1
This manuscript provides two genomes for plant research, which is valuable. However, the data analysis still needs to be improved before this resource is released.

1. The analysis of genome sizes was not done comprehensively. The genome sizes were critical for the result, including the evaluation of assembly completeness. The module "FindErrors" in AllPATHS-LG was designed to correct errors in sequencing reads by counting kmers. I would suggest the authors to adopt multiple tools with statistics models to estimate genome size and compare the results. Besides this, it is also not clear how the genome size was calculated from the figure 2.

Response: Thank you for this excellent suggestion. We are sorry for the confused description of “FindErorrs”. Indeed, a script of ErrorCorrrectReads.pl in ALLPATHS-LG was used to correct reads by counting K-mer and then three output files of K-mer frequents (.kspec) were generated. Subsequently, the .kspec files could be analyzed using a script of KmerSpectrumPlot.pl in ALLPATHS-LG based on K-mer statistics to evaluate some genome features including the genome size. Additionally, we had also performed another three methods to access the genome sizes, including GCE (Genome Characteristics Estimation, released 20150107, ftp://ftp.genomics.org.cn/pub/gce), JELLYFISH (version 2.0) [1] and flow cytometry (only for Calamus simplicifolius) (see Tables S1-2 and Figs. S1-2). According to your suggestion, we have re-evaluated the genome size on the basis of the combination from above methods of estimating the genome size. Furthermore, we have revised the related descriptions and provided Table S2 to explain the K-mer calculation in Figure 2, as follows:

“The genome size was estimated using four independence methods, i.e., a script of KmerSpectrumPlot.pl in ALLPATHS-LG (version r52488), GCE (Genome Characteristics Estimation, released 20150107, ftp://ftp.genomics.org.cn/pub/gce), JELLYFISH (version 2.0) [1] and flow cytometry (only for Calamus simplicifolius) (Additional Tables S1-2 and Fig. S1).”

2. It's a little surprising to see PacBio reads only delivered assembly with N50 ~60kb. Even though these two genomes have relatively high repeat content, assemblies of PacBio reads still worth another try. The author first used MECAT to correct reads, which may lose some coverage. Both MECAT and FALCON are assembly pipeline, it would make more sense to run them separately. Canu should also be tested.

Response: Thank you for this excellent suggestion. The evaluation showed high heterozygosity between the two rattans (1.32%~1.52% for C. simplicifolius and 1.19~1.31% for D. jenkinsiana). According to our experience of genome assembly, we found that PacBio data has the advantage of assembling high repetitive genome, but they are not very effective in assembling highly heterozygous ones. In our study, we had used ~20 X of clean data of PacBio after the read correction for ~40 X of raw data. Also, we had tried to assembly genome only using PacBio data but obtained bad performances (Table S3), which may be due to highly heterozygous genomes and/or less valid data of PacBio. On the other hand, the two software, MECAT and Canu, have similar pipelines and they were not good at highly heterozygous genomes,

Powered by Editorial Manager® and ProduXion Manager® from Aries Systems Corporation

according to their software's manual. We had assessed MECAT using the rattan data but also obtained a bad performance (Table S3). Taken together, we adopted a hybrid assembly strategy including Illumina and PacBio data. Additionally, the different assembly strategies we tested were provided in Table S3.

3. PacBio reads were used for Gap-filling. This will request a polish process to correct the sequencing error. Without this step, the current gene annotation is not accurate, which was largely affected by errors in the sequence.

Response: Thank you for this excellent suggestion. We had provided a polish process before the SSPACE process. The polish process referred to consensus analysis of DBG2OLC and obtained relatively good performances (Table S4), which were contributed to enhance the quality of the genome assembly and reduce errors in the SSPACE process. Additionally, no gap was detected in WGS assembly, which has less impact on subsequent gene annotations. Therefore, a polish process after gap-filling was unavailable in our study and we have revised the related context, as follows:

"Third, a polish process before the SSPACE process referred to consensus analysis of DBG2OLC (Table S4), which were contributed to enhance the quality of the genome assembly and reduce errors in the SSPACE process."

4. The author only used Fig. 3 to show the Hi-C data agreed with the sequence assembly very well. This is definitely not enough. The whole genome plot has limited resolution to find the conflict between the Hi-c links and the sequence. The author should have a detailed comparison of them to evaluate the assembly quality. Then do the scaffolding after resolve conflicts.

Response: Thank you for this excellent suggestion. Resolving the conflicts is very important for chromosome-level scaffolding. In our study, we used 3D-DNA pipeline [2], which has contained considered assessing steps for bad links to produce credible Hi-C assembly. We performed the pipeline and generated the whole genome Hi-C heat map which is commonly used to show an overview of Hi-C contacts on the longest chromosome-level scaffolds. According to your suggestion, we complementally checked the Hi-C contacts and calculated average link number along genomic distance for each longest chromosome-level scaffold to evaluate the assembly quality, and made revision by scaffolding after removing obvious conflict sequences, which have strong Hi-C links of inter-chromosome but take less relationships of intra-chromosome (hic_scaffold_1 and hic_scaffold_4 in C. simplicifolius genome and hic_scaffold_10, hic_scaffold_11, hic_scaffold_12 and hic_scaffold_13 in D. jenkinsiana genome). We have updated Figure 2 and presented one modified scaffold from the two genomes respectively as an example, as well as added the distribution of Hi-C contacts along the genomic distance, which is normally decayed.

Responses to the comments of Reviewer #2
In general, the authors have successfully performed a good if rather basic analysis for a genome paper. However, as outlined below, there are several issues that need to be addressed before the paper can be suitable for publication.

1. Unfortunately, there is a very little about any potential routes for application of the data presented. The rattans discussed in the paper are not really conventional crops in the sense of being grown in plantations and with a history of being bred for specific agronomic traits. Therefore, it is not clear how the genomic information presented here can be applied in the real world. The authors can address this point by outlining a more extensive rationale for the work in the Intro and then by discussing possible specific genes/traits that could be of interest for improving rattan species as biological resources.

Powered by Editorial Manager® and ProduXion Manager® from Aries Systems Corporation

Response: Thank you for this excellent suggestion. According to your suggestion, we have added the related description, as follows:

"C. simplicifolius and D. jenkinsiana have various applications and enormous developed potentials, and they are highly interesting mainly because of their canes with highly pliable and remarkably durable. Molecular breeding technology facilitate to meet growing requirements of the quantity and quality. However, the lack of genetic architecture underlying rattan's important traits severely hampered the comprehensive understanding of molecular biology in scientific research and actual production and in-depth performing comparative genome analyses between and among related species. Thus, we reported the two de novo genome assemblies of C. simplicifolius and D. jenkinsiana using the latest sequencing (Illumina and PacBio) and Hi-C mapping technology. With the available of the two chromosome-level reference genomes in rattan, many comparative genome analyses and other downstream applications will become feasible, such as the development of biomarkers, the analysis of functional genes, molecular design breeding and so on. Additionally, a high-quality genome assembly of two rattans facilitates genomic, transcriptomic, and metabolomics analyses of the material traits. As possible specific genes that could be of interest for improving material property, the lignin gene family members are identified in rattan. These studies lay the foundation for future research to understand and utilize the genes that determine rattan quality and its diversity within rattan germplasm."

2. As the authors already have the genome and transcriptome data, it is important for the authors to enlarge and be more specific in highlighting the contribution of this paper in their conclusions - possibly along the lines of "elucidation of this genome should be make it easier to identify genes involved in metabolite pathways that have potential developmental importance"

Response: Thank you for this excellent suggestion. We have added the section: "Genome- wide identification of gene families involved in the lignin biosynthesis pathway", as follows:

"Genome-wide identification of gene families involved in the lignin biosynthesis pathway

Lignin is a class of complex aromatic heteropolymers of monolignols that encrusts and interacts with the cellulose/hemicellulose matrix of the secondary cell wall. The aromatic lignin polymers are commonly composed of three monolignols, i.e., p-hydroxyphenyl (H), vanillin (G), and syringaldehyde (S)[3]. Thus, we performed genome-wide identification of 13 gene families involved in the lignin biosynthesis pathway of rattan using the 8 genomes, i.e., A. thaliana, B. distachyon, O. sativa, S. bicolor, Phyllostachys edulis, Populus trichocarpa, D. jenkinsiana, and C. simplicifolius. Most genome sequences (A. thaliana, B. distachyon, O. sativa, S. bicolor, and Populus trichocarpa) were downloaded from the ENSEMBL database [4]. The genome sequences of Ph. edulis was downloaded from the Bamboo Genome Database [5]. According to vast literature-based investigations, 140 genes from the lignin biosynthetic pathway was collected from experimentally validation of previous studies (Additional Table S14), and then, these known genes were used as the query sequences for further identification. A BLAST search and domain analysis as described previously [54] was used in the genome-wide identification. Briefly, we performed standard protein BLAST searches (version 2.2.26) against all genome sequences including two rattans using the coding sequence of the known genes with the following cut-off values: E-value <1e-10; identity >40%; and coverage rate >95% query sequence. The filtered sequences were subsequently analysed by hmmsearch (version 3.1b2) using the Pfam-A.hmm database (released 2017/03/31). Consequently, unclear sequences with incomplete domains were discarded by manual correction. The result showed the expansion of most families was detected in two rattans (Additional Table S15). Each gene contained multiple copies with an average of ~15 and ~13 copies per family in C. simplicifolius and D. jenkinsiana, respectively. The total size of the gene families in the lignin biosynthesis pathway contained 193 and 172 gene copies in C. simplicifolius and D. jenkinsiana, respectively. Peroxidase (POD), as the most copy number, was detected both in the two rattans. As the least copy number, phenylalanine ammonia-lyase (PAL) was identified in C. simplicifolius and coumarate

3-hdroxylase (C3H) and cinnamate 4-hydroxylase (C4H) were detected in D. jenkinsiana. The expansion of the lignin biosynthesis genes in rattan could be due to the occurrence of whole genome duplication (WGD) event, since WGD could provide more gene copies, which facilitated evolving the genes with new functions [6].”

3. We also suggest that the authors do some basic gene ontology analysis to discover how and which genes involved in key pathways of specific interest.

Response: Thank you for this excellent suggestion. We have added the GO and KEGG analysis of rattan specific-gene families, as follows:

“Additionally, the result showed that 637 gene families were specific to the rattans. The rattan species-specific gene families were enriched in gene ontology (GO) categories related to component membrane, transcription factor activity (Table S13) and in KEGG pathways related to plant-pathogen interaction, plant hormone signal transduction (Table S14).”

4. In this paper, the authors attempt to identify gene families for two genomes. But the gene family construction should be described in more detail to explain how the they identified the 29,240 gene families. Also in Figure 5 the authors should define what they mean by "unique paralogs" and also define "other orthologs". In general they should provide data and more detailed discussion for Figure 5.

Response: Thank you for this excellent suggestion. We have revised the part by providing more details, as follow:

“In our study, we performed a pairwise sequence comparison to genome-widely predict orthologous genes. The method is faster and generally deals with larger amounts of data. As a popular BLAST-based approach, OrthoMCL (version 2.0.9) [41] was used to identified orthologous genes in C. simplicifolius, D. jenkinsiana with the following BLASTP parameters: an E-value cutoff of 1e-5 and a percent match cut-off of 80 (i.e., query and match had to overlap on more than 80% of the query and match sequence length). Markov Chain Clustering was also used with a default inflation parameter in an all-to-all BLASTP analysis of entries for other 8 plants, i.e., Amborella trichopoda, E. guineensis, A. thaliana, B. distachyon, O. sativa, S. polyrhiza, P. dactylifera and S. bicolor (see Availability of supporting data).”

“Figure 5. Clusters of the orthologous and paralogous gene families in C. simplicifolius, D. jenkinsiana and other 7 full-sequenced plants using OrthoMCL.
Single-copy orthologs and multiple-copy orthologs represent one copy and more one copies from the gene families in each of selected species, respectively. Unique paralogs represent unique gene related by duplication within a given genome. Other orthologs represent gene duplication events internal to the overall set, but basal more than two of the compared species.”

5. Finally, since there is a P. dactylifera genome available and is in the same family as rattan and oil palm, it will be good to be including these two palm species in the phylogenetic analysis and calculate their possible divergence times.

Response: Thank you for this excellent suggestion. We have added the P. dactylifera genome for reconstructing phylogenetic relationship and revised the subsequent analysis based on the new result (Fig.4a).

Reference:
1. Marçais G, Kingsford C. A fast, lock-free approach for efficient parallel counting of occurrences of k-mers. Bioinformatics. 2011;27:764–70.

Powered by Editorial Manager® and ProduXion Manager® from Aries Systems Corporation

	2. Dudchenko O, Batra SS, Omer AD, Nyquist SK, Hoeger M, Durand NC, et al. De novo assembly of the Aedes aegypti genome using Hi-C yields chromosome-length scaffolds. Science. 2017;356:92–5.<br>3. Martone P, Estevez J, Lu F, Ruel K, Denny MW, Somerville C, et al. Discovery of lignin in seaweed reveals convergent evolution of cell-wall architecture. Curr. Biol. 2009;19:169–75.<br>4. Kersey P, Allen J, Allot A, Barba M, Boddu S, Bolt B, et al. Ensembl Genomes 2018: an integrated omics infrastructure for non-vertebrate species. Nucleic Acids Res. 2018;46:D802–808.<br>5. Zhao H, Peng Z, Fei B, Li L, Hu T, Gao Z, et al. BambooGDB: a bamboo genome database with functional annotation and an analysis platform. Database (Oxford). 2014;2014:bau006.<br>6. Taylor J, Raes J. Duplication and divergence: the evolution of new genes and old ideas. Annu. Rev. Genet. 2004;38:615–43.
**Additional Information:**	
**Question**	**Response**
Are you submitting this manuscript to a special series or article collection?	No
**Experimental design and statistics**<br><br>Full details of the experimental design and statistical methods used should be given in the Methods section, as detailed in our Minimum Standards Reporting Checklist. Information essential to interpreting the data presented should be made available in the figure legends.<br><br>Have you included all the information requested in your manuscript?	Yes
**Resources**<br><br>A description of all resources used, including antibodies, cell lines, animals and software tools, with enough information to allow them to be uniquely identified, should be included in the Methods section. Authors are strongly encouraged to cite Research Resource Identifiers (RRIDs) for antibodies, model organisms and tools, where possible.<br><br>Have you included the information requested as detailed in our Minimum Standards Reporting Checklist?	Yes
**Availability of data and materials**<br><br>All datasets and code on which the conclusions of the paper rely must be either included in your submission or deposited in publicly available repositories (where available and ethically	Yes

appropriate), referencing such data using a unique identifier in the references and in the “Availability of Data and Materials” section of your manuscript.<br><br>Have you have met the above requirement as detailed in our Minimum Standards Reporting Checklist?	

Powered by Editorial Manager® and ProduXion Manager® from Aries Systems Corporation

Revision1

Click here to download Manuscript Manuscript_Rattans-R1-0602.docx

The chromosome-level genome assemblies of two rattans (*Calamus simplicifolius* and *Daemonorops jenkinsiana*)

Hansheng Zhao[1#], Songbo Wang[2#], Jiongliang Wang[1#], Chuanhai Chen[2#], Shijie Hao[3], Lianfu Chen[1], Benhua Fei[1], Kai Han[3], Rongsheng Li[4], Chengcheng Shi[3], Huayu Sun[1], Sining Wang[1], Hao Xu[1], Kebin Yang[1], Xiurong Xu[1], Xuemeng Shan[1], Jingjing Shi[1], Aiqin Feng[2], Guangyi Fan[3], Xin Liu[3], Shancen Zhao[2], Chi Zhang[2], Qiang Gao[2*], Zhimin Gao[1*], and Zehui Jiang[1*]

[1] State Forestry Administration Key Open Laboratory on the Science and Technology of Bamboo and Rattan, Institute of Gene Science for Bamboo and Rattan Resources, International Center for Bamboo and Rattan, Futongdong Rd, WangJing, Chaoyang District, Beijing 100102, China;

[2] BGI Genomics, BGI-Shenzhen, Building NO.7, BGI Park, No. 21 Hongan 3rd Street, Yantian District, Shenzhen 518083, China;

[3] BGI-Qingdao, No. 2877, Tuanjie Road, Sino-German Ecopark, Qingdao, Shandong 266555, China;

[4] Research Institute of Tropical Forestry, Chinese Academy of Forestry, Guangshanyi Rd, Tianhe District, Guangzhou 510000, China.

[#] These authors contributed equally to this work.

[*] To whom correspondence should be addressed: Qiang Gao (gaoqiang@bgi.com), Zhimin Gao (gaozhimin@icbr.ac.cn), and Zehui Jiang (jiangzehui@icbr.ac.cn)

Manuscript type: Data note

Abstract

Background: *Calamus simplicifolius* and *Daemonorops jenkinsiana* are two representative rattans, as the most significant material sources for the rattan industry. However, the lack of reference genome sequences is a major obstacle for basic and applied biology on rattan.

Findings: We rendered the two chromosome-level genome assemblies of *C. simplicifolius* and *D. jenkinsiana* using the Illumina, PacBio, and Hi-C sequencing data. A total of ~730 Gb and ~682 Gb of raw data covered the predicted genome length (~1.98 Gb of *C. simplicifolius* and ~1.61 Gb of *D. jenkinsiana*) to ~ 372× and ~ 426× read depth, respectively. The two *de novo* genome assemblies of ~1.94 Gb and ~1.58 Gb are generated with the scaffold N50 of ~160 Mb and ~119 Mb in *C. simplicifolius* and *D. jenkinsiana*, respectively. The *C. simplicifolius* and *D. jenkinsiana* genome were predicted to harbor 51,235 and 53,342 intact protein-coding gene models, respectively. BUSCO evaluation demonstrated that the genome completeness reached 96.4% and 91.3% in the *C. simplicifolius* and *D. jenkinsiana* genome, respectively. Genome evolution showed that four Arecaceae plants were located in a cluster and the divergence time between the two rattans was ~19.3 Mya. Additionally, we genome-widely identified 193 and 172 genes involved in the lignin biosynthesis pathway in *C. simplicifolius* and *D. jenkinsiana*, respectively.

Conclusions: We present the first *de novo* assemblies of the two rattan genomes (*C. simplicifolius* and *D. jenkinsiana*). These essential data will not only provide a fundamental resource of functional genomics particularly in promoting germplasm utilization for breeding improved rattan material property, but also will serve as two reference genomes for performing comparative studies between and among different species.

Keywords: Rattan, *Calamus simplicifolius*, *Daemonorops jenkinsiana*, whole genome sequencing, genome assembly, annotation

Background

Rattan is one of the world’s most important non-timber forest products and represents a major lineage of climbing palms occurring naturally in the Old World in a narrow sense [1]. A recent study indicates rattan is classified into 11 genera within the tribe of Calameae of the subfamily Calamoideae of the family Arecaceae in the world and consists of 631 species in a broad sense together with non-climbing palms in the same genera [2]. Among all genera, *Calamus* (NCBI Taxon ID:4711) and *Daemonorops* (NCBI Taxon ID:93268) are most diversified, accounting for ~65% and ~20% of rattan species [3], respectively. The two genera are also the most important material sources, producing more than 95% canes for the rattan industry. More than 5 million people depend economically on rattan and about 7 billion US dollars per year was made in the rattan industry, including domestic industry production and international trade of canes and their splitting, plaiting materials, baskets, seats and furniture [4]. With the increasing attention and the development of genetic and breeding techniques in rattan, the area of planting rattans is expected to gradually exceed that of natural rattans in recent years.

Calamus simplicifolius (NCBI Taxon ID:746888), is a deeply developed and indigenous rattan species in China (Fig. 1a), generally forming an open cluster of vigorous, unbranched stems with up to 50 m long and ~15 mm in diameter [5,6]. As a endemic rattan in Hainan Island, *C. simplicifolius* could produce the high-quality canes of medium diameter for binding and weaving in the rattan industry [5]. Additionally, *Daemonorops jenkinsiana* (NCBI Taxon ID:1510057), a representative species of high climbing evergreen rattan, is only one rattan species in *Daemonorops* genus (Fig. 1b) and naturally grows in the lowland rain forests below 1 km from Bangladesh, Bhutan, Cambodia, India, Laos, Myanmar, Nepal, Thailand, Vietnam to south-eastern China [2]. *D. jenkinsiana* could produce a dense cluster of vigorous stems that can be up to 50 m long and ~30 mm in diameter with internodes up to 40 cm long [6]. As the two most productive rattan species, *C. simplicifolius* and *D. jenkinsiana* are cultivated in areas with the latitude less than 23°30' N in China, i.e., Hainan island, Guangdong, Guangxi, Yunnan, Fujian and other areas of southern. Their established planting areas were estimated more than 1,000 ha [5].

C. simplicifolius and *D. jenkinsiana* have various applications and enormous developed potentials, and they are highly interesting mainly because of their canes with highly pliable and remarkably durable. Molecular breeding technology facilitate to meet growing requirements of the quantity and quality. However, the lack of genetic architecture underlying rattan's important traits severely hampered the comprehensive understanding of molecular biology in scientific research and actual production and in-depth performing comparative genome analyses between and among related species. Thus, we reported the two *de novo* genome assemblies of *C. simplicifolius* and *D. jenkinsiana* using the latest sequencing (Illumina and PacBio) and Hi-C mapping technology. With the available of the two chromosome-level reference genomes in rattan, many comparative genome analyses and other downstream applications will become feasible, such as the development of biomarkers, the analysis of functional genes, molecular design breeding and so on. Additionally, a high-quality genome assembly of two rattans facilitates genomic, transcriptomic, and metabolomics analyses of the material traits. As possible specific genes that could be of interest for improving material property, the lignin gene family members are identified in rattan. These studies lay the foundation for future research to understand and utilize the genes that determine rattan quality and its diversity within rattan germplasm.

Data description

DNA isolation, library construction, and sequencing

The young leaves at the vegetative growth stage of *C. simplicifolius* and *D. jenkinsiana* were collected in Spring 2015 from the Research Institute of Tropical Forestry of the Chinese Academy of Forestry in the city of Guangzhou, Guangdong Province, China (N: 23°11′29″, E: 113°22′40″, 87 M). The total DNA was isolated and extracted using DNeasy Plant Mini kit (Qiagen) based on the manufacturer's instruction. Genomic DNA was purified based on the protocol of the isolation of high-molecular-weight nuclear DNA. Multiple DNA libraries were constructed as described previously [7] and were sequenced on Illumina HiSeq 4000 and PacBio Sequel platform (Table 1). Briefly, we built three libraries with different insert sizes (270 bp, 500 bp and 800 bp) for paired-end (PE) sequencing and four libraries with different insert sizes (2 kb, 5 kb, 10 kb and 20 kb) for mate-pair (MP) sequencing

according to the standard Illumina protocol. We also constructed five PacBio Sequel libraries with 20 kb of insert size according to the standard PacBio protocol. After data cleaning and data preprocessing, we obtained 494.08 Gb of clean data (322.3 Gb PE reads, 93.4 Gb MP reads, and 78.38 Gb PacBio data), representing 252× coverage of the *C. simplicifolius* genome, and 426.17 Gb of clean data (244.58 Gb PE reads, 103.21 Gb MP reads, and 78.38 Gb PacBio data), representing 266 × coverage of the *D. jenkinsiana* genome, respectively.

Moreover, two Hi-C libraries were constructed for *C. simplicifolius* and *D. jenkinsiana*, respectively. We used Mbo I restriction enzyme to digest DNA after conformation fixing by formaldehyde and repaired 5′ overhang using biotinylated residue. Following ligating blunt-end fragments in situ, the isolated DNA was reverse-crosslinked, purified and filtered for biotin-containing fragments. Subsequently, DNA fragment end repair, adaptor ligation and PCR were performed in order. Then, the standard circularization step of BGISEQ-500 was carried out and sequencing was performed on BGISEQ500 with PE100 reads. Thus, we obtained ~6.7 Gb and ~13.1 Gb of valid data after ~148 Gb and ~154 Gb of raw data were evaluated and qualified using HiC-Pro (version 2.8.0_devel) [8] in *C. simplicifolius* and *D. jenkinsiana*, respectively (Table 1).

Genome survey

The understanding of genome characteristics for a given new species, i.e., genome size and heterozygosity, facilitates customizing a specific sequencing and assembling strategy. Thus, the genome size was estimated using four independence methods, i.e., a script of KmerSpectrumPlot.pl in ALLPATHS-LG (version r52488) [9], GCE (Genome Characteristics Estimation, released 20150107, ftp://ftp.genomics.org.cn/pub/gce), JELLYFISH (version 2.0) [10] and flow cytometry (only for *C. simplicifolius*) (Additional Tables S1-2 and Figs. S1-2). In our study, ~98 Gb and ~60 Gb of genome survey sequences were generated from short-insert size libraries in *C. simplicifolius* and *D. jenkinsiana*, respectively. In the data preprocessing, low-quality reads (the proportion of the base of Q<13 more than 40% in a given reads) were filtered using NGS QC Toolkit (version 2.3.3) [11] with default parameters. The combination (Additional Tables S1) showed the final predicted genome sizes

were ~1.98 Gb of *C. simplicifolius* and ~1.61 Gb of *D. jenkinsiana*, and the related heterozygosity was evaluated at 1.32%~1.52% and 1.19~1.31%, respectively. Thus, the genome survey suggested the two rattan genomes might be suitable for a hybrid sequencing strategy using Illumina and PacBio data.

Hybrid *de novo* genome assembly using Illumina, PacBio and Hi-C sequencing data

In the preprocessing of Illumina data, we filtered low-quality reads and adaptor sequences. Thus, ~416 Gb and ~348 Gb of clean data were generated in *C. simplicifolius* and *D. jenkinsiana*, respectively. For the PacBio data, we used MECAT (release 20170627) to correct errors [12] with the following parameters: -x 0 -i 0 -t 60 -r 0.8 -a 1000 -c 5 -l 2000. Thus, we obtained ~52 Gb and ~32 Gb of corrected PacBio data in *C. simplicifolius* and *D. jenkinsiana*, respectively. Subsequently, FALCON (version 0.3, https://github.com/PacificBiosciences/FALCON) was priority to perform assembling the initial contigs of the two rattans. As shown in Additional Table S3, the result showed two assemblies using different parameters were generated for the *C. simplicifolius* genome, e.g., 1.59 Gb of assembly size with 67.2 kb of contig N50 (~80% of the estimated genome size) and 1.53 Gb of assembly size with 66.7 kb of contig N50 (~77% of the estimated genome size). Additionally, 1.27 Gb of assembly size with 81.5kb of contig N50 (~79% of estimated genome size) was obtained for *D. jenkinsiana*. The performance of MECAT for the two rattans was still not very well. Thus, we assumed the incompleteness assembled scaffolds and low contig N50 may due to high heterozygosity (1.32%~1.52% for *C. simplicifolius* and 1.19~1.31% for *D. jenkinsiana*), high repeat sequences (54.15% for *C. simplicifolius* and 70% of *D. jenkinsiana*, see subsequent analysis for details), and inadequate sequencing depth, which contained ~26× and ~20× effective PacBio data after error correction, respectively. Therefore, according to the above investigations, we conducted hybrid *de novo* genome assembly of *C. simplicifolius* and *D. jenkinsiana* using Illumina and PacBio sequencing data. First, Platanus (version 1.2.4) [13], an *de novo* and high heterozygous genome assembler, was carried out to assemble the fragment PE reads into contigs by constructing De Bruijn Graphs with automatically optimized *k*-mer size. Second, the corrected PacBio reads and the assembled contigs

were thrown into DBG2OLC (release 20150611) [14] to construct scaffolds with the parameters: DBG2OLC Contigs contig.fa LD 0 K 17 KmerCovTh 4 MinOverlap 25 AdaptiveTh 0.007 RemoveChimera 1 f scaffold.fa. Hence, we obtained ~1.92 Gb and ~1.56 Gb of initial assembly sequences in *C. simplicifolius* and *D. jenkinsiana*, respectively. Third, a polish process before the SSPACE process referred to consensus analysis of DBG2OLC (Additional Table S4), which were contributed to enhance the quality of the genome assembly and reduce errors in the SSPACE process. Then, the assemblies were elongated by SSPACE (version 3.0) [15] using the MP reads and some gaps were filled using the Illumina and PacBio data by GapCloser (version 1.12) [16] and PBJelly (release 20150824) [17]. Thus, we obtained an assembly of 1.96 Gb, containing 5,116 scaffolds with a contig N50 length of 107 kb and a scaffold N50 of 803 kb in *C. simplicifolius*, and we also obtained another assembly of ~1.60 Gb in *D. jenkinsiana* and the N50 length was 108 kb and 784 kb for the contig and scaffold, respectively (Table 2).

Subsequently, the valid Hi-C data together with the above assembly were processed by 3D-DNA pipeline (version 170123) [18] to produce chromosome-level scaffolds. We obtained an explicit contact pattern which inferred a pretty accurate chromosome-level assembly. As shown in Fig. 2, the contact maps were visualized by Juicerbox (version 1.5.2) [19]. The length of the longest 12 chromosome-level scaffolds for the *C. simplicifolius* assembly and that of 13 for the *D. jenkinsiana* assembly were presented in Additional Table S5. The total length of the pseudo-chromosomes accounted for 92.08% and 92.01% of *C. simplicifolius* and *D. jenkinsiana*, which with scaffold N50 of 169 Mb and 119 Mb, respectively.

Genome evaluation

Three independent methods were used to evaluate the accuracy and completeness of the *C. simplicifolius* and *D. jenkinsiana* assemblies. First, two genome features were summarized, i.e., the percentage of ambiguous bases (Ns) and GC content. The result showed a low percentage of Ns (~ 0.6% of *C. simplicifolius* and ~ 0.7% of *D. jenkinsiana*) was observed and overall GC contents (41.07% of *C. simplicifolius* and 41.78% of *D. jenkinsiana*) were similar to related transcriptomic data (41.68% of *C. simplicifolius* and 41.89% of *D. jenkinsiana*). Then, the unigenes assembled by RNA-

Seq data were aligned to the assembly using BLAT (version 1.0) [20] with default parameters. The alignment results showed more than 90% sequences in one scaffold reach to a high proportion (92.89% of *C. simplicifolius* and 81.81% of *D. jenkinsiana*) (Additional Table S6). Last, the completeness of the two rattans assemblies was evaluated using BUSCO (version 3.0) [21], which quantitatively assesses genome completeness using evolutionarily informed expectations of gene content from near-universal single-copy orthologs. BUSCO results showed 96.4% of conserved BUSCO proteins (embryophyta_odb9) were detected in the *C. simplicifolius* assembly, including 3.8% of the fragment BUSCO proteins. Additionally, 87.3% and 4.0% of the conserved BUSCO proteins were identified as the complete and fragment in *D. jenkinsiana*, respectively (Additional Table S7).

Repeat annotation

Before the prediction of protein-coding gene models, transposable elements (TEs) and tandem repeats were identified in the *C. simplicifolius* and *D. jenkinsiana* assembly, respectively. We adopted two independent approaches to predict repetitive elements, i.e., homology-based annotation and *de novo* method. In the homology-based annotation, TEs were identified using RepeatMasker (v4.0.5) and RepeatProteinMasker (v4.0.5) [22] via searching against Repbase library (release 201712) [23]. In *de novo* annotation, a *de novo* repeat library was constructed using RepeatModeler (v1.0.8, http://www.repeatmasker.org/RepeatModeler/) and LTR_FINDER [24] after eliminating contaminant and multi-copy genes. Then, RepeatMasker were performed to categorize the genome sequence against the *de novo* repeat library. Additionally, tandem repeat sequences were identified by Tandem Repeat Finder (version 4.09) [25] with the following parameters: “Match = 2, Mismatch = 7, Delta = 7, PM = 80, PI = 10, Minscore = 50 and MaxPerid = 2000”. Thus, the common result showed that long terminal repeat (LTR) was the most abundant repeats as well as SINE and LINE, two non-LTR retrotransposons, were lowest proportions in the two rattan assemblies (Additional Table S8). TEs were accounted for 54.15% and 70% of the *C. simplicifolius* and of *D. jenkinsiana* assembly, respectively, and the sequence divergence of TEs indicated that the *de novo* predicted repeats were more recently-active than the Repbase predicted repeats (Fig.3).

RNA sample collection, library construction, and transcriptome assembly

The four samples of the distal cirrus with three developmental stages were collected in *C. simplicifolius* and *D. jenkinsiana*, respectively. Each sample has three biological replicates (Additional Table S9). As one part of the rattan genome project, the location of RNA sampling was consistent with that of DNA sampling. Based on the manufacturer's instructions, RNA was isolated using TRIzol Reagent Solution (Invitrogen, Carlsbad, CA, USA) and the purity and concentration were determined with a NanoDrop 2000 spectrophotometer. Reverse transcription was conducted with a Reverse Transcription System (Promega, USA). The extracted RNA was treated with RNase-free DNase I for 30 min at 37 ºC to remove the residual DNA as described previously [26] and then the pooled libraries were sequenced using the BGISEQ500 platform with 100 short PE reads. In the preprocessing of the transcriptomic data, adaptor sequences and low-quality reads were filtered using SOAPnuke (version 1.5.6, https://github.com/BGI-flexlab/SOAPnuke) with the following parameters: "-n 0.001 -l 20 -q 0.4 -Q 2". The clean reads of all samples were together assembled using Trinity (version 2.0.6)[27] with the following parameters: (1) group_pairs_distance 500, (2) min_contig_length 200, (3) min_kmer_cov 2, (4) min_glue 2, (5) bfly_opts -V 5, (6) edge-thr=0.1, (7) stderr, and (8) SS_lib_type RF. Then, the outputs of Trinity were clustered to generate a single set of non-redundant references using TGI Clustering Tool (version v2.0.6) [28] with the following parameters: (1) a minimum of 95% identity between the contigs, (2) a minimum of 35 overlapping bases, (3) a minimum of 35 scores, and (4) a maximum of 20 unmatched overhanging bases at the sequence ends. Ultimately, the assembled transcripts were divided into the two classes based on sequence similarity: clusters (prefixed with 'CL') and singletons (prefixed with 'unigene'). In each cluster, the sequence similarity regions between the transcripts were more than 70% and the transcripts were spliced isoforms from a gene or a paralogous gene. Additionally, all unigenes were used in subsequent analyses.

Gene modeling and prediction

We performed a considerate prediction of intact protein-coding gene models using three independent approaches [7], i.e., *de novo* prediction, homology-based method, and RNA-Seq approach. The repeat masked assembly was firstly annotated by AUGUSTUS (version 3.3) with default parameters [29], which was a *de novo* predictor based on the self-trained model. Via optimizing training data and multiple trainings, the results showed 85,246 and 87,613 gene models were predicted in *C. simplicifolius* and *D. jenkinsiana*, respectively. In the homology-based prediction, we used seven species as reference datasets, i.e., *Elaeis guineensis*, *Phoenix dactylifera*, *Brachypodium distachyon*, *Oryza sativa*, *Setaria italic*, *Sorghum bicolor*, and *Zea mays* (see Availability of supporting data for individual genome version). Their protein sequences were downloaded for ENSEMBL database [30] and were aligned to the *C. simplicifolius* and *D. jenkinsiana* assembly using TBLASTN (version 2.2.26) [31] with an E-value cutoff of 1e-5, respectively. Then, the splicing patterns were generated by GeneWise (version 2.0) [32]. In the RNA-Seq analysis, HISAT2 (version 2.0.2) [33] was used to identify exon-intron splicing junctions and refine the alignment of the RNA-Seq reads to the genome. Then, we used Cufflinks (version 2.2.1) [34] to define 56,024 and 58,134 protein-coding gene models in *C. simplicifolius* and *D. jenkinsiana*, respectively (Additional Table S10).

Lastly, we integrated the evidences from the three above independent predictions using MARKER (version 2) [35]. The final prediction results showed 51,235 and 53,342 intact protein-coding gene models were predicted as a consensus gene set in *C. simplicifolius* and *D. jenkinsiana*, respectively.

Annotation evaluation and gene function prediction

We evaluated the predicted annotations using two independent methods, i.e., gene function evaluation and completeness evaluation by BUSCO. In the gene function evaluation, we assessed the agreement of predicted annotations with aligned protein evidences and/or of homologous proteins to closely related species by manual annotations. The result of aligned against five authoritative protein databases (Additional Table S11) indicated 5.34% and 2.89% of the predicted gene models were identified as unannotated genes in *C. simplicifolius* and *D. jenkinsiana*, respectively. These protein databases included non-redundant NCBI protein database (release 20180313) [36], SWISS-PROT (release 201801) [37], GO (release 20131030) [38], KEGG (dataset v81) [39], and InterPro (dataset

v.53) [40]. Additionally, BUSCO evaluation showed 88.7% and 91.3% of conserved BUSCO proteins (embryophyta_odb9) were present in *C. simplicifolius* and *D. jenkinsiana*, respectively. Among the conserved BUSCO proteins, 76.2% and 81.2% were complete. Furthermore, the four types of non-coding RNA genes, i.e., tRNA, rRNA, miRNA and snRNA, were also predicted (Additional Table S12).

Gene family construction and rattan species-specific gene families

In our study, we performed a pairwise sequence comparison to genome-widely predict orthologous genes. The method is faster and generally deals with larger amounts of data. As a popular BLAST-based approach, OrthoMCL (version 2.0.9) [41] was used to identified orthologous genes in *C. simplicifolius*, *D. jenkinsiana* with an E-value cutoff of 1e-5 and a percent match cut-off of 80 (i.e., query and match had to overlap on more than 80% of the query and match sequence length). Markov Chain Clustering was also used with a default inflation parameter in an all-to-all BLASTP analysis of entries for other 8 plants, i.e., *Amborella trichopoda*, *E. guineensis*, *A. thaliana*, *B. distachyon*, *O. sativa*, *S. polyrhiza*, *P. dactylifera* and *S. bicolor* (see Availability of supporting data for individual genome version). Among 30,936 gene families identified in entire 10 species, 44,700 and 44,537 orthologous genes were detected in the *C. simplicifolius* and *D. jenkinsiana* genome, respectively. Approximately 6,132 (19.8%) gene families confined entire 10 species as well as 2,366 and 2,707 specific gene families were detected in *C. simplicifolius* and *D. jenkinsiana*, respectively (Fig 4b). Additionally, the result showed that 637 gene families were specific to the rattans. The rattan species-specific gene families were enriched in gene ontology (GO) categories related to component membrane and transcription factor activity (Additional Table S13), and in KEGG pathways related to plant-pathogen interaction and plant hormone signal transduction (Additional Table S14).

Phylogenetic analysis and Divergence time

We obtained 962 single-copy orthologous genes derived from entire gene families, which are conserved among species to facilitate understanding the evolutionary relationship of rattan with other species. First, multiple alignments of protein sequences were conducted by MUSCLE (version 3.8.31)

[42], and then CDS alignment was constructed based on the protein alignments. Subsequent, all aligned CDS sequences were concatenated to generate a super gene for each species using an in-house Perl script. Thus, we extracted the nucleotides at position 2 (phase1) of each codon to construct the phylogenetic tree using RAxML (version 8.2.3) [43] with the model "GTRGAMMA". The results showed that four Arecaceae plants were located in a cluster, which comprised of two independent sister branches that contain *C. simplicifolius*, *D. jenkinsiana* and the *E. guineensis*, *P. dactylifera* respectively. (Fig. 4a).

Moreover, we used the MCMCTree program of PAML (version 4.5) [44] to estimate the divergence time among *C. simplicifolius*, *D. jenkinsiana* and the other 8 plants with the following parameters: "-nsample 200000 -burnin 40000". The calibration times were derived published timings for the divergence of the reference species [45]. The result indicated that the divergence time between two rattans was ~19.3 Mya and for other two Arecaceae genes, *P. dactylifera* was separated from *E. guineensis* at ~40.8 Mya (Fig. 4c).

Genome-wide identification of gene families involved in the lignin biosynthesis pathway

Lignin is a class of complex aromatic heteropolymers of monolignols that encrusts and interacts with the cellulose/hemicellulose matrix of the secondary cell wall. The aromatic lignin polymers are commonly composed of three monolignols, i.e., *p*-hydroxyphenyl (H), vanillin (G), and syringaldehyde (S)[46]. Thus, we performed genome-wide identification of 13 gene families involved in the lignin biosynthesis pathway of rattan using the 8 genomes, i.e., *A. thaliana*, *B. distachyon, O. sativa, S. bicolor, Phyllostachys* edulis, *Populus trichocarpa*, *D. jenkinsiana*, and *C. simplicifolius.* Most genome sequences (*A. thaliana*, *B. distachyon*, *O. sativa*, *S. bicolor*, and *Populus trichocarpa*) were downloaded from the ENSEMBL database [47]. The genome sequences of *Ph. edulis* was downloaded from the Bamboo Genome Database [48]. According to vast literature-based investigations, one-hundred forty genes from the lignin biosynthetic pathway was collected from experimentally validation of previous studies (Additional Table S15), and then, these known genes were used as the query sequences for further identification. A BLAST search and domain analysis as described previously [54] was used in the genome-wide identification. Briefly, we performed standard

protein BLAST searches (version 2.2.26) against all genome sequences including two rattans using the coding sequence of the known genes with the following cut-off values: E-value <1e-10; identity >40%; and coverage rate >95% query sequence. The filtered sequences were subsequently analyzed by hmmsearch (version 3.1b2) using the Pfam-A.hmm database (released 2017/03/31). Consequently, unclear sequences with incomplete domains were discarded by manual correction. The result showed the expansion of most families was detected in two rattans (Table 3). Each gene contained multiple copies with an average of ~15 and ~13 copies per family in *C. simplicifolius* and *D. jenkinsiana*, respectively. The total size of the gene families in the lignin biosynthesis pathway contained 193 and 172 gene copies in *C. simplicifolius* and *D. jenkinsiana*, respectively. Peroxidase (POD), as the most copy number, was detected both in the two rattans. As the least copy number, phenylalanine ammonia-lyase (PAL) was identified in *C. simplicifolius* and coumarate 3-hdroxylase (C3H) and cinnamate 4-hydroxylase (C4H) were detected in *D. jenkinsiana*. The expansion of the lignin biosynthesis genes in rattan could be due to the occurrence of whole genome duplication (WGD) event, since WGD could provide more gene copies, which facilitated evolving the genes with new functions [49].

Conclusion

We reported the two chromosome-level reference genome sequences in rattan (*C. simplicifolius* and *D. jenkinsiana*) using the multiple types of sequencing data and assembly technologies. The availability of the *C. simplicifolius* and *D. jenkinsiana* genome could facilitate *de novo* genome assembling and resequencing of other species in rattan, and server as essential resources to identify regions providing a suitable resolution in an evolutionary landscape by performing comparative studies between and among different species. The two high-quality rattan genomes become easier to identify the critical genes involved in the lignin biosynthesis pathways that have potential developmental importance. Therefore, these data pave the way for extra-genomic studies in rattan and related plants.

Availability of supporting data

The datasets supporting the results of this article are available in the *Giga*DB repository [50]. All raw genomic sequence reads from BIGSEQ500, Illumina and PacBio platform, and transcriptome reads derived from multiple tissues have been uploaded and deposited in the European Nucleotide Sequence Archive (EMBL-EBI) with the project accession No. PRJEB24031 and PRJEB24829 for *C. simplicifolius* and *D. jenkinsiana*, respectively. Other analytical data in this study included *A. trichopoda* (version 1.0) downloaded from Amborella Genome Database (amborella.huck.psu.edu) and *E. guineensis* (version GCF_000442705.1) downloaded from NCBI database. Except for the above two species, other genomes were downloaded from ENSEMBL database, including *E. guineensis* (version GCF_000442705.1), *Ph. dactylifera* (version 1.0), *B. distachyon* (version 3.1), *O. sativa* (version R498), *S. italic* (version 9.0), *S. bicolor* (version 3.1), *Z. mays* (version B73_RefGen_V4), *Ph. edulis* (version 2), *P. trichocarpa* (JGI2.0.31), *P. dactylifera* (version 1) and *A. thaliana* (version: TAIR10).

Abbreviations

NCBI: National Center for Biotechnology Information; SRA: Sequence Read Archive (SRA); RNA-Seq: RNA-sequencing; BUSCO: Benchmarking Universal Single-Copy Ortholog; GO: Gene Ontology; TE: transposable element; GABR: Genome Atlas of Bamboo and Rattan; PE: paired-end; MP: mate-pair; LTR: long terminal repeat; GO: genome ontology; KEGG: Kyoto Encyclopedia of Genes and Genomes.

Additional files

Additional Table S1: Evaluation of genome size of *C. simplicifolius* and *D. jenkinsiana*

Additional Table S2: 17-mer frequency method estimated genome size of *C. simplicifolius* and *D. jenkinsiana*

Additional Table S3: Statistics of the assemblies using different assembling strategies

Additional Table S4: BUSCO evaluation of the polish process

Additional Table S5: The chromosome-level length of Hi-C assembly in *C. simplicifolius* and *D. jenkinsiana*

Additional Table S6: Statistics of the quality assessment of the *C. simplicifolius* and *D. jenkinsiana* genomes

Additional Table S7: BUSCO evaluation of the *C. simplicifolius* and *D. jenkinsiana* genomes

Additional Table S8: Statistics of the predicted repetitive sequences in the *C. simplicifolius* and *D. jenkinsiana* genomes

Additional Table S9: Statistics of RNA libraries in transcriptome assembly

Additional Table S10: Statistics of the predicted protein-coding genes in the *C. simplicifolius* and *D. jenkinsiana* genomes

Additional Table S11: Statistics of functional annotations of the *C. simplicifolius* and *D. jenkinsiana* genomes

Additional Table S12: Statistics of the predicted non-coding RNAs in the *C. simplicifolius* and *D. jenkinsiana* genomes

Additional Table S13: GO analysis of rattan specific-species gene families

Additional Table S14: KEGG analysis of rattan specific-species gene families

Additional Table S15: Totally 140 genes of lignin biosynthetic pathway were experimentally validated from the previous studies.

Additional Figure S1: Evaluation of genome size of *C. simplicifolius* and *D. jenkinsiana* genomes by K-mer

Additional Figure S2: Evaluation of genome size of *C. simplicifolius* by flow cytometry

Competing interests

The authors have declared that there are financial and non-financial competing interests in this study.

Funding

This work was supported by the Sub-Project of the National Science and Technology Support Plan of the Twelfth Five-Year Plan in China (No. 2015BAD04B03 and 2015BAD04B01) and Fundamental Research Funds for the International Center for Bamboo and Rattan (No. 1632017018).

Author contributions

HSZ and RSL collected the samples; JLW, HYS, SNW, HX, KBY, XRX, XMS, and JJS constructed libraries; HSZ, SBW, CHC, LFC, AQF, CZ, and QG performed the genome assembly. SJH, KH, CCS, and GYF performed the Hi-C analysis. SBW and LFC performed the genome annotation; HSZ, SBW, LFC, and XL analyzed the genome data. HSZ and SBW wrote the manuscript; HSZ, SBW, XL, ZMG and ZHJ reviewed the manuscript. All above authors have read and approved the final manuscript.

Acknowledgements

As a part of Genome Atlas of Bamboo and Rattan (GABR), we wish to acknowledge the GABR Consortium members, partners, advisors, and supporters who have helped this project run smoothly.

Reference

1. Jiang Z. Bamboo and Rattan in the World. Beijing: China Forestry Publishing House. 2007.

2. International Network for Bamboo and Rattan. World Checklist of Bamboo and Rattans. Beijing: International Network of Bamboo and Rattan; 2017.

3. Larsen K. Genera Palmarum. A classification of palms based on the work of Harold E. Moore Jr. Nordic Journal of Botany. 1989;9:62–2.

4. Kumar HNK, Preethi SD, Chauhan JB. Studies on the *in vitro* propagation of *Calamus travancoricus*. Asian Journal of Plant Science and Research. 2012;:1–7.

5. Li R-S, Yin G-T, Yang J-C, Zou W-T. Rattan sector in Hainan Island, China: a case study. Journal of Forestry Research. 2007;18:153–6.

6. Internet EPOT. eFloras Published on the Internet. Missouri Botanical Garden, St. Louis, MO, & Harvard University Herbaria, Cambridge, MA. 2008. http://www.eoras.org. Accessed 20 May 2017.

7. Peng Z, Lu Y, Li L, Zhao Q, Feng Q, Gao Z, et al. The draft genome of the fast-growing non-timber forest species moso bamboo (*Phyllostachys heterocycla*). Nature Genetics. 2013;45:456–61.

8. Servant N, Varoquaux N, Lajoie BR, Viara E, Chen C-J, Vert J-P, et al. HiC-Pro: an optimized and flexible pipeline for Hi-C data processing. Genome Biology. 2015;16:259.

9. Maccallum I, Przybylski D, Gnerre S, Burton J, Shlyakhter I, Gnirke A, et al. ALLPATHS 2: small genomes assembled accurately and with high continuity from short paired reads. Genome Biology. 2009;10:R103.

10. Marçais G, Kingsford C. A fast, lock-free approach for efficient parallel counting of occurrences

of k-mers. Bioinformatics. 2011;27:764–70.

11. Patel RK, Jain M. NGS QC Toolkit: A Toolkit for Quality Control of Next Generation Sequencing Data. Liu Z, editor. PloS one. 2012;7:e30619.

12. Xiao C-L, Chen Y, Xie S-Q, Chen K-N, Wang Y, Han Y, et al. MECAT: fast mapping, error correction, and de novo assembly for single-molecule sequencing reads. Nature Methods. 2017;14:1072–4.

13. Kajitani R, Toshimoto K, Noguchi H, Toyoda A, Ogura Y, Okuno M, et al. Efficient *de novo* assembly of highly heterozygous genomes from whole-genome shotgun short reads. Genome research. 2014;24:1384–95.

14. Ye C, Hill CM, Wu S, Ruan J, Ma ZS. DBG2OLC: Efficient Assembly of Large Genomes Using Long Erroneous Reads of the Third Generation Sequencing Technologies. Scientific Reports. 2016;6:31900.

15. Hunt M, Newbold C, Berriman M, Otto TD. A comprehensive evaluation of assembly scaffolding tools. Genome Biology. 2014;15:R42.

16. Luo R, Liu B, Xie Y, Li Z, Huang W, Yuan J, et al. Erratum: SOAPdenovo2: an empirically improved memory-efficient short-read de novo assembler. GigaScience. 2015;4:30.

17. English AC, Richards S, Han Y, Wang M, Vee V, Qu J, et al. Mind the gap: upgrading genomes with Pacific Biosciences RS long-read sequencing technology. Liu Z, editor. PloS one. 2012;7:e47768.

18. Dudchenko O, Batra SS, Omer AD, Nyquist SK, Hoeger M, Durand NC, et al. *De novo* assembly of the *Aedes aegypti* genome using Hi-C yields chromosome-length scaffolds. Science. 2017;356:92–5.

19. Zhao H, Dong L, Sun H, Li L, Lou Y, Wang L, et al. Comprehensive analysis of multi-tissue transcriptome data and the genome-wide investigation of GRAS family in *Phyllostachys edulis*. Scientific Reports. 2016;6:27640.

20. Kent WJ. BLAT--the BLAST-like alignment tool. Genome research. 2002;12:656–64.

21. Simão FA, Waterhouse RM, Ioannidis P, Kriventseva EV, Zdobnov EM. BUSCO: assessing genome assembly and annotation completeness with single-copy orthologs. Bioinformatics. 2015;31:3210–2.

22. Tarailo-Graovac M, Chen N. Using RepeatMasker to identify repetitive elements in genomic sequences. Curr Protoc Bioinformatics. Hoboken, NJ, USA: John Wiley & Sons, Inc; 2009;Chapter 4:Unit4.10–4.10.14.

23. Bao W, Kojima KK, Kohany O. Repbase Update, a database of repetitive elements in eukaryotic genomes. Mobile DNA. 2015;6:11.

24. Xu Z, Wang H. LTR_FINDER: an efficient tool for the prediction of full-length LTR retrotransposons. Nucleic Acids Research. 2007;35:W265–8.

25. Benson G. Tandem repeats finder: a program to analyze DNA sequences. Nucleic Acids Research. 1999;27:573–80.

26. Zhao H, Sun H, Li L, Lou Y, Li R, Qi L, et al. Transcriptome-based investigation of cirrus development and identifying microsatellite markers in rattan (*Daemonorops jenkinsiana*). Scientific

Reports. 2017;7:46107.

27. Haas BJ, Papanicolaou A, Yassour M, Grabherr M, Blood PD, Bowden J, et al. *De novo* transcript sequence reconstruction from RNA-seq using the Trinity platform for reference generation and analysis. Nature Protocol. 2013;8:1494–512.

28. Pertea G, Huang X, Liang F, Antonescu V, Sultana R, Karamycheva S, et al. TIGR Gene Indices clustering tools (TGICL): a software system for fast clustering of large EST datasets. Bioinformatics. 2003;19:651–2.

29. Stanke M, Morgenstern B. AUGUSTUS: a web server for gene prediction in eukaryotes that allows user-defined constraints. Nucleic Acids Research. 2005;33:W465–7.

30. Zerbino DR, Johnson N, Juetteman T, Sheppard D, Wilder SP, Lavidas I, et al. Ensembl regulation resources. Database (Oxford). 2016;2016:bav119.

31. Mount DW. Using the Basic Local Alignment Search Tool (BLAST). CSH Protocol. 2007;2007:pdb.top17.

32. Birney E, Durbin R. Using GeneWise in the Drosophila annotation experiment. Genome research. 2000;10:547–8.

33. Kim D, Langmead B, Salzberg SL. HISAT: a fast spliced aligner with low memory requirements. Nature Methods. 2015;12:357–60.

34. Ghosh S, Chan C-KK. Analysis of RNA-Seq Data Using TopHat and Cufflinks. Methods in Molecular Biology. 2016;1374:339–61.

35. Holt C, Yandell M. MAKER2: an annotation pipeline and genome-database management tool for second-generation genome projects. BMC Bioinformatics. 2011;12:491.

36. O'Leary NA, Wright MW, Brister JR, Ciufo S, Haddad D, McVeigh R, et al. Reference sequence (RefSeq) database at NCBI: current status, taxonomic expansion, and functional annotation. Nucleic Acids Research. 2016;44:D733–45.

37. Boutet E, Lieberherr D, Tognolli M, Schneider M, Bansal P, Bridge AJ, et al. UniProtKB/Swiss-Prot, the Manually Annotated Section of the UniProt KnowledgeBase: How to Use the Entry View. Methods in Molecular. Biology. 2016;1374:23–54.

38. Gene Ontology Consortium. The Gene Ontology (GO) database and informatics resource. Nucleic Acids Research. 2004;32:258D–261.

39. Kanehisa M, Furumichi M, Tanabe M, Sato Y, Morishima K. KEGG: new perspectives on genomes, pathways, diseases and drugs. Nucleic Acids Research. 2017;45:D353–61.

40. Finn RD, Attwood TK, Babbitt PC, Bateman A, Bork P, Bridge AJ, et al. InterPro in 2017-beyond protein family and domain annotations. Nucleic Acids Research. 2017;45:D190–9.

41. Chen F, Mackey AJ, Stoeckert CJ, Roos DS. OrthoMCL-DB: querying a comprehensive multi-species collection of ortholog groups. Nucleic Acids Research. 2006;34:D363–8.

42. Edgar RC. MUSCLE: multiple sequence alignment with high accuracy and high throughput. Nucleic Acids Research. 2004;32:1792–7.

43. Stamatakis A. RAxML version 8: a tool for phylogenetic analysis and post-analysis of large phylogenies. Bioinformatics. 2014;30:1312–3.

44. Yang Z. PAML 4: phylogenetic analysis by maximum likelihood. Molecular Biology and Evolution. 2007;24:1586–91.

45. Kumar S, Stecher G, Suleski M, Hedges SB. TimeTree: A Resource for Timelines, Timetrees, and Divergence Times. Molecular Biology and Evolution. 2017;34:1812–9.

46. Martone PT, Estevez JM, Lu F, Ruel K, Denny MW, Somerville C, et al. Discovery of lignin in seaweed reveals convergent evolution of cell-wall architecture. Current Biology. 2009;19:169–75.

47. Kersey PJ, Allen JE, Allot A, Barba M, Boddu S, Bolt BJ, et al. Ensembl Genomes 2018: an integrated omics infrastructure for non-vertebrate species. Nucleic Acids Research. 2018;46:D802–8.

48. Zhao H, Peng Z, Fei B, Li L, Hu T, Gao Z, et al. BambooGDB: a bamboo genome database with functional annotation and an analysis platform. Database (Oxford). 2014;2014:bau006–6.

49. Taylor JS, Raes J. Duplication and divergence: the evolution of new genes and old ideas. Annual Review Genetics. 2004;38:615–43.

50. Sneddon TP, Li P, Edmunds SC. GigaDB: announcing the GigaScience database. GigaScience. 2012;1:11.

Figure legends

Figure 1. Morphological characteristics of *C. simplicifolius* and *D. jenkinsiana*

The series pictures of A and B displayed different morphological characteristics of *C. simplicifolius* and *D. jenkinsiana*, respectively. (a1) a young *C. simplicifolius*; (a2) a middle-aged *C. simplicifolius*; (a3) a climbing *C. simplicifolius*; (a4) a mature *C. simplicifolius*; (a5) a nursery of *C. simplicifolius*; (b1) a young *D. jenkinsiana*; (b2) a young forest of *D. jenkinsiana*; (b3) a nursery of *D. jenkinsiana*; (b4) leaves of *D. jenkinsiana*; (b5) inflorescences of *D. jenkinsiana*; (b6) young fruits of *D. jenkinsiana*. All the pictures were taken by Prof. Rongsheng Li.

Figure2 The Hi-C contact map of the *C. simplicifolius* (a) and *D. jenkinsiana* genome (b).

(c) and (d) show the Hi-C links on hic_scaffold_4 of *C. simplicifolius* and hic_scaffold_10 of *D. jenkinsiana* before (top) and after (bottom) conflict resolving. (e) and (f) show the distribution of Hi-C links decay along the genomic distance.

Figure 3. The distribution of the sequence divergence rate of different TE types in the *C. simplicifolius* (a) and *D. jenkinsiana* (b) genome

Figure 4. The phylogenetic tree, orthologous gene families and divergence times among *C. simplicifolius*, *D. jenkinsiana*, and other 8 plants

(a). The phylogenetic tree was constructed by RAxML using all single-copy genes in the 10 species and the divergence time was estimated using the MCMCTree programmer in the PAML software package. (b). Clusters of the orthologous and paralogous gene families in *C. simplicifolius*, *D. jenkinsiana* and other 8 full-sequenced plants using OrthoMCL. (c). The number on the nodes are divergence times and the red nodes indicate the calibration times.

Table 1: Statistics of the clean data of the *C. simplicifolius* and *D. jenkinsiana* genomes

Sequencing Platform	Insert Size	*C. simplicifolius*			*D. jenkinsiana*		
		Reads Length (bp)	Total Data (Gb)	Sequence Depth (X)*	Reads Length (bp)	Total Data (Gb)	Sequence Depth (X)*
Illumina	270 bp	150	160.9	82.09	150	98.21	61.38
	500 bp	125	60.2	30.71	125	56.9	35.56
	800 bp	125	101.2	51.63	125	89.47	55.91
	2 Kb	49	22.8	11.63	49	33.08	20.67
	5 Kb	49	16.4	8.37	49	22.1	13.81
	10 Kb	49	26.8	13.67	49	32.63	20.39
	20 Kb	49	27.4	13.98	49	15.4	9.6
PacBio	20 Kb	9,079**	78.38	39.99	9,131**	78.38	48.75
Hi-C	N.A.	100	6.7	3.42	100	13.1	8.19
Total			500.78	255.5		439.27	274.26

*Read length of PacBio means an average length

**Sequencing depth was calculated based on 1.98 Gb of the *C. simplicifolius* genome and 1.61 Gb of the *D. jenkinsiana* genome

Table 2: Metrics of the final assembly of the *C. simplicifolius* and *D. jenkinsiana* genome

**Items**		***C. simplicifolius***		***D. jenkinsiana***	
		**hybrid assembly***	**Hi-C assembly**	**hybrid assembly***	**Hi-C assembly**
**Contig**	Number	29,973	29,973	27,631	27,631
	Size (bp)	1,923,260,127	1,923,260,127	1,570,849,893	1,570,849,893
	N50 (bp)	99,304	99,304	89,562	89,562
	N90 (bp)	28,872	28,872	25,720	25,720
**Scaffold**	Number	29,775	5,283	27,146	5,126
	Size (bp)	1,923,287,712	1,935,533,712	1,570,878,714	1,581,888,714
	N50 (bp)	99,590	160,072,219	89,705	119,093,744
	N90 (bp)	28,922	93,668,489	25,828	61,330,142
**Total number**	>3 kb	29,767	5,275	27,137	5,117
	>5 kb	29,727	5,235	27,081	5,061
**The longest sequence (bp)**		877,470	219,145,773	1,422,351	162,635,149
**The shortest sequence (bp)**		1,286	1,286	719	719
**Ns ratio (%)**		0.0	0.6	0.0	0.7
**GC ratio (%)**		41.07	41.07	41.78	41.78

* hybrid assembly means *de novo* assembly using Illumina and PacBio data in our study

Table 3. The family number in lignin biosynthesis pathway

Family	*C. simplicifolius*	*D. jenkinsiana*	*A. thaliana*	*B. distachyon*	*O. sativa*	*Ph. edulis*	*P. trichocarpa*	*S. bicolor*	Total
4CL: 4-coumarate CoA ligase	9	13	12	13	12	15	13	16	90
C3H: Coumarate 3-hdroxylase	3	2	3	1	1	3	3	2	15
C4H: Cinnamate 4-hydroxylase	3	2	1	2	3	6	2	2	19
CAD: Cinnamyl alcohol dehydrogenase	29	22	9	7	10	14	17	11	102
CCoAOMT: Caffeoyl-CoA 3-O-methyltransferase	16	5	4	7	6	9	5	5	52
CCR: Cinnamoyl-CoA reductase	6	6	3	9	12	17	10	11	64
COMT: Caffeic acid 3-O-methyltransferase	13	16	11	4	6	4	11	5	59
F5H: Ferulate 5-hydroxylase	7	6	1	4	5	16	17	11	50
HCT: hydroxycinnamoyl-CoA	5	4	3	12	6	16	7	13	59
LAC: Laccase	29	29	16	22	20	41	47	21	178
PAL: Phenylalanine ammonia-lyase	2	7	4	9	8	12	5	10	52
CHS: Chalcone synthase	31	17	4	7	17	12	13	27	115
POD: Peroxidase	40	43	45	44	37	77	56	42	328
Total	193	172	116	141	143	242	206	176	--

Figure 1 Click here to download Figure Fig1.Morphlogical features.pdf

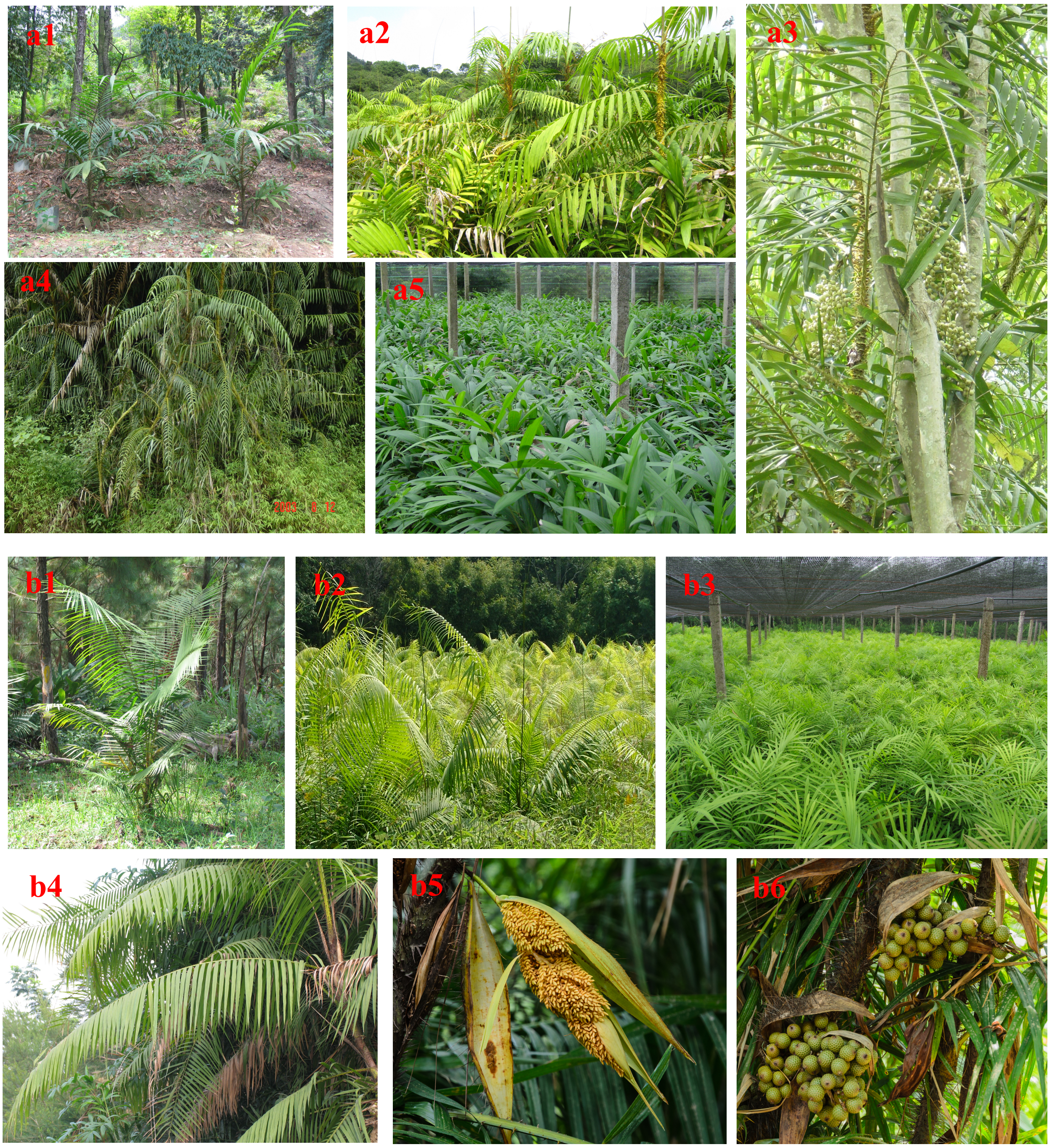

Figure 2

Click here to download Figure Fig2.Hic.pdf

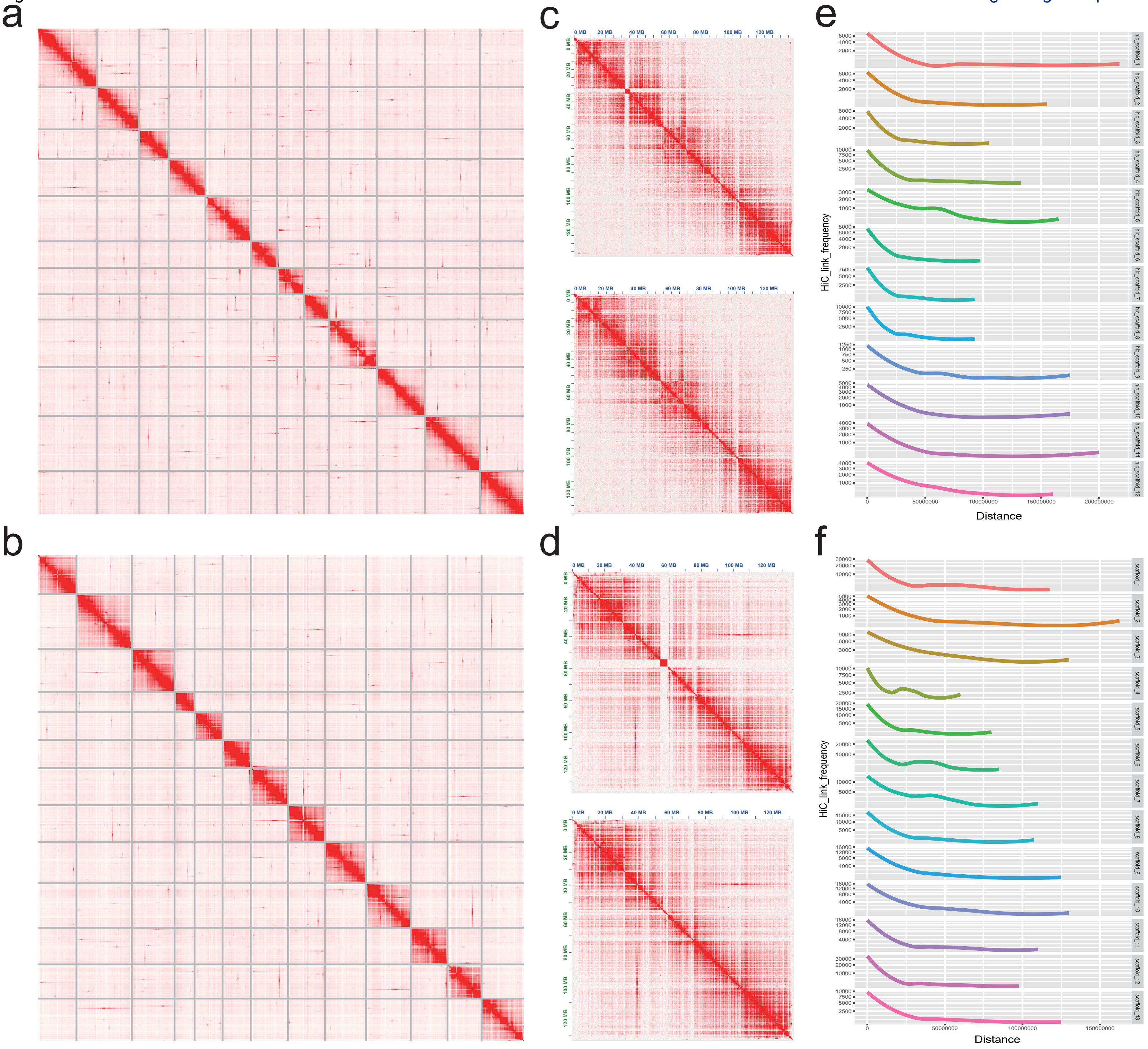

Figure 3

Click here to download Figure Fig3.Divergence rate distribution of different types of TE.pdf

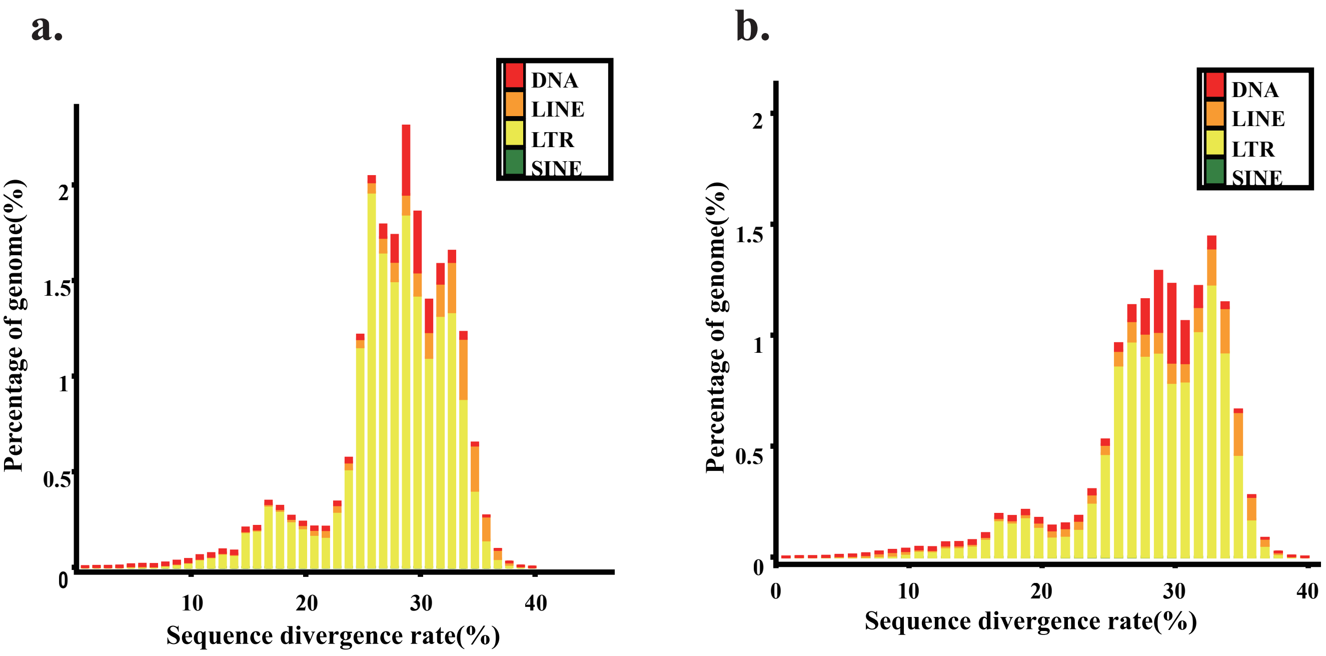

Figure 4

Click here to download Figure Fig4.phylogenetic and divergence time add P.dactylifera(1).pdf

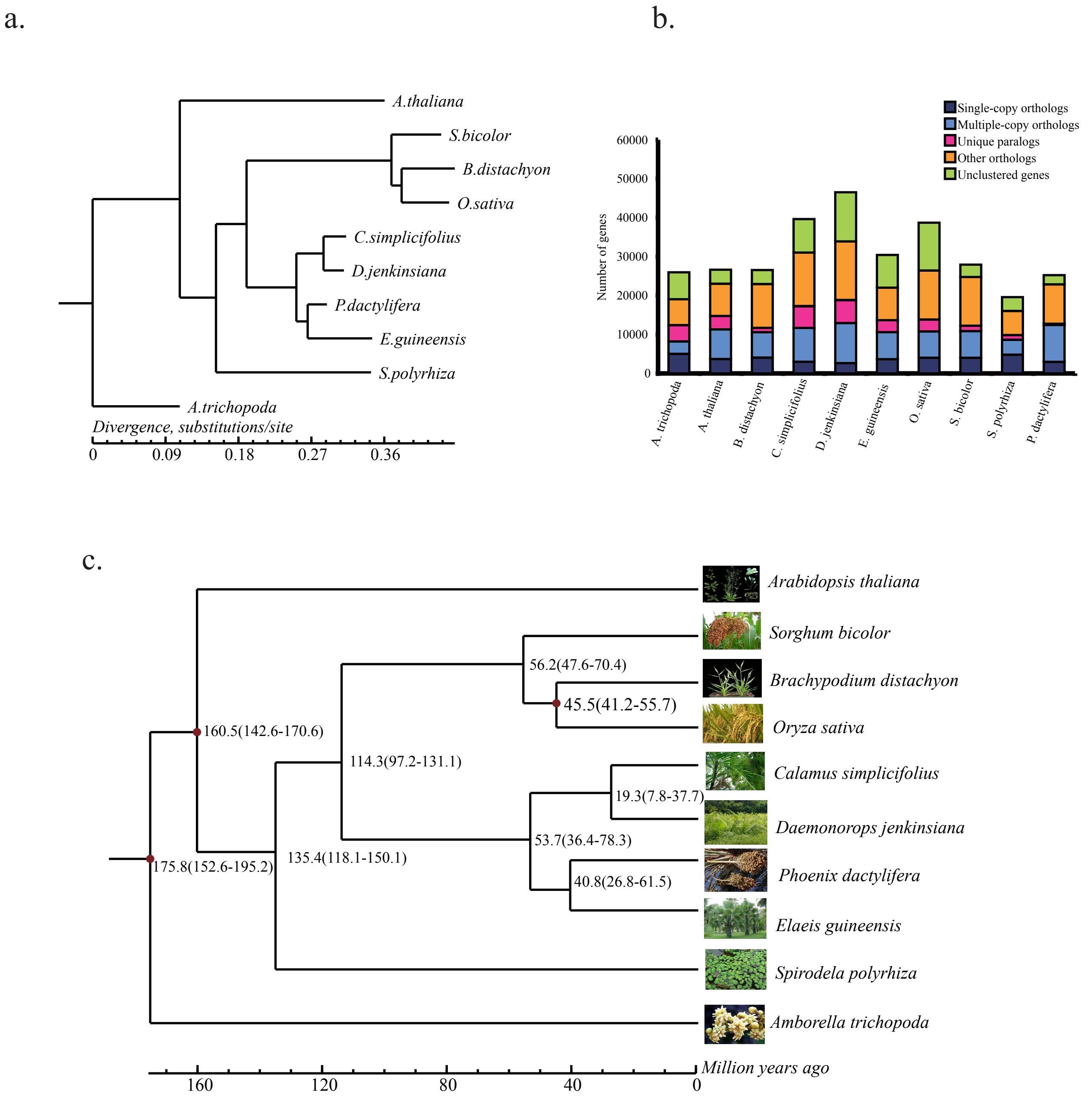

Additional Tables

Click here to access/download
Supplementary Material
Additional Files-R2-603.xlsx

Click here to access/download
Supplementary Material
Additional Figures-603.docx
