# Supplementary material for: The chromosome-level genome assemblies of two rattans (Calamus simplicifolius and Daemonorops jenkinsiana)
Source: Gigascience. 2018 Aug 7;7(9):giy097. doi: 10.1093/gigascience/giy097 (PMC6117794; doi:10.1093/gigascience/giy097)
Supplement: GIGA-D-18-00152_Revision_2.pdf [file giy097_giga-d-18-00152_revision_2.pdf]

GigaScience

The chromosome-level genome assemblies of two rattans (Calamus simplicifolius and Daemonorops jenkinsiana)

--Manuscript Draft--

**Manuscript Number:**	GIGA-D-18-00152R2
**Full Title:**	The chromosome-level genome assemblies of two rattans (Calamus simplicifolius and Daemonorops jenkinsiana)
**Article Type:**	Data Note
**Funding Information:**	the Sub-Project of the National Science and Technology Support Plan of the Twelfth Five-Year Plan in China (2015BAD04B03) — Prof. Hansheng Zhao
**Abstract:**	Background: Calamus simplicifolius and Daemonorops jenkinsiana are two representative rattans, the most significant material sources for the rattan industry. However, the lack of reference genome sequences is a major obstacle for basic and applied biology on rattan.<br><br>Findings: We produced two chromosome-level genome assemblies of C. simplicifolius and D. jenkinsiana using Illumina, PacBio, and Hi-C sequencing data. A total of ~730 Gb and ~682 Gb of raw data covered the predicted genome lengths (~1.98 Gb of C. simplicifolius and ~1.61 Gb of D. jenkinsiana) to ~372× and ~426× read depths, respectively. The two de novo genome assemblies, of ~1.94 Gb and ~1.58 Gb, were generated with scaffold N50s of ~160 Mb and ~119 Mb in C. simplicifolius and D. jenkinsiana, respectively. The C. simplicifolius and D. jenkinsiana genomes were predicted to harbor 51,235 and 53,342 intact protein-coding gene models, respectively. BUSCO evaluation demonstrated that genome completeness reached 96.4% and 91.3% in the C. simplicifolius and D. jenkinsiana genomes, respectively. Genome evolution showed that four Arecaceae plants clustered together, and the divergence time between the two rattans was ~19.3 Mya. Additionally, we identified 193 and 172 genes involved in the lignin biosynthesis pathway in the C. simplicifolius and D. jenkinsiana genomes, respectively.<br><br>Conclusions: We present the first de novo assemblies of two rattan genomes (C. simplicifolius and D. jenkinsiana). These data will not only provide a fundamental resource for functional genomics, particularly in promoting germplasm utilization for breeding, but also serve as reference genomes for comparative studies between and among different species.
**Corresponding Author:**	Hansheng Zhao<br>International Center for Bamboo and Rattan<br>Beijing, Beijing CHINA
**Corresponding Author Secondary Information:**	
**Corresponding Author's Institution:**	International Center for Bamboo and Rattan
**Corresponding Author's Secondary Institution:**	
**First Author:**	Hansheng Zhao
**First Author Secondary Information:**	
**Order of Authors:**	Hansheng Zhao
	Songbo Wang
	Jiongliang Wang
	Chuanhai Chen
	Shijie Hao

Powered by Editorial Manager® and ProduXion Manager® from Aries Systems Corporation

	Lianfu Chen
	Benhua Fei
	Kai Han
	Rongsheng Li
	Chengcheng Shi
	Huayu Sun
	Sining Wang
	Hao Xu
	Kebin Yang
	Xiurong Xu
	Xuemeng Shan
	Jingjing Shi
	Aiqin Feng
	Guangyi Fan
	Xin Liu
	Shancen Zhao
	Chi Zhang
	Qiang Gao
	Zhimin Gao
	Zehui Jiang
**Order of Authors Secondary Information:**	
**Response to Reviewers:**	Scott Edmunds<br>Executive Editor<br>GigaScience<br><br>25 July 2018<br><br>Dear Dr. Scott,<br><br>Re: Manuscript reference No. GIGA-D-18-00152R2<br>Please find attached a revised version of our manuscript "The chromosome-level genome assemblies of two rattans (Calamus simplicifolius and Daemonorops jenkinsiana)", which we would like to resubmit for publication as a research article in GigaScience.<br><br>The comments of two Reviewers were highly insightful and enabled us to improve the quality of our manuscript. In the following pages are our point-by-point responses to each of the comments and suggestions of the Reviewer.<br><br>The latest manuscript was revised by our team and AJE experts. We hope that the new submission and our accompanying responses will be sufficient to make our manuscript suitable for publication in GigaScience.<br><br>We shall look forward to hearing from you at your earliest convenience.<br><br><br>Yours sincerely,<br><br>Prof. Hansheng Zhao<br>Address: No. 8, Fu Tong Dong Da Jie, Chaoyang District, Beijing 100102, P.R. China<br>Tel: +86-010-8478 9804

Powered by Editorial Manager® and ProduXion Manager® from Aries Systems Corporation

Fax: +86-010-8478 9802
E-mail: zhaohansheng@icbr.ac.cn

Responses to the comments of Reviewer #1

The revised manuscript has addressed my comments properly. By merging two genomes together, the authors also did more survey into the biology of the Rattan, which also made the story more interesting. There are a few other points the authors could improve before publication:

1. A new section related to the lignin biosynthesis pathway was added in the revision. However, the concept of the copy number of a gene family is not clear. For example, the first gene family from Table 3 "4CL" has 12 paralogue genes from EnsemlPlants. They are normally interpreted as 12 gene members from the "4CL" family - not 12 copies of a particular gene "4CL". The current description is quite misleading. Please revise.

Response: Thank you for this excellent suggestion. According to your suggestion, we have revised the description, as follows:

“Each gene family contained multiple gene members with an average of ~15 and ~13 gene members per family in C. simplicifolius and D. jenkinsiana, respectively. The total size of the gene families in the lignin biosynthesis pathway contained 193 and 172 gene members in C. simplicifolius and D. jenkinsiana, respectively. Peroxidase (POD), as the most gene member, was detected both in the two rattans. As the least gene member, phenylalanine ammonia-lyase (PAL) was identified in C. simplicifolius and coumarate 3-hdroxylase (C3H) and cinnamate 4-hydroxylase (C4H) were detected in D. jenkinsiana.”

2. The name of the package for Hi-C data analysis is JuiceBox, not "Juicerbox". Besides, many citations in this section were wrongly labeled. Please check.

Response: Thank you for this excellent suggestion. According to your suggestion, we have revised the description, as follows:

“As shown in Fig. 2, the contact maps were visualized by JuiceBox (version 1.5.2).”

Responses to the comments of Reviewer #2

Reviewer #2: The authors have addressed all of our original comments in a detailed and satisfactory manner that includes the inclusion of substantially more text and new tables etc. This has resulted in a much improved paper that has increased relevance to plant breeders and users of rattan and related crops.

Response: Thank you for satisfying our revision and previous suggestions.

Additional Information:	
**Question**	**Response**
Are you submitting this manuscript to a special series or article collection?	No
**Experimental design and statistics**<br><br>Full details of the experimental design and statistical methods used should be given in the Methods section, as detailed in our Minimum Standards Reporting Checklist.	Yes

Information essential to interpreting the data presented should be made available in the figure legends.<br><br>Have you included all the information requested in your manuscript?	
**Resources**<br><br>A description of all resources used, including antibodies, cell lines, animals and software tools, with enough information to allow them to be uniquely identified, should be included in the Methods section. Authors are strongly encouraged to cite Research Resource Identifiers (RRIDs) for antibodies, model organisms and tools, where possible.<br><br>Have you included the information requested as detailed in our Minimum Standards Reporting Checklist?	Yes
**Availability of data and materials**<br><br>All datasets and code on which the conclusions of the paper rely must be either included in your submission or deposited in publicly available repositories (where available and ethically appropriate), referencing such data using a unique identifier in the references and in the "Availability of Data and Materials" section of your manuscript.<br><br>Have you have met the above requirement as detailed in our Minimum Standards Reporting Checklist?	Yes

Powered by Editorial Manager® and ProduXion Manager® from Aries Systems Corporation

Manuscript
Click here to view linked References
Click here to access/download;Manuscript;25Jul_mansucript_rattan_genom

The chromosome-level genome assemblies of two rattans (*Calamus simplicifolius* and *Daemonorops jenkinsiana*)

Hansheng Zhao[1#], Songbo Wang[2,5#], Jiongliang Wang[1#], Chuanhai Chen[2#], Shijie Hao[3], Lianfu Chen[1], Benhua Fei[1], Kai Han[3], Rongsheng Li[4], Chengcheng Shi[3], Huayu Sun[1], Sining Wang[1], Hao Xu[1], Kebin Yang[1], Xiurong Xu[1], Xuemeng Shan[1], Jingjing Shi[1], Aiqin Feng[2], Guangyi Fan[3], Xin Liu[3], Shancen Zhao[2,5], Chi Zhang[2,5], Qiang Gao[2*], Zhimin Gao[1*], and Zehui Jiang[1*]

[1] State Forestry Administration Key Open Laboratory on the Science and Technology of Bamboo and Rattan, Institute of Gene Science for Bamboo and Rattan Resources, International Center for Bamboo and Rattan, Futongdong Rd, WangJing, Chaoyang District, Beijing 100102, China;

[2] BGI Genomics, BGI-Shenzhen, Building No. 7, BGI Park, No. 21 Hongan 3rd Street, Yantian District, Shenzhen 518083, China;

[3] BGI-Qingdao, No. 2877, Tuanjie Road, Sino-German Ecopark, Qingdao, Shandong 266555, China;

[4] Research Institute of Tropical Forestry, Chinese Academy of Forestry, Guangshanyi Rd, Tianhe District, Guangzhou 510000, China;

[5] State Key Laboratory of Agricultural Genomics, BGI-Shenzhen, No. 7, Pengfei Road, Dapeng District, Shenzhen 518120, China.

[#] These authors contributed equally to this work.

[*] To whom correspondence should be addressed: Qiang Gao (gaoqiang@bgi.com), Zhimin Gao (gaozhimin@icbr.ac.cn), and Zehui Jiang (jiangzehui@icbr.ac.cn)

ORCIDs: Hansheng Zhao: 0000-0002-5405-0375; Jiongliang Wang: 0000-0002-0947-4582; Benhua Fei: 0000-0003-0764-2762; Rongsheng Li: 0000-0001-5253-4053; Huayu Sun: 0000-0002-6532-7018; Sining Wang: 0000-0002-3532-2777; Xiurong Xu: 0000-0002-7199-9961;

Guangyi Fan: 0000-0001-7365-1590; Shancen Zhao: 0000-0001-8779-6969; Zhimin Gao: 0000-0003-4464-7159; Zehui Jiang: 0000-0002-2696-5500.

Manuscript type: Data note

Abstract

Background: *Calamus simplicifolius* and *Daemonorops jenkinsiana* are two representative rattans, the most significant material sources for the rattan industry. However, the lack of reference genome sequences is a major obstacle for basic and applied biology on rattan.

Findings: We produced two chromosome-level genome assemblies of *C. simplicifolius* and *D. jenkinsiana* using Illumina, PacBio, and Hi-C sequencing data. A total of ~730 Gb and ~682 Gb of raw data covered the predicted genome lengths (~1.98 Gb of *C. simplicifolius* and ~1.61 Gb of *D. jenkinsiana*) to ~372× and ~426× read depths, respectively. The two *de novo* genome assemblies, of ~1.94 Gb and ~1.58 Gb, were generated with scaffold N50s of ~160 Mb and ~119 Mb in *C. simplicifolius* and *D. jenkinsiana*, respectively. The *C. simplicifolius* and *D. jenkinsiana* genomes were predicted to harbor 51,235 and 53,342 intact protein-coding gene models, respectively. BUSCO evaluation demonstrated that genome completeness reached 96.4% and 91.3% in the *C. simplicifolius* and *D. jenkinsiana* genomes, respectively. Genome evolution showed that four Arecaceae plants clustered together, and the divergence time between the two rattans was ~19.3 Mya. Additionally, we identified 193 and 172 genes involved in the lignin biosynthesis pathway in the *C. simplicifolius* and *D. jenkinsiana* genomes, respectively.

Conclusions: We present the first *de novo* assemblies of two rattan genomes (*C. simplicifolius* and *D. jenkinsiana*). These data will not only provide a fundamental resource for functional genomics, particularly in promoting germplasm utilization for breeding, but also serve as reference genomes for comparative studies between and among different species.

Keywords: Rattan, *Calamus simplicifolius*, *Daemonorops jenkinsiana*, whole genome sequencing, genome assembly, annotation

Background

Rattan is one of the world's most important nontimber forest products and represents a major lineage of climbing palms occurring naturally in the Old World [1]. A recent study has indicated that rattan is classified into 11 genera within the tribe Calameae and subfamily Calamoideae of the family Arecaceae. Broadly, rattan consists of 631 species, which occur in the same genera as nonclimbing palms [2]. Among all these genera, *Calamus* (NCBI Taxon ID: 4711) and *Daemonorops* (NCBI Taxon ID: 93268) are the most diverse, accounting for ~65% and ~20% of rattan species [3], respectively. These two genera are also the most important material sources, providing more than 95% of the canes produced by the rattan industry. More than 5 million people depend economically on rattan, and approximately 7 billion US dollars per year is made in the rattan industry, including domestic industrial production, the international cane trade, cane splitting, plaiting materials, baskets, seats and furniture [4]. Attention to the development of genetic breeding techniques in rattan is increasing, and the area of planted rattan is expected to gradually exceed that of natural rattans within a few years.

Calamus simplicifolius (NCBI Taxon ID: 746888) is a deeply developed rattan species indigenous to China (Fig. 1a) that generally forms an open cluster of vigorous, unbranched stems up to 50 m long and ~15 mm in diameter [5,6]. An endemic rattan of Hainan Island, *C. simplicifolius* can produce high-quality canes of medium diameter for binding and weaving in the rattan industry [5]. Furthermore, *Daemonorops jenkinsiana* (NCBI Taxon ID: 1510057), a representative species of high-climbing evergreen rattan, is one of the rattan species in the *Daemonorops* genus (Fig. 1b), which naturally grows in lowland rain forests below 1 km, from Bangladesh, Bhutan, Cambodia, India, Laos, Myanmar, Nepal, Thailand, and Vietnam to Southeast China [2]. *D. jenkinsiana* produces a dense cluster of vigorous stems that can be up to 50 m long and ~30 mm in diameter with internodes up to 40 cm long [6]. The two most productive rattan species, *C. simplicifolius* and *D. jenkinsiana*, are cultivated in areas with latitudes less than 23°30' N in China, i.e., Hainan Island, Guangdong, Guangxi, Yunnan, Fujian and other areas of southern China. Their established planting areas have been estimated at more than 1,000 ha [5].

C. simplicifolius and *D. jenkinsiana* have various applications and enormous development potentials. These species are interesting mainly because of their canes, which have high pliability and remarkable

durability. Molecular breeding technologies have been employed to meet the growing requirements for rattan quality and quantity. However, the lack of known genetic structure underlying the important traits of rattan has severely hampered a comprehensive understanding of its molecular biology for scientific research and actual production, as well as the in-depth performance of comparative genome analyses between and among related species. Thus, we here report the two *de novo* genome assemblies of *C. simplicifolius* and *D. jenkinsiana* using the latest sequencing (Illumina and PacBio) and mapping (Hi-C) technologies. With the availability of these two chromosome-level reference genomes in rattan, many comparative genome analyses and other downstream applications will become feasible, such as the development of biomarkers, the identification of functional genes, and molecular design breeding. Additionally, high-quality genome assemblies of rattan will facilitate genomic, transcriptomic, and metabolomic analyses of its material traits. As genes of possible specific interest for material improvement, members of gene families involved in lignin biosynthesis in rattan are identified here. These studies lay a foundation for future research on the utilization of these genes to improve rattan quality and diversity within rattan germplasm.

Data Description

DNA isolation, library construction, and sequencing

Young leaves at the vegetative growth stage were collected from *C. simplicifolius* and *D. jenkinsiana* in Spring 2015 at the Research Institute of Tropical Forestry of the Chinese Academy of Forestry in the city of Guangzhou, Guangdong Province, China (N: 23°11′29″, E: 113°22′40″, 87 M). Total DNA was isolated and extracted using DNeasy Plant Mini Kits (Qiagen) based on the manufacturer’s instructions. Genomic DNA was purified according to the isolation protocol for high-molecular-weight nuclear DNA. Multiple DNA libraries were constructed [7] and sequenced on the Illumina HiSeq 4000 and PacBio Sequel platforms (Table 1). Briefly, we built three libraries with different insert sizes (270 bp, 500 bp and 800 bp) for paired-end (PE) sequencing and four libraries with different insert sizes (2 kb, 5 kb, 10 kb and 20 kb) for mate-pair (MP) sequencing, based on the standard Illumina protocol [8]. We also constructed five PacBio Sequel libraries with a 20 kb insert size, following the standard PacBio protocol. After data cleaning and data preprocessing, we obtained 494.08 Gb of clean data (322.3 Gb PE reads,

93.4 Gb MP reads, and 78.38 Gb PacBio data), representing 252× coverage of the *C. simplicifolius* genome, and 426.17 Gb of clean data (244.58 Gb PE reads, 103.21 Gb MP reads, and 78.38 Gb PacBio data), representing 266 × coverage of the *D. jenkinsiana* genome.

As another analysis parallel to the library construction of Illumina and PacBio, two Hi-C libraries were constructed for *C. simplicifolius* and *D. jenkinsiana* using same young leaves in BGI-Qingdao[9]. We used the *Mbo*I restriction enzyme to digest the DNA after its conformation was fixed by formaldehyde and then repaired the 5′ overhangs using biotinylated residues. Following the ligation of blunt-end fragments in situ, the isolated DNA was reverse-crosslinked, purified and filtered for biotin-containing fragments. Subsequently, DNA fragment end repair, adaptor ligation and PCR were performed, in that order. Then, the standard circularization step of BGISEQ-500 was carried out, and sequencing was performed using BGISEQ-500 sequencing with 100PE reads [10,11]. Thus, we obtained ~6.7 Gb and ~13.1 Gb of valid data after ~148 Gb and ~154 Gb of raw data were evaluated and analyzed using HiC-Pro (version 2.8.0_devel) [12] in *C. simplicifolius* and *D. jenkinsiana*, respectively (Table 1).

Genome survey

An understanding of the genomic characteristics of a given new species, i.e., genome size and heterozygosity, facilitates the development of a customized sequencing and assembly strategy. Thus, the genome size was estimated using four independent methods: a script of KmerSpectrumPlot.pl in ALLPATHS-LG (version r52488) [13], GCE (Genome Characteristics Estimation, released 20150107, ftp://ftp.genomics.org.cn/pub/gce), JELLYFISH (version 2.0) [14] and flow cytometry (Additional Tables S1-2 and Figs. S1-2). In our genome survey, ~98 Gb and ~60 Gb of sequences were generated from short-insert-size libraries for *C. simplicifolius* and *D. jenkinsiana*, respectively. During data preprocessing, low-quality reads (more than 40% of bases with Q<13 in a given read) were filtered out using NGS QC Toolkit (version 2.3.3) [15] with the default parameters. The combination (Additional Table S1) showed that the final predicted genome sizes were ~1.98 Gb for *C. simplicifolius* and ~1.61 Gb for *D. jenkinsiana*, and the related heterozygosity was estimated at 1.32%~1.52% and 1.19~1.31%,

respectively. Thus, the genome survey suggested that these two rattan genomes might be suitable for a hybrid sequencing strategy using the Illumina and PacBio data.

Hybrid *de novo* genome assembly using Illumina, PacBio and Hi-C sequencing data

During preprocessing of the Illumina data, we filtered out low-quality reads and adaptor sequences. Thus, ~416 Gb and ~348 Gb of clean data were generated for *C. simplicifolius* and *D. jenkinsiana*, respectively. For the PacBio data, we used MECAT (release 20170627) to correct errors [16] with the following parameters: -x 0 -i 0 -t 60 -r 0.8 -a 1000 -c 5 -l 2000. Thus, we obtained ~52 Gb and ~32 Gb of corrected PacBio data for *C. simplicifolius* and *D. jenkinsiana*, respectively. Subsequently, FALCON (version 0.3) [17] was chosen to perform the first assembly of the initial contigs of the two rattans. As shown in Additional Table S3, two assemblies using different parameters were generated for the *C. simplicifolius* genome: a 1.59 Gb assembly with a contig N50 of 67.2 kb (~80% of the estimated genome size) and a 1.53 Gb assembly with a contig N50 of 66.7 kb (~77% of the estimated genome size). Additionally, a 1.27 Gb assembly with a contig N50 of 81.5 kb (~79% of estimated genome size) was obtained for *D. jenkinsiana*. The performance of MECAT for the two rattans was still not of sufficiently high quality. Thus, we considered that the incompleteness of the assembled scaffolds and low contig N50 might be due to high heterozygosity (1.32%~1.52% for *C. simplicifolius* and 1.19~1.31% for *D. jenkinsiana*), a high proportion of repeat sequences (54.15% for *C. simplicifolius* and 70% of *D. jenkinsiana*, see subsequent analysis for details), and inadequate sequencing depth, which was ~26× and ~20× of effective PacBio data after error correction, respectively. Therefore, taking the above findings into account, we conducted hybrid *de novo* genome assembly of *C. simplicifolius* and *D. jenkinsiana* using the Illumina and PacBio sequencing data. First, Platanus (version 1.2.4) [18], a *de novo* genome assembler for highly heterozygous data, was carried out to assemble the fragment PE reads into contigs by constructing De Bruijn graphs with an automatically optimized *k*-mer size. Second, the corrected PacBio reads and the assembled contigs were subjected to DBG2OLC (release 20150611) [19] to construct scaffolds with the following parameters: DBG2OLC Contigs contig.fa LD 0 K 17 KmerCovTh 4 MinOverlap 25 AdaptiveTh 0.007 RemoveChimera 1 f scaffold.fa. Hence, we obtained

~1.92 Gb and ~1.56 Gb of initial assembly sequences for *C. simplicifolius* and *D. jenkinsiana*, respectively. Third, a polishing process before the SSPACE process was performed with reference to the consensus analysis of DBG2OLC (Additional Table S4); this step contributed to enhancing the quality of the genome assembly and reducing errors in the SSPACE process. Then, the assemblies were elongated by SSPACE (version 3.0) [20] using the MP reads, and some gaps were filled using the Illumina and PacBio data by GapCloser (version 1.12) [21] and PBJelly (release 20150824) [22]. Thus, we obtained an assembly of 1.96 Gb, containing 5,116 scaffolds with a contig N50 length of 107 kb and a scaffold N50 of 803 kb for *C. simplicifolius*, and we obtained an assembly of ~1.60 Gb for *D. jenkinsiana* with N50 lengths of 108 kb and 784 kb for the contigs and scaffolds, respectively (Table 2).

Subsequently, the valid Hi-C data together with the above assembly were processed by the 3D-DNA pipeline (version 170123) [23] to produce chromosome-level scaffolds. We obtained an explicit contact pattern, which implied a reasonably accurate chromosome-level assembly. As shown in Fig. 2, the contact maps were visualized by Juicebox (version 1.5.2) [24]. The lengths of the longest 12 chromosome-level scaffolds for the *C. simplicifolius* assembly and the 13 for the *D. jenkinsiana* assembly are presented in Additional Table S5. The total lengths of the pseudochromosomes accounted for 92.08% and 92.01% of the *C. simplicifolius* and *D. jenkinsiana* genomes, with scaffold N50 values of 169 Mb and 119 Mb, respectively.

Genome evaluation

Three independent methods were used to evaluate the accuracy and completeness of the *C. simplicifolius* and *D. jenkinsiana* assemblies. First, two genome features were summarized: the percentage of ambiguous bases (Ns) and GC content. The results showed a low percentage of Ns (~0.6% for *C. simplicifolius* and ~0.7% for *D. jenkinsiana*) in each genome, and the overall GC contents (41.07% for *C. simplicifolius* and 41.78% for *D. jenkinsiana*) were similar to those of the related transcriptomic data (41.68% for *C. simplicifolius* and 41.89% for *D. jenkinsiana*). Then, the unigenes assembled from the RNA-Seq data were aligned to the assembly using BLAT (version 1.0) [25] with the default parameters. The alignment results showed that more than 90% of the sequences in one scaffold could

be aligned with assembly (92.89% of *C. simplicifolius* and 81.81% of *D. jenkinsiana*) (Additional Table S6). Last, the completeness of the two rattan assemblies was evaluated using BUSCO (version 3.0) [26], which quantitatively assesses genome completeness using evolutionarily informed expectations of gene content from near-universal single-copy orthologs. The BUSCO results showed that 96.4% of conserved BUSCO proteins (embryophyta_odb9) were detected in the *C. simplicifolius* assembly, including 3.8% of fragment BUSCO proteins. Additionally, 87.3% and 4.0% of the conserved BUSCO proteins were identified as complete and fragment proteins in *D. jenkinsiana*, respectively (Additional Table S7).

Repeat annotation

Before protein-coding gene model prediction, transposable elements (TEs) and tandem repeats were identified in the *C. simplicifolius* and *D. jenkinsiana* assemblies. We adopted two independent approaches to predict repetitive elements: homology-based annotation and *de novo* methods. In the homology-based annotation, TEs were identified using RepeatMasker (v4.0.5) and RepeatProteinMasker (v4.0.5) [27] via searching against the Repbase library (release 201712) [28]. In the *de novo* annotation, a *de novo* repeat library was constructed using RepeatModeler (v1.0.8) [29] and LTR_FINDER [30] after eliminating contaminants and multicopy genes. Then, RepeatMasker was used to categorize the genome sequences against the *de novo* repeat library. Additionally, tandem repeat sequences were identified by Tandem Repeat Finder (version 4.09) [31] with the following parameters: "Match = 2, Mismatch = 7, Delta = 7, PM = 80, PI = 10, Minscore = 50 and MaxPeriod = 2000". Overall, the results showed that long terminal repeat (LTR) was the most abundant repeat type and that SINE and LINE, two non-LTR retrotransposons, had the lowest proportions in the two rattan assemblies (Additional Table S8). TEs accounted for 54.15% and 70% of the *C. simplicifolius* and of *D. jenkinsiana* assemblies, respectively, and the sequence divergence of TEs indicated that the *de novo*-predicted repeats were more recently active than the Repbase-predicted repeats (Fig. 3).

RNA sample collection, library construction, and transcriptome assembly

Four samples of the distal cirrus at three developmental stages were collected from *C. simplicifolius* and *D. jenkinsiana*. Each sample had three biological replicates (Additional Table S9). Because this experiment was a part of the rattan genome project, the location of RNA sampling was consistent with that of DNA sampling. Based on the manufacturer's instructions, RNA was isolated using TRIzol Reagent Solution (Invitrogen, Carlsbad, CA, USA), and the purity and concentration were determined with a NanoDrop 2000 spectrophotometer. Reverse transcription was conducted with a Reverse Transcription System (Promega, USA). The extracted RNA was treated with RNase-free DNase I for 30 min at 37 °C to remove residual DNA, as described previously [32], and then, the pooled libraries were sequenced using the BGISEQ-500 platform with short 100PE reads. When preprocessing the transcriptomic data, adaptor sequences and low-quality reads were filtered using SOAPnuke (version 1.5.6) [33] with the following parameters: "-n 0.001 -l 20 -q 0.4 -Q 2". The clean reads of all samples were assembled using Trinity (version 2.0.6)[34] with the following parameters: (1) group_pairs_distance 500, (2) min_contig_length 200, (3) min_kmer_cov 2, (4) min_glue 2, (5) bfly_opts -V 5, (6) edge-thr=0.1, (7) stderr, and (8) SS_lib_type RF. Then, the outputs of Trinity were clustered to generate a single set of nonredundant references using TGI Clustering Tool (version v2.0.6) [35] with the following parameters: (1) a minimum of 95% identity between the contigs, (2) a minimum of 35 overlapping bases, (3) a minimum score of 35, and (4) a maximum of 20 unmatched overhanging bases at the sequence ends. Ultimately, the assembled transcripts were divided into two classes based on sequence similarity: clusters (prefixed with 'CL') and singletons (prefixed with 'unigene'). In each cluster, the sequence similarity regions between the transcripts were more than 70%, and the transcripts were spliced isoforms from a gene or a paralogous gene. Additionally, all unigenes were used in subsequent analyses.

Gene modeling and prediction

We performed an integrated prediction of intact protein-coding gene models using three independent approaches [7], i.e., *de novo* prediction, homology-based method, and RNA-Seq approach. The repeat masked assembly was first annotated by AUGUSTUS (version 3.3) with the default parameters [36]; this program is a *de novo* predictor based on a self-trained model. After the training data were optimized

and multiple trainings were performed, 85,246 and 87,613 gene models were predicted for *C. simplicifolius* and *D. jenkinsiana*, respectively. In the homology-based prediction, we used seven species as reference datasets, i.e., *Elaeis guineensis*, *Phoenix dactylifera*, *Brachypodium distachyon*, *Oryza sativa*, *Setaria italica*, *Sorghum bicolor*, and *Zea mays* (for individual genome versions, see Availability of supporting data). Their protein sequences were downloaded from the ENSEMBL database [37] and aligned to the *C. simplicifolius* and *D. jenkinsiana* assembly using TBLASTN (version 2.2.26) [38] with an E-value cutoff of 1e-5. Then, splicing patterns were generated by GeneWise (version 2.0) [39]. In the RNA-Seq analysis, HISAT2 (version 2.0.2) [40] was used to identify exon-intron splicing junctions and refine the alignment of the RNA-Seq reads to the genome. We then used Cufflinks (version 2.2.1) [41] to define 56,024 and 58,134 protein-coding gene models in *C. simplicifolius* and *D. jenkinsiana*, respectively (Additional Table S10).

Lastly, we integrated the evidence from the three above independent predictions using MARKER (version 2) [42]. The final prediction results showed that 51,235 and 53,342 intact protein-coding gene models were predicted as consensus gene sets in *C. simplicifolius* and *D. jenkinsiana*, respectively.

Annotation evaluation and gene function prediction

We evaluated the predicted annotations using two independent methods: gene function evaluation and completeness evaluation by BUSCO. In the gene function evaluation, we assessed the agreement of the predicted annotations with protein alignment searches for homologous proteins in closely related species and manual annotations. The results of alignments against five authoritative protein databases (Additional Table S11) indicated that 5.34% and 2.89% of the predicted gene models were identified as unannotated genes in *C. simplicifolius* and *D. jenkinsiana*, respectively. These protein databases included the NCBI nonredundant protein database (release 20180313) [43], SWISS-PROT (release 201801) [44], GO (release 20131030) [45], KEGG (dataset v81) [46], and InterPro (dataset v.53) [47]. Additionally, the BUSCO evaluation showed that 88.7% and 91.3% of conserved BUSCO proteins (embryophyta_odb9) were present in *C. simplicifolius* and *D. jenkinsiana*, respectively. Among the conserved BUSCO proteins, 76.2% and 81.2% were complete. Furthermore, the four types of

noncoding RNA genes, i.e., tRNA, rRNA, miRNA and snRNA, were also predicted (Additional Table S12).

Gene family construction and rattan-specific gene families

In our study, we performed a pairwise sequence comparison to predict orthologous genes at the genome level. This method is rapid and generally deals well with large amounts of data. A popular BLAST-based approach, OrthoMCL (version 2.0.9) [48], was used to identify orthologous genes in *C. simplicifolius* and *D. jenkinsiana* with an E-value cutoff of 1e-5 and a percent match cutoff of 80 (i.e., query and match were required to overlap on more than 80% of the query and match sequence length). Markov chain clustering was also used with a default inflation parameter in an all-to-all BLASTP analysis of entries for other 8 plants, i.e., *Amborella trichopoda*, *E. guineensis*, *A. thaliana*, *B. distachyon*, *O. sativa*, *Spirodela polyrhiza*, *P. dactylifera* and *S. bicolor* (for individual genome versions, see Availability of supporting data). Among the 30,936 gene families identified in all 10 species, 44,700 and 44,537 orthologous genes were detected in the *C. simplicifolius* and *D. jenkinsiana* genomes, respectively. Approximately 6,132 (19.8%) gene families common to all 10 species as well as 2,366 and 2,707 specific gene families were detected in *C. simplicifolius* and *D. jenkinsiana*, respectively (Fig. 4b). Additionally, the results showed that 637 gene families were specific to the rattans. These rattan-specific gene families were enriched in gene ontology (GO) categories related to component membrane and transcription factor activity (Additional Table S13) and in KEGG pathways related to plant-pathogen interaction and plant hormone signal transduction (Additional Table S14).

Phylogenetic analysis and divergence time

We obtained 962 single-copy orthologous genes derived from entire gene families that were conserved among the species to facilitate an understanding of the evolutionary relationships of rattans with other species. First, multiple alignments of protein sequences were conducted by MUSCLE (version 3.8.31) [49], and then, a CDS alignment was constructed based on the protein alignments. Subsequently, all aligned CDS sequences were concatenated to generate a supergene for each species using an in-house Perl script. Thus, we extracted the nucleotides at position 2 (phase 1) of each codon to construct the

phylogenetic tree using RAxML (version 8.2.3) [50] with the model "GTRGAMMA". The results showed that four Arecaceae plants were located in a cluster, which comprised two independent sister branches with one containing *C. simplicifolius* and *D. jenkinsiana* and the other containing *E. guineensis* and *P. dactylifera* (Fig. 4a).

Moreover, we used the MCMCTree program of PAML (version 4.5) [51] to estimate the divergence times among *C. simplicifolius*, *D. jenkinsiana* and the other 8 plants with the following parameters: "-nsample 200000 -burnin 40000". The calibration times were derived from published times for the divergences of the reference species [52]. The results indicated that the divergence time between the two rattans was ~19.3 Mya, and for the other two Arecaceae species, *P. dactylifera* separated from *E. guineensis* at ~40.8 Mya (Fig. 4c).

Genome-wide identification of gene families involved in the lignin biosynthesis pathway

Lignins are a class of complex aromatic heteropolymers of monolignols that encrust and interact with the cellulose/hemicellulose matrix of the secondary cell wall. The aromatic lignin polymers are commonly composed of three monolignols, i.e., *p*-hydroxyphenyl (H), vanillin (G), and syringaldehyde (S)[53]. Thus, we performed genome-wide identification of 13 gene families involved in the lignin biosynthesis pathway of rattan using 8 genomes, i.e., *A. thaliana*, *B. distachyon*, *O. sativa*, *S. bicolor*, *Phyllostachys edulis*, *Populus trichocarpa*, *D. jenkinsiana*, and *C. simplicifolius.* Most genome sequences (*A. thaliana*, *B. distachyon*, *O. sativa*, *S. bicolor*, and *Po. trichocarpa*) were downloaded from the ENSEMBL database [54]. The genome sequence of *Ph. edulis* was downloaded from the Bamboo Genome Database [55]. Based on wide literature-based investigations, one hundred forty genes involved in the lignin biosynthetic pathway were collected based on experimental validation in previous studies (Additional Table S15); then, these known genes were used as query sequences for further gene identification. A BLAST search and domain analysis as described previously [54] were used in the genome-wide gene identification process. Briefly, we performed standard protein BLAST searches (version 2.2.26) against all genome sequences including those of the two rattans using the coding sequences of known genes with the following cutoff values: E-value <1e-10; identity >40%; and coverage rate >95% of query sequence. The filtered sequences were subsequently analyzed by

hmmsearch (version 3.1b2) using the Pfam-A.hmm database (released 2017/03/31), and unclear sequences with incomplete domains were discarded by manual correction. The results showed that the expansion of most lignin-related gene families was detected in the two rattans (Table 3). Each gene family contained multiple members, with an average of ~15 and ~13 gene members per family in *C. simplicifolius* and *D. jenkinsiana,* respectively. The total numbers of genes in the lignin biosynthesis pathway were 193 and 172 genes in *C. simplicifolius* and *D. jenkinsiana,* respectively. Peroxidase (POD), as the most common gene, was detected in both rattans. Among the least common genes, phenylalanine ammonia-lyase (PAL) was identified in *C. simplicifolius*, and coumarate 3-hydroxylase (C3H) and cinnamate 4-hydroxylase (C4H) were detected in *D. jenkinsiana*. The observed expansion of lignin biosynthesis genes in rattan could be due to the occurrence of a whole genome duplication (WGD) event, since WGD could provide more gene copies, which facilitates the evolution of genes with new functions [56].

Conclusion

Here, we report two chromosome-level reference genome sequences of rattan (*C. simplicifolius* and *D. jenkinsiana*) using multiple types of sequencing data and assembly technologies. These *C. simplicifolius* [50] and *D. jenkinsiana* [51] genomes should facilitate the *de novo* genome assembly and resequencing of other rattan species and serve as essential resources to identify regions providing suitable resolution in the evolutionary landscape by performing comparative studies between and among different species. The availability of two high-quality rattan genomes simplifies the identification of critical genes involved in the lignin biosynthesis pathway, which have potential importance for rattan growth and development. Therefore, these data pave the way for additional genomic studies in rattan and related plants.

Availability of supporting data

The datasets and materials supporting the results of this article are available in the *Giga*DB repository [57-59]. All raw genomic sequence reads from the BIGSEQ-500, Illumina and PacBio platforms and the transcriptome reads derived from multiple tissues have been uploaded and deposited in the European

Nucleotide Sequence Archive (EMBL-EBI) with the project accession nos. PRJEB24031 and PRJEB24829 for *C. simplicifolius* and *D. jenkinsiana*, respectively. Other data analyzed in this study included *A. trichopoda* (version 1.0), downloaded from the Amborella Genome Database (amborella.huck.psu.edu), and *E. guineensis* (version GCF_000442705.1), downloaded from NCBI. The remaining genomes were downloaded from the ENSEMBL database, including *E. guineensis* (version GCF_000442705.1), *Ph. dactylifera* (version 1.0), *B. distachyon* (version 3.1), *O. sativa* (version R498), *S. italica* (version 9.0), *S. bicolor* (version 3.1), *Z. mays* (version B73_RefGen_V4), *Ph. edulis* (version 2), *Po. trichocarpa* (JGI2.0.31), and *A. thaliana* (version: TAIR10).

Abbreviations

BUSCO: Benchmarking Universal Single-Copy Ortholog; GABR: Genome Atlas of Bamboo and Rattan; GO: Gene Ontology; KEGG: Kyoto Encyclopedia of Genes and Genomes; LTR: long terminal repeat; MP: mate-pair; NCBI: National Center for Biotechnology Information; PE: paired-end; RNA-Seq: RNA-sequencing; SRA: Sequence Read Archive; TE: transposable element; WGD: whole genome duplication.

Additional files

Additional Table S1: Evaluation of genome sizes of *C. simplicifolius* and *D. jenkinsiana*

Additional Table S2: The 17-mer frequency method estimated genome sizes of *C. simplicifolius* and *D. jenkinsiana*

Additional Table S3: Statistics of the assemblies using different assembly strategies

Additional Table S4: BUSCO evaluation of the polishing process

Additional Table S5: The chromosome-level lengths of the Hi-C assemblies for *C. simplicifolius* and *D. jenkinsiana*

Additional Table S6: Statistics of the quality assessment of the *C. simplicifolius* and *D. jenkinsiana* genomes

Additional Table S7: BUSCO evaluation of the *C. simplicifolius* and *D. jenkinsiana* genomes

Additional Table S8: Statistics of the predicted repetitive sequences in the *C. simplicifolius* and *D. jenkinsiana* genomes

Additional Table S9: Statistics of RNA libraries in the transcriptome assemblies

Additional Table S10: Statistics of the predicted protein-coding genes in the *C. simplicifolius* and *D. jenkinsiana* genomes

Additional Table S11: Statistics of functional annotations of the *C. simplicifolius* and *D. jenkinsiana* genomes

Additional Table S12: Statistics of the predicted noncoding RNAs in the *C. simplicifolius* and *D. jenkinsiana* genomes

Additional Table S13: GO analysis of rattan-specific gene families

Additional Table S14: KEGG analysis of rattan-specific gene families

Additional Table S15: A total of 140 genes in the lignin biosynthetic pathway experimentally validated in previous studies

Additional Figure S1: Evaluation of the genome sizes of *C. simplicifolius* and *D. jenkinsiana* by K-mer

Additional Figure S2: Evaluation of the genome size of *C. simplicifolius* and *D. jenkinsiana* by flow cytometry

Competing interests

The authors have declared that there are financial and nonfinancial competing interests in this study.

Funding

This work was supported by the Sub-Project of the National Science and Technology Support Plan of the Twelfth Five-Year Plan in China (Nos. 2015BAD04B03 and 2015BAD04B01), Fundamental Research Funds for the International Center for Bamboo and Rattan (No. 1632017018) and the Science Technology and Innovation Committee of Shenzhen Municipality (No JCYJ20160331190123578).

Author contributions

HSZ and RSL collected the samples; JLW, HYS, SNW, HX, KBY, XRX, XMS, and JJS constructed libraries; HSZ, SBW, CHC, LFC, AQF, CZ, and QG performed the genome assembly. SJH, KH, CCS, and GYF performed the Hi-C analysis. SBW and LFC performed the genome annotation; HSZ, SBW, LFC, and XL analyzed the genome data. HSZ and SBW wrote the manuscript; HSZ, SBW, XL, ZMG and ZHJ reviewed the manuscript. All the above authors have read and approved the final manuscript.

Acknowledgements

As a part of the Genome Atlas of Bamboo and Rattan (GABR), we wish to acknowledge the GABR Consortium members, partners, advisors, and supporters who have helped this project run smoothly.

Reference

1. Jiang Z. Bamboo and Rattan in the World. Beijing: China Forestry Publishing House. 2007.

2. International Network for Bamboo and Rattan. World Checklist of Bamboo and Rattans. Beijing: International Network of Bamboo and Rattan; 2017.

3. Larsen K. Genera Palmarum. A classification of palms based on the work of Harold E. Moore Jr. Nordic Journal of Botany. 1989;9:62–2.

4. Kumar HNK, Preethi SD, Chauhan JB. Studies on the *in vitro* propagation of *Calamus travancoricus*. Asian Journal of Plant Science and Research. 2012;2:137-179.

5. Li R, Yin G, Yang J, Zou W. Rattan sector in Hainan Island, China: a case study. Journal of Forestry Research. 2007;18:153–156.

6. eFloras Published on the Internet. Missouri Botanical Garden, St. Louis, MO, & Harvard University Herbaria, Cambridge, MA. 2008. http://www.eoras.org. Accessed 20 May 2017.

7. Peng Z, Lu Y, Li L, Zhao Q, Feng Q, Gao Z, et al. The draft genome of the fast-growing non-timber forest species moso bamboo (*Phyllostachys heterocycla*). Nature Genetics. 2013;45:456–61.

8. Edmunds SC. Hiseq 4000 Sequencing protocol. protocols.io. 2018. dx.doi.org/10.17504/protocols.io.q58dy9w.

9. Liu X. The pipeline of Hi-C assembly. protocols.io. 2018. dx.doi.org/10.17504/protocols.io.qradv2e.

10. Huang J, Liang X, Xuan Y, Geng C, Li Y, Lu H, et al. BGISEQ-500 Sequencing. protocols.io. 2018. dx.doi.org/10.17504/protocols.io.ps5dng6

11. Huang J, Liang X, Xuan Y, Geng C, Li Y, Lu H, et al. BGISEQ-500 WGS library construction. protocols.io. 2018. dx.doi.org/10.17504/protocols.io.pq7dmzn

12. Servant N, Varoquaux N, Lajoie BR, Viara E, Chen C, Vert JP, et al. HiC-Pro: an optimized and flexible pipeline for Hi-C data processing. Genome Biology. 2015;16:259.

13. Maccallum I, Przybylski D, Gnerre S, Burton J, Shlyakhter I, Gnirke A, et al. ALLPATHS 2: small genomes assembled accurately and with high continuity from short paired reads. Genome Biology. 2009;10:R103.

14. Marçais G, Kingsford C. A fast, lock-free approach for efficient parallel counting of occurrences of k-mers. Bioinformatics. 2011;27:764–770.

15. Patel RK, Jain M. NGS QC Toolkit: A Toolkit for Quality Control of Next Generation Sequencing Data. PloS one. 2012;7:e30619.

16. Xiao C, Chen Y, Xie S, Chen K-N, Wang Y, Han Y, et al. MECAT: fast mapping, error correction, and *de novo* assembly for single-molecule sequencing reads. Nature Methods. 2017;14:1072–4.

17. FALCON. https://github.com/PacificBiosciences/FALCON. Accessed 10 Sep. 2017.

18. Kajitani R, Toshimoto K, Noguchi H, Toyoda A, Ogura Y, Okuno M, et al. Efficient *de novo* assembly of highly heterozygous genomes from whole-genome shotgun short reads. Genome research. 2014;24:1384–95.

19. Ye C, Hill CM, Wu S, Ruan J, Ma Z. DBG2OLC: Efficient Assembly of Large Genomes Using Long Erroneous Reads of the Third Generation Sequencing Technologies. Scientific Reports. 2016;6:31900.

20. Hunt M, Newbold C, Berriman M, Otto TD. A comprehensive evaluation of assembly scaffolding tools. Genome Biology. 2014;15:R42.

21. Luo R, Liu B, Xie Y, Li Z, Huang W, Yuan J, et al. Erratum: SOAPdenovo2: an empirically improved memory-efficient short-read *de novo* assembler. GigaScience. 2015;4:30.

22. English AC, Richards S, Han Y, Wang M, Vee V, Qu J, et al. Mind the gap: upgrading genomes with Pacific Biosciences RS long-read sequencing technology. PloS one. 2012;7:e47768.

23. Dudchenko O, Batra SS, Omer AD, Nyquist SK, Hoeger M, Durand NC, et al. *De novo* assembly of the *Aedes aegypti* genome using Hi-C yields chromosome-length scaffolds. Science. 2017;356:92–5.

24. Zhao H, Dong L, Sun H, Li L, Lou Y, Wang L, et al. Comprehensive analysis of multi-tissue transcriptome data and the genome-wide investigation of GRAS family in *Phyllostachys edulis*. Scientific Reports. 2016;6:27640.

25. Kent WJ. BLAT--the BLAST-like alignment tool. Genome research. 2002;12:656–64.

26. Simão FA, Waterhouse RM, Ioannidis P, Kriventseva EV, Zdobnov EM. BUSCO: assessing genome assembly and annotation completeness with single-copy orthologs. Bioinformatics. 2015;31:3210–2.

27. Tarailo-Graovac M, Chen N. Using RepeatMasker to identify repetitive elements in genomic sequences. Current Protocol in Bioinformatics. Hoboken, NJ, USA: John Wiley & Sons, Inc; 2009;Chapter 4:Unit4.10–4.10.14.

28. Bao W, Kojima KK, Kohany O. Repbase Update, a database of repetitive elements in eukaryotic genomes. Mobile DNA. 2015;6:11.

29. RepeatModeler. http://www.repeatmasker.org/RepeatModeler/. Accessed 10 Sep. 2017.

30. Xu Z, Wang H. LTR_FINDER: an efficient tool for the prediction of full-length LTR retrotransposons. Nucleic Acids Research. 2007;35:W265–268.

31. Benson G. Tandem repeats finder: a program to analyze DNA sequences. Nucleic Acids Research. 1999;27:573–580.

32. Zhao H, Sun H, Li L, Lou Y, Li R, Qi L, et al. Transcriptome-based investigation of cirrus development and identifying microsatellite markers in rattan (*Daemonorops jenkinsiana*). Scientific Reports. 2017;7:46107.

33. Chen Y, Chen Y, Shi C, Huang Z, Zhang Y, Li S, et al. SOAPnuke: a MapReduce acceleration-supported software for integrated quality control and preprocessing of high-throughput sequencing data. GigaScience. 2018;7:1–6.

34. Haas BJ, Papanicolaou A, Yassour M, Grabherr M, Blood PD, Bowden J, et al. *De novo* transcript sequence reconstruction from RNA-seq using the Trinity platform for reference generation and analysis. Nature Protocol. 2013;8:1494–512.

35. Pertea G, Huang X, Liang F, Antonescu V, Sultana R, Karamycheva S, et al. TIGR Gene Indices clustering tools (TGICL): a software system for fast clustering of large EST datasets. Bioinformatics. 2003;19:651–652.

36. Stanke M, Morgenstern B. AUGUSTUS: a web server for gene prediction in eukaryotes that allows user-defined constraints. Nucleic Acids Research. 2005;33:W465–467.

37. Zerbino DR, Johnson N, Juetteman T, Sheppard D, Wilder SP, Lavidas I, et al. Ensembl regulation resources. Database (Oxford). 2016;2016:bav119.

38. Mount DW. Using the Basic Local Alignment Search Tool (BLAST). CSH Protocol. 2007;2007:pdb.top17.

39. Birney E, Durbin R. Using GeneWise in the Drosophila annotation experiment. Genome research. 2000;10:547–548.

40. Kim D, Langmead B, Salzberg SL. HISAT: a fast spliced aligner with low memory requirements. Nature Methods. 2015;12:357–360.

41. Ghosh S, Chan C. Analysis of RNA-Seq Data Using TopHat and Cufflinks. Methods in Molecular Biology. 2016;1374:339–361.

42. Holt C, Yandell M. MAKER2: an annotation pipeline and genome-database management tool for second-generation genome projects. BMC Bioinformatics. 2011;12:491.

43. O'Leary NA, Wright MW, Brister JR, Ciufo S, Haddad D, McVeigh R, et al. Reference sequence (RefSeq) database at NCBI: current status, taxonomic expansion, and functional annotation. Nucleic Acids Research. 2016;44:D733–745.

44. Boutet E, Lieberherr D, Tognolli M, Schneider M, Bansal P, Bridge AJ, et al. UniProtKB/Swiss-Prot, the Manually Annotated Section of the UniProt KnowledgeBase: How to Use the Entry View. Methods in Molecular Biology. 2016;1374:23–54.

45. Gene Ontology Consortium. The Gene Ontology (GO) database and informatics resource. Nucleic Acids Research. 2004;32:D258–261.

46. Kanehisa M, Furumichi M, Tanabe M, Sato Y, Morishima K. KEGG: new perspectives on genomes, pathways, diseases and drugs. Nucleic Acids Research. 2017;45:D353–361.

47. Finn RD, Attwood TK, Babbitt PC, Bateman A, Bork P, Bridge AJ, et al. InterPro in 2017-beyond protein family and domain annotations. Nucleic Acids Research. 2017;45:D190–199.

48. Chen F, Mackey AJ, Stoeckert CJ, Roos DS. OrthoMCL-DB: querying a comprehensive multi-species collection of ortholog groups. Nucleic Acids Research. 2006;34:D363–368.

49. Edgar RC. MUSCLE: multiple sequence alignment with high accuracy and high throughput. Nucleic Acids Research. 2004;32:1792–1797.

50. Stamatakis A. RAxML version 8: a tool for phylogenetic analysis and post-analysis of large phylogenies. Bioinformatics. 2014;30:1312–1313.

51. Yang Z. PAML 4: phylogenetic analysis by maximum likelihood. Molecular Biology and Evolution. 2007;24:1586–1591.

52. Kumar S, Stecher G, Suleski M, Hedges SB. TimeTree: A Resource for Timelines, Timetrees, and Divergence Times. Molecular Biology and Evolution. 2017;34:1812–1819.

53. Martone PT, Estevez JM, Lu F, Ruel K, Denny MW, Somerville C, et al. Discovery of lignin in seaweed reveals convergent evolution of cell-wall architecture. Current Biology. 2009;19:169–175.

54. Kersey PJ, Allen JE, Allot A, Barba M, Boddu S, Bolt BJ, et al. Ensembl Genomes 2018: an integrated omics infrastructure for non-vertebrate species. Nucleic Acids Research. 2018;46:D802–808.

55. Zhao H, Peng Z, Fei B, Li L, Hu T, Gao Z, et al. BambooGDB: a bamboo genome database with functional annotation and an analysis platform. Database (Oxford). 2014;2014:bau006–6.

56. Taylor JS, Raes J. Duplication and divergence: the evolution of new genes and old ideas. Annual Review Genetics. 2004;38:615–643.

57. Zhao H; Wang S; Wang J; Chen C; Hao S; Chen L, et al. Chromosome-level genome assembly of *Calamus simplicifolius*. GigaScience Database. 2018. http://dx.doi.org/10.5524/101052

58. Zhao H; Wang S; Wang J; Chen C; Hao S; Chen L, et al. Chromosome-level genome assembly of *Daemonorops jenkinsiana*. GigaScience Database. 2018. http://dx.doi.org/10.5524/101053

59. Zhao H; Wang S; Wang J; Chen C; Hao S; Chen L, et al. Supporting data for "The chromosome-level genome assemblies of two rattans (*Calamus simplicifolius* and *Daemonorops jenkinsiana*)". GigaScience Database. 2018. http://dx.doi.org/10.5524/100480

Figure legends

Figure 1. Morphological characteristics of *C. simplicifolius* and *D. jenkinsiana*

The pictures in series A and B display the different morphological characteristics of *C. simplicifolius* and *D. jenkinsiana*, respectively. (a1) a young *C. simplicifolius*; (a2) a developing *C. simplicifolius*; (a3) a climbing *C. simplicifolius*; (a4) a mature *C. simplicifolius*; (a5) a nursery of *C. simplicifolius*; (b1) a young *D. jenkinsiana*; (b2) a young forest of *D. jenkinsiana*; (b3) a nursery of *D. jenkinsiana*; (b4) leaves of *D. jenkinsiana*; (b5) inflorescences of *D. jenkinsiana*; (b6) young fruits of *D. jenkinsiana*. All the pictures were taken by Prof. Rongsheng Li.

Figure 2. Hi-C contact map of the *C. simplicifolius* (a) and *D. jenkinsiana* genomes (b)

(c) and (d) show the Hi-C links on hic_scaffold_4 of *C. simplicifolius* and hic_scaffold_10 of *D. jenkinsiana* before (top) and after (bottom) conflict resolution. (e) and (f) show the distribution of Hi-C link decay along the genomic distance.

Figure 3. Distribution of the sequence divergence rates of different TE types in the *C. simplicifolius* (a) and *D. jenkinsiana* (b) genomes

Figure 4. The phylogenetic tree, orthologous gene families and divergence times among *C. simplicifolius*, *D. jenkinsiana*, and 8 other plants

(a) The phylogenetic tree was constructed by RAxML using all single-copy genes in the 10 species, and the divergence times were estimated using the MCMCTree program in the PAML software package. (b) Clusters of orthologous and paralogous gene families in *C. simplicifolius*, *D. jenkinsiana* and other 8 fully sequenced plants using OrthoMCL. (c) The numbers on the nodes are divergence times, and the red nodes indicate the calibration times.

Table 1: Statistics of the clean data of the *C. simplicifolius* and *D. jenkinsiana* genomes

Sequencing Platform	Insert Size	*C. simplicifolius*			*D. jenkinsiana*		
		**Read Length (bp)**	**Total Data (Gb)**	**Sequence Depth (X)***	**Read Length (bp)**	**Total Data (Gb)**	**Sequence Depth (X)***
Illumina	270 bp	150	160.9	82.09	150	98.21	61.38
	500 bp	125	60.2	30.71	125	56.9	35.56
	800 bp	125	101.2	51.63	125	89.47	55.91
	2 Kb	49	22.8	11.63	49	33.08	20.67
	5 Kb	49	16.4	8.37	49	22.1	13.81
	10 Kb	49	26.8	13.67	49	32.63	20.39
	20 Kb	49	27.4	13.98	49	15.4	9.6
PacBio	20 Kb	9,079**	78.38	39.99	9,131**	78.38	48.75
Hi-C	N.A.	100	6.7	3.42	100	13.1	8.19
Total			500.78	255.5		439.27	274.26

*Read length for PacBio means the average length

**Sequencing depth was calculated based on a 1.98 Gb *C. simplicifolius* genome and 1.61 Gb *D. jenkinsiana* genome

Table 2: Metrics of the final assemblies of the *C. simplicifolius* and *D. jenkinsiana* genomes

Items		*C. simplicifolius*		*D. jenkinsiana*	
		**Hybrid assembly***	**Hi-C assembly**	**Hybrid assembly***	**Hi-C assembly**
**Contig**	Number	29,973	29,973	27,631	27,631
	Size (bp)	1,923,260,127	1,923,260,127	1,570,849,893	1,570,849,893
	N50 (bp)	99,304	99,304	89,562	89,562
	N90 (bp)	28,872	28,872	25,720	25,720
**Scaffold**	Number	29,775	5,283	27,146	5,126
	Size (bp)	1,923,287,712	1,935,533,712	1,570,878,714	1,581,888,714
	N50 (bp)	99,590	160,072,219	89,705	119,093,744
	N90 (bp)	28,922	93,668,489	25,828	61,330,142
**Total number**	>3 kb	29,767	5,275	27,137	5,117
	>5 kb	29,727	5,235	27,081	5,061
**Longest sequence (bp)**		877,470	219,145,773	1,422,351	162,635,149
**Shortest sequence (bp)**		1,286	1,286	719	719
**Ns ratio (%)**		0.0	0.6	0.0	0.7
**GC ratio (%)**		41.07	41.07	41.78	41.78

* Hybrid assembly means *de novo* assembly using Illumina and PacBio data in our study

Table 3. Numbers of genes in gene families of the lignin biosynthesis pathway

Family	*C. simplicifolius*	*D. jenkinsiana*	*A. thaliana*	*B. distachyon*	*O. sativa*	*Ph. edulis*	*Po. trichocarpa*	*S. bicolor*	Total
4CL: 4-coumarate CoA ligase	9	13	12	13	12	15	13	16	90
C3H: Coumarate 3-hydroxylase	3	2	3	1	1	3	3	2	15
C4H: Cinnamate 4-hydroxylase	3	2	1	2	3	6	2	2	19
CAD: Cinnamyl alcohol dehydrogenase	29	22	9	7	10	14	17	11	102
CCoAOMT: Caffeoyl-CoA 3-O-methyltransferase	16	5	4	7	6	9	5	5	52
CCR: Cinnamoyl-CoA reductase	6	6	3	9	12	17	10	11	64
COMT: Caffeic acid 3-O-methyltransferase	13	16	11	4	6	4	11	5	59
F5H: Ferulate 5-hydroxylase	7	6	1	4	5	16	17	11	50
HCT: Hydroxycinnamoyl-CoA	5	4	3	12	6	16	7	13	59
LAC: Laccase	29	29	16	22	20	41	47	21	178
PAL: Phenylalanine ammonia-lyase	2	7	4	9	8	12	5	10	52
CHS: Chalcone synthase	31	17	4	7	17	12	13	27	115
POD: Peroxidase	40	43	45	44	37	77	56	42	328
Total	193	172	116	141	143	242	206	176	--

Figure 1

Click here to access/download;Figure;Fig1.Morphlogical features.pdf

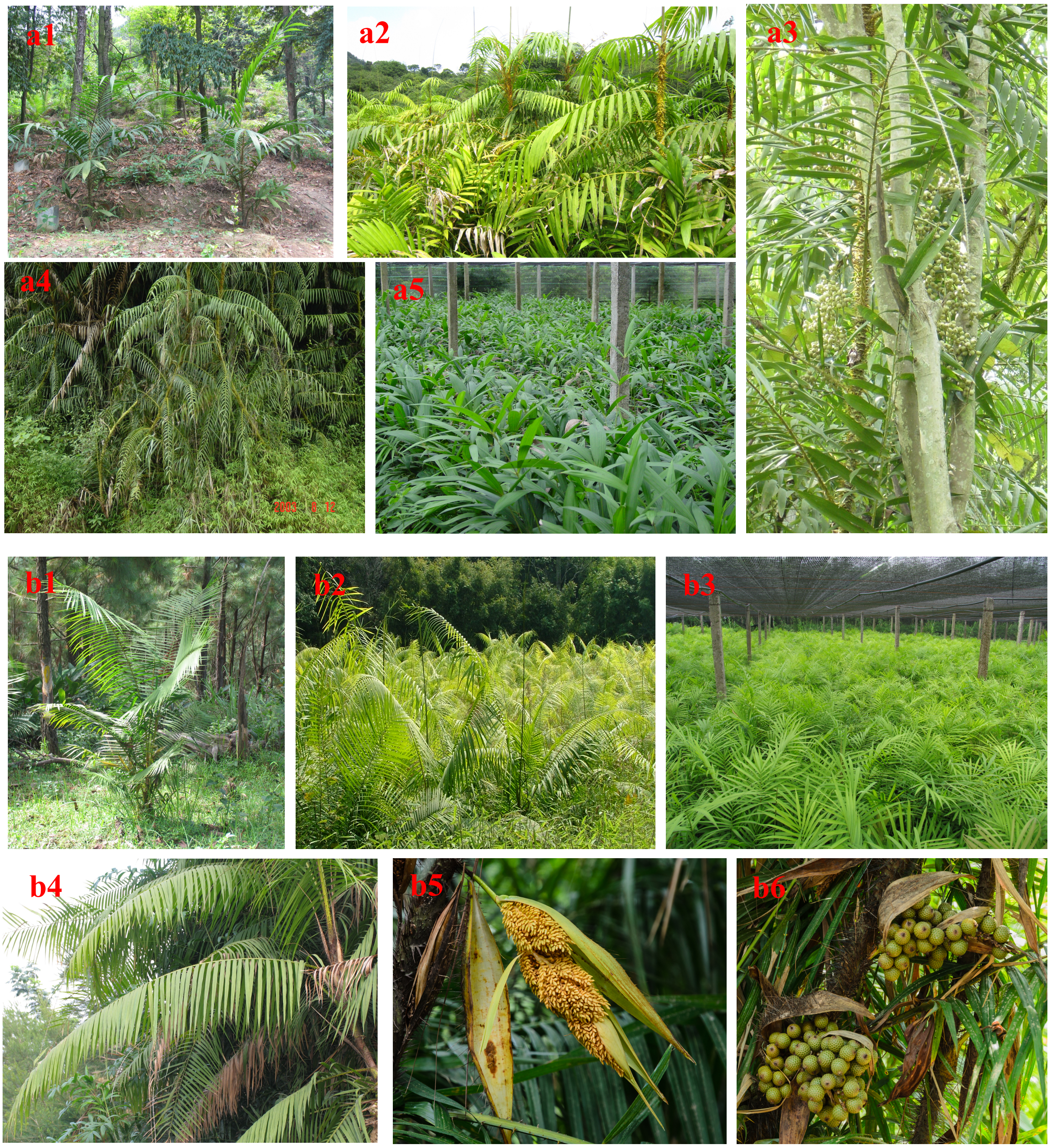

Figure 2

Click here to access/download;Figure;Fig2.Hic.pdf

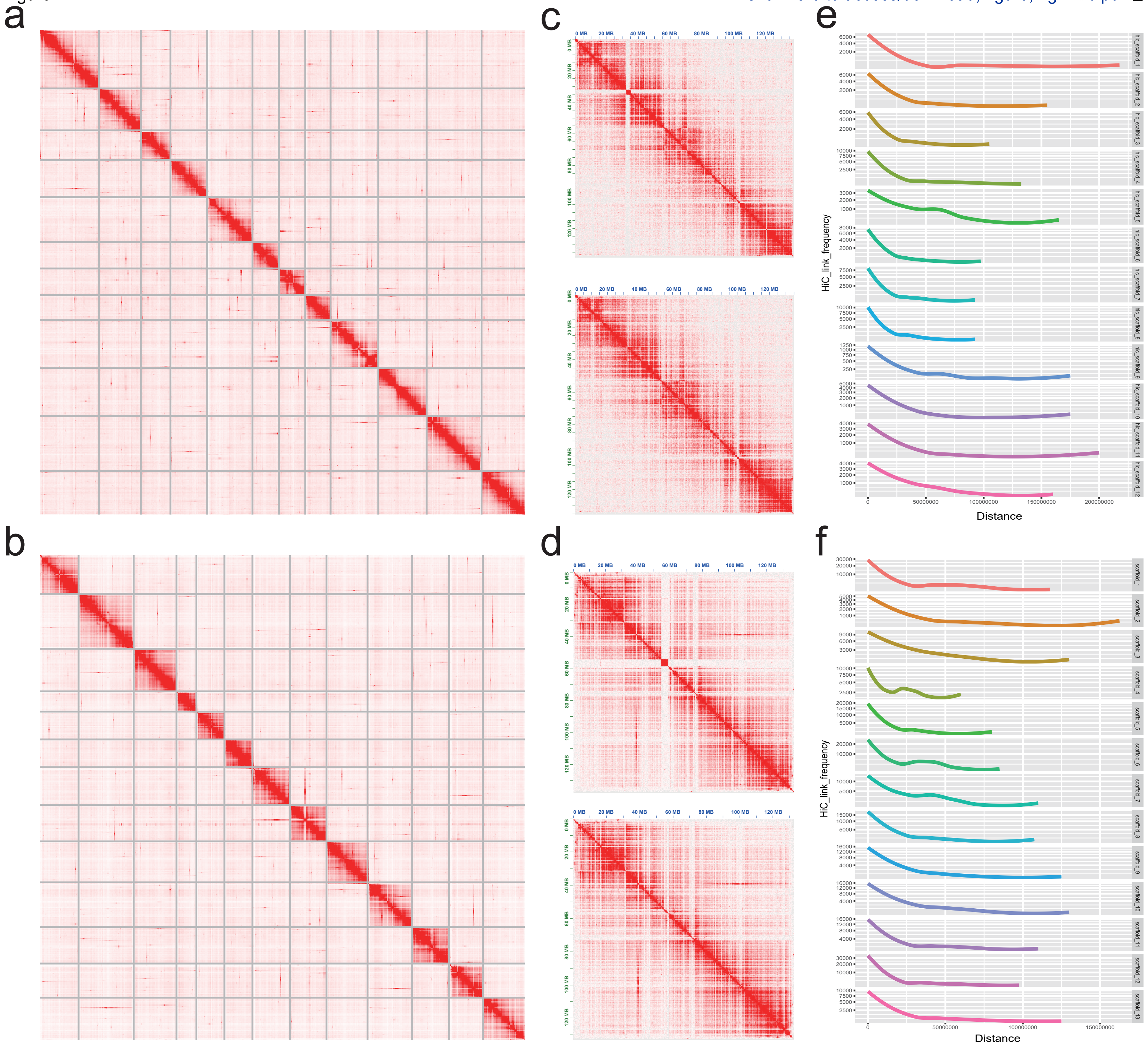

Figure 3

Click here to access/download;Figure;Fig3.Divergence rate distribution of different types of TE.pdf

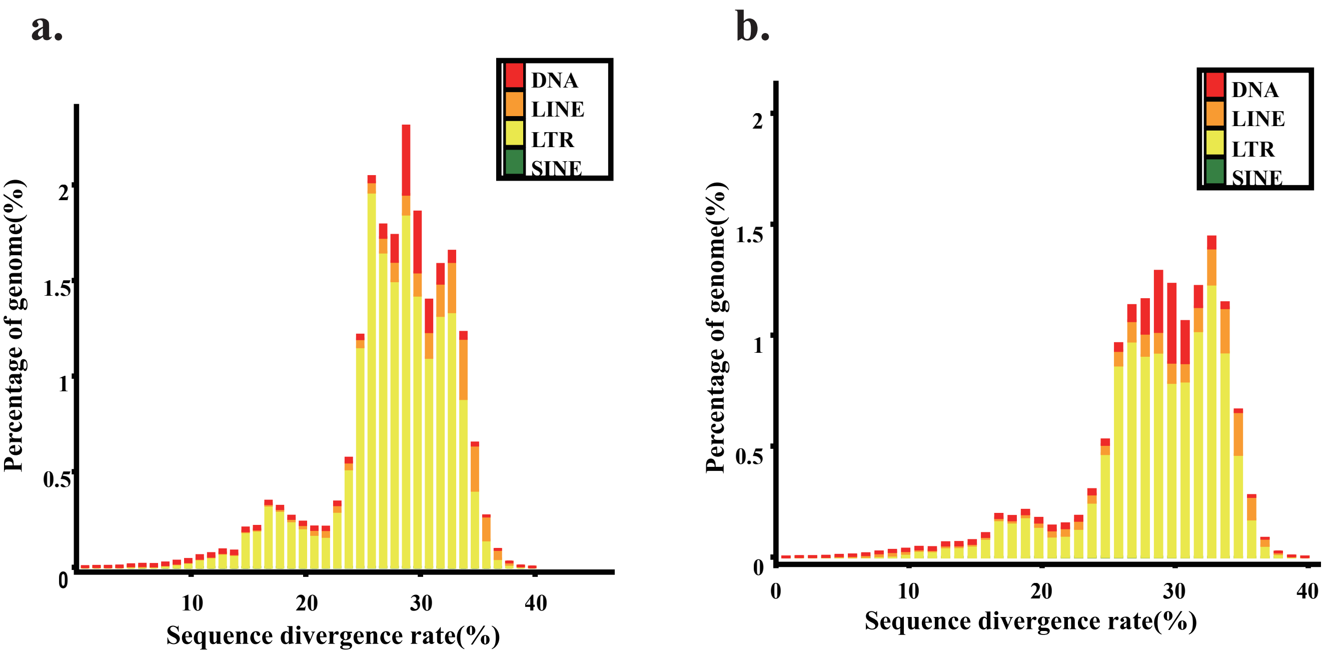

Figure 4

Click here to access/download;Figure;Fig4.phylogenetic and divergence time add P.dactylifera(1).pdf

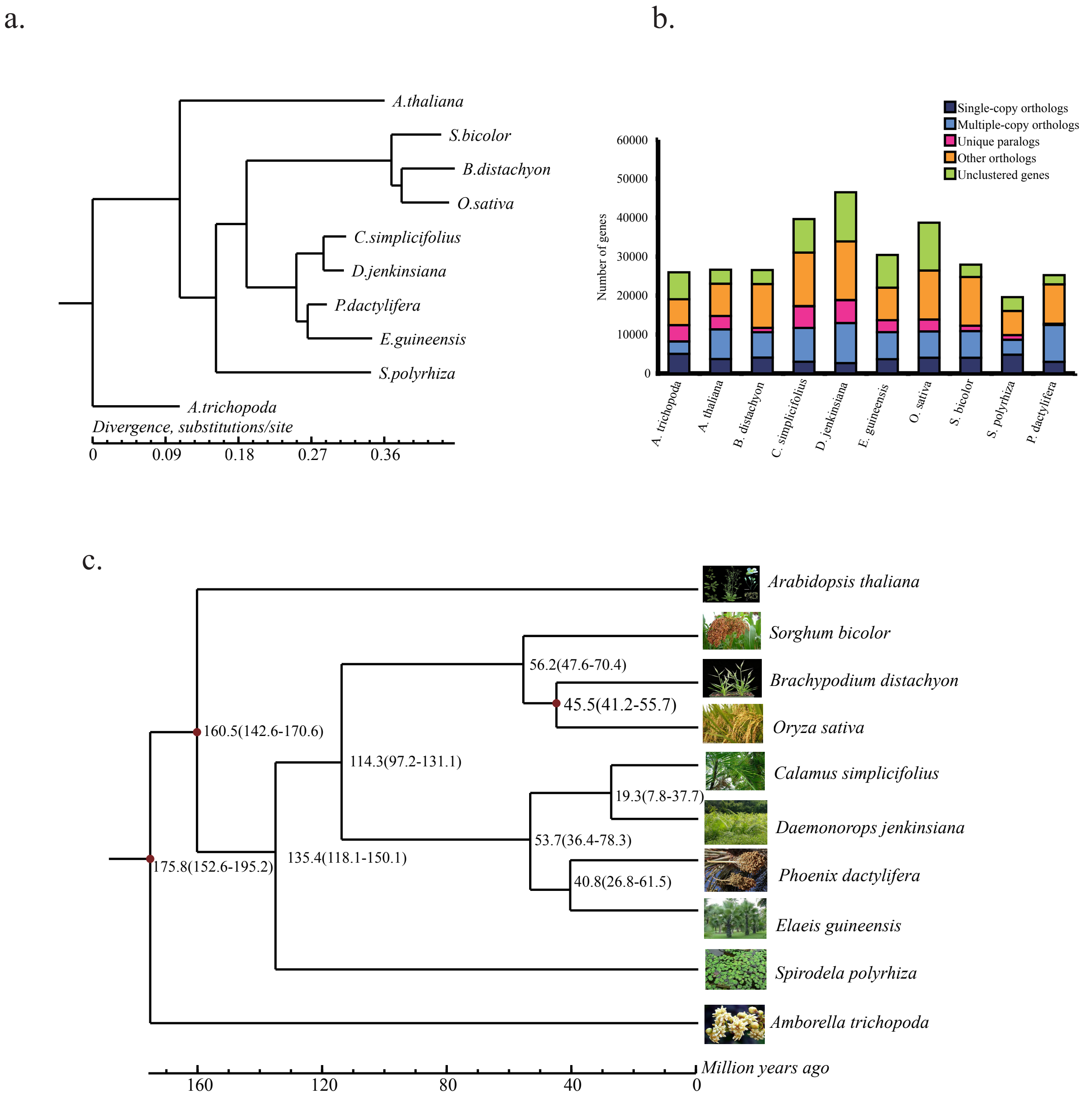

Additional Files

Click here to access/download
Supplementary Material
Additional Files-R2-725.xlsx

Additional Figures

Click here to access/download
Supplementary Material
Additional Figures-725.docx
