# Supplementary material for: The chromosome-level genome assemblies of two rattans (Calamus simplicifolius and Daemonorops jenkinsiana)
Source: Gigascience. 2018 Aug 7;7(9):giy097. doi: 10.1093/gigascience/giy097 (PMC6117794; doi:10.1093/gigascience/giy097)
Supplement: Supplemental Files [file giy097_supplemental_files.zip › Additional Figures-725.docx]

**Figure S1. Evaluation of the genome size of *C. simplicifolius* and** ***D. jenkinsiana* by 17-mer analyses**

The distribution of 17-mer depth of high-quality reads. Approximately 98 Gb and 60 Gb of sequencing reads from short-insert size libraries in *C. simplicifolius* (red line) and *D. jenkinsiana* (blue line), respectively, were split into 17 bp in length (17-mers) to plot the frequency (depth) of those 17-mers. The X-axis represents the sequencing depth and the Y-axis represents the frequency of those 17-mers at a given sequencing depth. Genome size was estimated according to the distribution (Table S2). The frequency exhibits a bi-modality due to the heterozygosity.


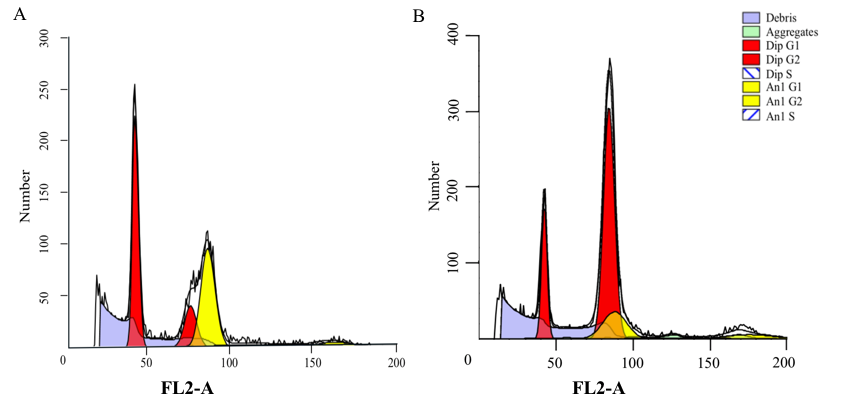


**Figure S2. Evaluation of genome size of *C. simplicifolius* and** ***D. jenkinsiana* by flow cytometry.**

A) Red represents tomato (*Solanum lycopersicum*), whose position is 42.01. Yellow represents *C. simplicifolius*, whose position is 96.92. the genome size of *C. simplicifolius* can estimated by the formula (Gc= peakc /peaks * Gs = 96.92/42.01 * 0.9 = 2.05 Gb).

B) Red represents tomato (*Solanum lycopersicum*), whose position is 43.84. Yellow represents *C. simplicifolius*, whose position is 83.112. the genome size of *D. jenkinsiana* can estimated by the formula (Gc= peakc /peaks * Gs = 83.11/43.84 * 0.9 = 1.71 Gb).
