# Supplementary material for: The chromosome-level genome assemblies of two rattans (Calamus simplicifolius and Daemonorops jenkinsiana)
Source: Gigascience. 2018 Aug 7;7(9):giy097. doi: 10.1093/gigascience/giy097 (PMC6117794; doi:10.1093/gigascience/giy097)
Supplement: Response_to_Reviewer_Comments_Original_Submission.pdf [file giy097_response_to_reviewer_comments_original_submission.pdf]

Author's Response To Reviewer Comments

Close

Scott Edmunds
Executive Editor
GigaScience

3 Jun 2018

Dear Dr. Scott,

Re: Manuscript reference No. GIGA-D-18-00152R1
Please find attached a revised version of our manuscript “The chromosome-level genome assemblies of two rattans (Calamus simplicifolius and Daemonorops jenkinsiana)”, which we would like to resubmit for publication as a research article in GigaScience.

The comments of two Reviewer were highly insightful and enabled us to greatly improve the quality of our manuscript. In the following pages are our point-by-point responses to each of the comments and suggestions of the Reviewer.

Revisions in the text are shown using red highlight. In accordance with the two reviewers’ suggestions, we carefully revised our manuscript, e.g., re-assembling the two genomes and adding the analyses of GO and lignin biosynthesis pathway. We hope that the new submission and our accompanying responses will be sufficient to make our manuscript suitable for publication in GigaScience.

We shall look forward to hearing from you at your earliest convenience.

Yours sincerely,

Prof. Hansheng Zhao
Address: No. 8, Fu Tong Dong Da Jie, Chaoyang District, Beijing 100102, P.R. China
Tel: +86-010-8478 9804
Fax: +86-010-8478 9802
E-mail: zhaohansheng@icbr.ac.cn

Responses to the comments of Reviewer #1
This manuscript provides two genomes for plant research, which is valuable. However, the data analysis still needs to be improved before this resource is released.

1. The analysis of genome sizes was not done comprehensively. The genome sizes were critical for the result, including the evaluation of assembly completeness. The module "FindErrors" in AllPATHS-LG was designed to correct errors in sequencing reads by counting kmers. I would suggest the authors to adopt multiple tools with statistics models to estimate genome size and compare the results. Besides this, it is also not clear how the genome size was calculated from the figure 2.

Response: Thank you for this excellent suggestion. We are sorry for the confused description of “FindErorrs”. Indeed, a script of ErrorCorrrectReads.pl in ALLPATHS-LG was used to correct reads by counting K-mer and then three output files of K-mer frequents (.kspec) were generated. Subsequently, the .kspec files could be analyzed using a script of KmerSpectrumPlot.pl in ALLPATHS-LG based on K-mer statistics to evaluate some genome features including the genome size. Additionally, we had also performed another three methods to access the genome sizes, including GCE (Genome Characteristics Estimation, released 20150107, ftp://ftp.genomics.org.cn/pub/gce), JELLYFISH (version 2.0) [1] and flow cytometry (only for Calamus simplicifolius) (see Tables S1-2 and Figs. S1-2). According to your suggestion, we have re-evaluated the genome size on the basis of the combination from above methods of estimating the genome size. Furthermore, we have revised the related descriptions and provided Table S2 to explain the K-mer calculation in Figure 2, as follows:

“The genome size was estimated using four independence methods, i.e., a script of KmerSpectrumPlot.pl in ALLPATHS-LG (version r52488), GCE (Genome Characteristics Estimation, released 20150107, ftp://ftp.genomics.org.cn/pub/gce), JELLYFISH (version 2.0) [1] and flow cytometry (only for Calamus simplicifolius) (Additional Tables S1-2 and Fig. S1).”

2. It's a little surprising to see PacBio reads only delivered assembly with N50 ~60kb. Even though these two genomes have relatively high repeat content, assemblies of PacBio reads still worth another try. The author first used MECAT to correct reads, which may lose some coverage. Both MECAT and FALCON are assembly pipeline, it would make more sense to run them separately. Canu should also be tested.

Response: Thank you for this excellent suggestion. The evaluation showed high heterozygosity between the two rattans (1.32%~1.52% for C. simplicifolius and 1.19~1.31% for D. jenkinsiana). According to our experience of genome assembly, we found that PacBio data has the advantage of assembling high repetitive genome, but they are not very effective in assembling highly heterozygous ones. In our study, we had used ~20 X of clean data of PacBio after the read correction for ~40 X of raw data. Also, we had tried to assembly genome only using PacBio data but obtained bad performances (Table S3), which may be due to highly heterozygous genomes and/or less valid data of PacBio. On the other hand, the two software, MECAT and Canu, have similar pipelines and they were not good at highly heterozygous genomes, according to their software’s manual. We had assessed MECAT using the rattan data but also obtained a bad performance (Table S3). Taken together, we adopted a hybrid assembly strategy including Illumina and PacBio data. Additionally, the different assembly strategies we tested were provided in Table S3.

3. PacBio reads were used for Gap-filling. This will request a polish process to correct the sequencing error. Without this step, the current gene annotation is not accurate, which was largely affected by errors in the sequence.

Response: Thank you for this excellent suggestion. We had provided a polish process before

the SSPACE process. The polish process referred to consensus analysis of DBG2OLC and obtained relatively good performances (Table S4), which were contributed to enhance the quality of the genome assembly and reduce errors in the SSPACE process. Additionally, no gap was detected in WGS assembly, which has less impact on subsequent gene annotations. Therefore, a polish process after gap-filling was unavailable in our study and we have revised the related context, as follows:

"Third, a polish process before the SSPACE process referred to consensus analysis of DBG2OLC (Table S4), which were contributed to enhance the quality of the genome assembly and reduce errors in the SSPACE process."

4. The author only used Fig. 3 to show the Hi-C data agreed with the sequence assembly very well. This is definitely not enough. The whole genome plot has limited resolution to find the conflict between the Hi-c links and the sequence. The author should have a detailed comparison of them to evaluate the assembly quality. Then do the scaffolding after resolve conflicts.

Response: Thank you for this excellent suggestion. Resolving the conflicts is very important for chromosome-level scaffolding. In our study, we used 3D-DNA pipeline [2], which has contained considered assessing steps for bad links to produce credible Hi-C assembly. We performed the pipeline and generated the whole genome Hi-C heat map which is commonly used to show an overview of Hi-C contacts on the longest chromosome-level scaffolds. According to your suggestion, we complementally checked the Hi-C contacts and calculated average link number along genomic distance for each longest chromosome-level scaffold to evaluate the assembly quality, and made revision by scaffolding after removing obvious conflict sequences, which have strong Hi-C links of inter-chromosome but take less relationships of intra-chromosome (hic_scaffold_1 and hic_scaffold_4 in C. simplicifolius genome and hic_scaffold_10, hic_scaffold_11, hic_scaffold_12 and hic_scaffold_13 in D. jenkinsiana genome). We have updated Figure 2 and presented one modified scaffold from the two genomes respectively as an example, as well as added the distribution of Hi-C contacts along the genomic distance, which is normally decayed.

Responses to the comments of Reviewer #2

In general, the authors have successfully performed a good if rather basic analysis for a genome paper. However, as outlined below, there are several issues that need to be addressed before the paper can be suitable for publication.

1. Unfortunately, there is a very little about any potential routes for application of the data presented. The rattans discussed in the paper are not really conventional crops in the sense of being grown in plantations and with a history of being bred for specific agronomic traits. Therefore, it is not clear how the genomic information presented here can be applied in the real world. The authors can address this point by outlining a more extensive rationale for the work in the Intro and then by discussing possible specific genes/traits that could be of interest for improving rattan species as biological resources.

Response: Thank you for this excellent suggestion. According to your suggestion, we have added the related description, as follows:

“C. simplicifolius and D. jenkinsiana have various applications and enormous developed potentials, and they are highly interesting mainly because of their canes with highly pliable and remarkably durable. Molecular breeding technology facilitate to meet growing requirements of the quantity and quality. However, the lack of genetic architecture underlying rattan’s important traits severely hampered the comprehensive understanding of molecular biology in scientific research and actual production and in-depth performing comparative genome analyses between and among related species. Thus, we reported the two de novo genome assemblies of C. simplicifolius and D. jenkinsiana using the latest sequencing (Illumina and PacBio) and Hi-C mapping technology. With the available of the two chromosome-level reference genomes in rattan, many comparative genome analyses and other downstream applications will become feasible, such as the development of biomarkers, the analysis of functional genes, molecular design breeding and so on. Additionally, a high-quality genome assembly of two rattans facilitates genomic, transcriptomic, and metabolomics analyses of the material traits. As possible specific genes that could be of interest for improving material property, the lignin gene family members are identified in rattan. These studies lay the foundation for future research to understand and utilize the genes that determine rattan quality and its diversity within rattan germplasm.”

2. As the authors already have the genome and transcriptome data, it is important for the authors to enlarge and be more specific in highlighting the contribution of this paper in their conclusions - possibly along the lines of "elucidation of this genome should be make it easier to identify genes involved in metabolite pathways that have potential developmental importance"

Response: Thank you for this excellent suggestion. We have added the section: “Genome-wide identification of gene families involved in the lignin biosynthesis pathway”, as follows:

“Genome-wide identification of gene families involved in the lignin biosynthesis pathway
Lignin is a class of complex aromatic heteropolymers of monolignols that encrusts and interacts with the cellulose/hemicellulose matrix of the secondary cell wall. The aromatic lignin polymers are commonly composed of three monolignols, i.e., p-hydroxyphenyl (H), vanillin (G), and syringaldehyde (S)[3]. Thus, we performed genome-wide identification of 13 gene families involved in the lignin biosynthesis pathway of rattan using the 8 genomes, i.e., A. thaliana, B. distachyon, O. sativa, S. bicolor, Phyllostachys edulis, Populus trichocarpa, D. jenkinsiana, and C. simplicifolius. Most genome sequences (A. thaliana, B. distachyon, O. sativa, S. bicolor, and Populus trichocarpa) were downloaded from the ENSEMBL database [4]. The genome sequences of Ph. edulis was downloaded from the Bamboo Genome Database [5]. According to vast literature-based investigations, 140 genes from the lignin biosynthetic pathway was collected from experimentally validation of previous studies (Additional Table S14), and then, these known genes were used as the query sequences for further identification. A BLAST search and domain analysis as described previously [54] was used in the genome-wide identification. Briefly, we performed standard protein BLAST searches (version 2.2.26) against all genome sequences including two rattans using the coding sequence of the known genes with the following cut-

off values: E-value <1e-10; identity >40%; and coverage rate >95% query sequence. The filtered sequences were subsequently analysed by hmmsearch (version 3.1b2) using the Pfam-A.hmm database (released 2017/03/31). Consequently, unclear sequences with incomplete domains were discarded by manual correction. The result showed the expansion of most families was detected in two rattans (Additional Table S15). Each gene contained multiple copies with an average of ~15 and ~13 copies per family in C. simplicifolius and D. jenkinsiana, respectively. The total size of the gene families in the lignin biosynthesis pathway contained 193 and 172 gene copies in C. simplicifolius and D. jenkinsiana, respectively. Peroxidase (POD), as the most copy number, was detected both in the two rattans. As the least copy number, phenylalanine ammonia-lyase (PAL) was identified in C. simplicifolius and coumarate 3-hdroxylase (C3H) and cinnamate 4-hydroxylase (C4H) were detected in D. jenkinsiana. The expansion of the lignin biosynthesis genes in rattan could be due to the occurrence of whole genome duplication (WGD) event, since WGD could provide more gene copies, which facilitated evolving the genes with new functions [6].”

3. We also suggest that the authors do some basic gene ontology analysis to discover how and which genes involved in key pathways of specific interest.

Response: Thank you for this excellent suggestion. We have added the GO and KEGG analysis of rattan specific-gene families, as follows:

“Additionally, the result showed that 637 gene families were specific to the rattans. The rattan species-specific gene families were enriched in gene ontology (GO) categories related to component membrane, transcription factor activity (Table S13) and in KEGG pathways related to plant-pathogen interaction, plant hormone signal transduction (Table S14).”

4. In this paper, the authors attempt to identify gene families for two genomes. But the gene family construction should be described in more detail to explain how the they identified the 29,240 gene families. Also in Figure 5 the authors should define what they mean by "unique paralogs" and also define "other orthologs". In general they should provide data and more detailed discussion for Figure 5.

Response: Thank you for this excellent suggestion. We have revised the part by providing more details, as follow:

“In our study, we performed a pairwise sequence comparison to genome-widely predict orthologous genes. The method is faster and generally deals with larger amounts of data. As a popular BLAST-based approach, OrthoMCL (version 2.0.9) [41] was used to identified orthologous genes in C. simplicifolius, D. jenkinsiana with the following BLASTP parameters: an E-value cutoff of 1e-5 and a percent match cut-off of 80 (i.e., query and match had to overlap on more than 80% of the query and match sequence length). Markov Chain Clustering was also used with a default inflation parameter in an all-to-all BLASTP analysis of entries for other 8 plants, i.e., Amborella trichopoda, E. guineensis, A. thaliana, B. distachyon, O. sativa, S. polyrhiza, P. dactylifera and S. bicolor (see Availability of supporting data).”

“Figure 5. Clusters of the orthologous and paralogous gene families in C. simplicifolius, D. jenkinsiana and other 7 full-sequenced plants using OrthoMCL.
Single-copy orthologs and multiple-copy orthologs represent one copy and more one copies from the gene families in each of selected species, respectively. Unique paralogs represent unique gene related by duplication within a given genome. Other orthologs represent gene duplication events internal to the overall set, but basal more than two of the compared species.”

5. Finally, since there is a P. dactylifera genome available and is in the same family as rattan and oil palm, it will be good to be including these two palm species in the phylogenetic analysis and calculate their possible divergence times.

Response: Thank you for this excellent suggestion. We have added the P. dactylifera genome for reconstructing phylogenetic relationship and revised the subsequent analysis based on the new result (Fig.4a).

Reference:
1. Marçais G, Kingsford C. A fast, lock-free approach for efficient parallel counting of occurrences of k-mers. Bioinformatics. 2011;27:764–70.
2. Dudchenko O, Batra SS, Omer AD, Nyquist SK, Hoeger M, Durand NC, et al. De novo assembly of the Aedes aegypti genome using Hi-C yields chromosome-length scaffolds. Science. 2017;356:92–5.
3. Martone P, Estevez J, Lu F, Ruel K, Denny MW, Somerville C, et al. Discovery of lignin in seaweed reveals convergent evolution of cell-wall architecture. Curr. Biol. 2009;19:169–75.
4. Kersey P, Allen J, Allot A, Barba M, Boddu S, Bolt B, et al. Ensembl Genomes 2018: an integrated omics infrastructure for non-vertebrate species. Nucleic Acids Res. 2018;46:D802–808.
5. Zhao H, Peng Z, Fei B, Li L, Hu T, Gao Z, et al. BambooGDB: a bamboo genome database with functional annotation and an analysis platform. Database (Oxford). 2014;2014:bau006.
6. Taylor J, Raes J. Duplication and divergence: the evolution of new genes and old ideas. Annu. Rev. Genet. 2004;38:615–43.

Close
