# Supplementary material for: The chromosome-level genome assemblies of two rattans (Calamus simplicifolius and Daemonorops jenkinsiana)
Source: Gigascience. 2018 Aug 7;7(9):giy097. doi: 10.1093/gigascience/giy097 (PMC6117794; doi:10.1093/gigascience/giy097)
Supplement: Response_to_Reviewer_Comments_Revision1.pdf [file giy097_response_to_reviewer_comments_revision1.pdf]

Author's Response To Reviewer Comments

Close

Scott Edmunds
Executive Editor
GigaScience

25 July 2018

Dear Dr. Scott,

Re: Manuscript reference No. GIGA-D-18-00152R2
Please find attached a revised version of our manuscript "The chromosome-level genome assemblies of two rattans (Calamus simplicifolius and Daemonorops jenkinsiana)", which we would like to resubmit for publication as a research article in GigaScience.

The comments of two Reviewers were highly insightful and enabled us to improve the quality of our manuscript. In the following pages are our point-by-point responses to each of the comments and suggestions of the Reviewer.

The latest manuscript was revised by our team and AJE experts. We hope that the new submission and our accompanying responses will be sufficient to make our manuscript suitable for publication in GigaScience.

We shall look forward to hearing from you at your earliest convenience.

Yours sincerely,

Prof. Hansheng Zhao
Address: No. 8, Fu Tong Dong Da Jie, Chaoyang District, Beijing 100102, P.R. China
Tel: +86-010-8478 9804
Fax: +86-010-8478 9802
E-mail: zhaohansheng@icbr.ac.cn

Responses to the comments of Reviewer #1

The revised manuscript has addressed my comments properly. By merging two genomes together, the authors also did more survey into the biology of the Rattan, which also made the story more interesting. There are a few other points the authors could improve before publication:

1. A new section related to the lignin biosynthesis pathway was added in the revision. However, the concept of the copy number of a gene family is not clear. For example, the first gene family from Table 3 "4CL" has 12 paralogue genes from EnsemlPlants. They are normally interpreted as 12 gene members from the "4CL" family - not 12 copies of a particular gene "4CL". The current description is quite misleading. Please revise.

Response: Thank you for this excellent suggestion. According to your suggestion, we have revised the description, as follows:

“Each gene family contained multiple gene members with an average of ~15 and ~13 gene members per family in C. simplicifolius and D. jenkinsiana, respectively. The total size of the gene families in the lignin biosynthesis pathway contained 193 and 172 gene members in C. simplicifolius and D. jenkinsiana, respectively. Peroxidase (POD), as the most gene member, was detected both in the two rattans. As the least gene member, phenylalanine ammonia-lyase (PAL) was identified in C. simplicifolius and coumarate 3-hdroxylase (C3H) and cinnamate 4-hydroxylase (C4H) were detected in D. jenkinsiana.”

2. The name of the package for Hi-C data analysis is JuiceBox, not "Juicerbox". Besides, many citations in this section were wrongly labeled. Please check.

Response: Thank you for this excellent suggestion. According to your suggestion, we have revised the description, as follows:

“As shown in Fig. 2, the contact maps were visualized by JuiceBox (version 1.5.2).”

Responses to the comments of Reviewer #2

Reviewer #2: The authors have addressed all of our original comments in a detailed and satisfactory manner that includes the inclusion of substantially more text and new tables etc. This has resulted in a much improved paper that has increased relevance to plant breeders and users of rattan and related crops.

Response: Thank you for satisfying our revision and previous suggestions.

Close
