# Supplementary material for: The chromosome-level genome assemblies of two rattans (Calamus simplicifolius and Daemonorops jenkinsiana)
Source: Gigascience. 2018 Aug 7;7(9):giy097. doi: 10.1093/gigascience/giy097 (PMC6117794; doi:10.1093/gigascience/giy097)
Supplement: Reviewer_1_Report_(Original_Submission) -- Yinping Jiao — 5/17/2018 Reviewed [file giy097_reviewer_1_report_(original_submission).pdf]

Reviewer Report

Title: The chromosome-level genome assemblies of two rattans (Calamus simplicifolius and Daemonorops jenkinsiana)

Version: Original Submission **Date:** 5/17/2018

Reviewer name: Yinping Jiao

Reviewer Comments to Author:

This manuscript provides two genomes for plant research, which is valuable. However, the data analysis still needs to be improved before this resource is released.1. The analysis of genome sizes was not done comprehensively. The genome sizes were critical for the result, including the evaluation of assembly completeness. The module "FindErrors" in AllPATHS-LG was designed to correct errors in sequencing reads by counting kmers. I would suggest the authors to adopt multiple tools with statistics models to estimate genome size and compare the results. Besides this, it is also not clear how the genome size was calculated from the figure 2.2. It's a little surprising to see PacBio reads only delivered assembly with N50 ~60kb. Even though these two genomes have relatively high repeat content, assemblies of PacBio reads still worth another try. The author first used MECAT to correct reads, which may lose some coverage. Both MECAT and FALCON are assembly pipeline, it would make more sense to run them separately. Canu should also be tested.3. PacBio reads were used for Gap-filling. This will request a polish process to correct the sequencing error. Without this step, the current gene annotation is not accurate, which was largely affected by errors in the sequence.4. The author only used Fig. 3 to show the Hi-C data agreed with the sequence assembly very well. This is definitely not enough. The whole genome plot has limited resolution to find the conflict between the Hi-c links and the sequence. The author should have a detailed comparison of them to evaluate the assembly quality. Then do the scaffolding after resolve conflicts.

Level of Interest

Please indicate how interesting you found the manuscript: Choose an item.

Quality of Written English

Please indicate the quality of language in the manuscript: Choose an item.

Declaration of Competing Interests

Please complete a declaration of competing interests, considering the following questions:

- Have you in the past five years received reimbursements, fees, funding, or salary from an organisation that may in any way gain or lose financially from the publication of this manuscript, either now or in the future?
- Do you hold any stocks or shares in an organisation that may in any way gain or lose financially from the publication of this manuscript, either now or in the future?
- Do you hold or are you currently applying for any patents relating to the content of the manuscript?

- Have you received reimbursements, fees, funding, or salary from an organization that holds or has applied for patents relating to the content of the manuscript?
- Do you have any other financial competing interests?
- Do you have any non-financial competing interests in relation to this paper?

If you can answer no to all of the above, write 'I declare that I have no competing interests' below. If your reply is yes to any, please give details below.

I declare that I have no competing interests

I agree to the open peer review policy of the journal. I understand that my name will be included on my report to the authors and, if the manuscript is accepted for publication, my named report including any attachments I upload will be posted on the website along with the authors' responses. I agree for my report to be made available under an Open Access Creative Commons CC-BY license (http://creativecommons.org/licenses/by/4.0/). I understand that any comments which I do not wish to be included in my named report can be included as confidential comments to the editors, which will not be published.

I agree to the open peer review policy of the journal

To further support our reviewers, we have joined with Publons, where you can gain additional credit to further highlight your hard work (see: https://publons.com/journal/530/gigascience). On publication of this paper, your review will be automatically added to Publons, you can then choose whether or not to claim your Publons credit. I understand this statement. Yes
