# Supplementary material for: The chromosome-level genome assemblies of two rattans (Calamus simplicifolius and Daemonorops jenkinsiana)
Source: Gigascience. 2018 Aug 7;7(9):giy097. doi: 10.1093/gigascience/giy097 (PMC6117794; doi:10.1093/gigascience/giy097)
Supplement: Reviewer_1_Report_(Revision_1) -- Yinping Jiao — 06/22/2018 Reviewed [file giy097_reviewer_1_report_(revision_1).pdf]

Reviewer Report

Title: **The chromosome-level genome assemblies of two rattans (Calamus simplicifolius and Daemonorops jenkinsiana)**

Version: **Revision 1** **Date:** 6/22/2018

Reviewer name: **Yinping Jiao**

Reviewer Comments to Author:

The revised manuscript has addressed my comments properly. By merging two genomes together, the authors also did more survey into the biology of the Rattan, which also made the story more interesting. There are a few other points the authors could improve before publication:1) A new section related to the lignin biosynthesis pathway was added in the revision. However, the concept of the copy number of a gene family is not clear. For example, the first gene family from Table 3 "4CL" has 12 paralogue genes from EnsemlPlants. They are normally interpreted as 12 gene members from the "4CL" family - not 12 copies of a particular gene "4CL". The current description is quite misleading. Please revise.2) The name of the package for Hi-C data analysis is JuiceBox, not "Juicerbox". Besides, many citations in this section were wrongly labeled. Please check.

Level of Interest

Please indicate how interesting you found the manuscript: Choose an item.

Quality of Written English

Please indicate the quality of language in the manuscript: Choose an item.

Declaration of Competing Interests

Please complete a declaration of competing interests, considering the following questions:

- Have you in the past five years received reimbursements, fees, funding, or salary from an organisation that may in any way gain or lose financially from the publication of this manuscript, either now or in the future?
- Do you hold any stocks or shares in an organisation that may in any way gain or lose financially from the publication of this manuscript, either now or in the future?
- Do you hold or are you currently applying for any patents relating to the content of the manuscript?
- Have you received reimbursements, fees, funding, or salary from an organization that holds or has applied for patents relating to the content of the manuscript?
- Do you have any other financial competing interests?
- Do you have any non-financial competing interests in relation to this paper?

If you can answer no to all of the above, write 'I declare that I have no competing interests' below. If your reply is yes to any, please give details below.

I declare that I have no competing interests

I agree to the open peer review policy of the journal. I understand that my name will be included on my report to the authors and, if the manuscript is accepted for publication, my named report including any attachments I upload will be posted on the website along with the authors' responses. I agree for my report to be made available under an Open Access Creative Commons CC-BY license (http://creativecommons.org/licenses/by/4.0/). I understand that any comments which I do not wish to be included in my named report can be included as confidential comments to the editors, which will not be published.

I agree to the open peer review policy of the journal

To further support our reviewers, we have joined with Publons, where you can gain additional credit to further highlight your hard work (see: https://publons.com/journal/530/gigascience). On publication of this paper, your review will be automatically added to Publons, you can then choose whether or not to claim your Publons credit. I understand this statement. Yes
