# Supplementary material for: The chromosome-level genome assemblies of two rattans (Calamus simplicifolius and Daemonorops jenkinsiana)
Source: Gigascience. 2018 Aug 7;7(9):giy097. doi: 10.1093/gigascience/giy097 (PMC6117794; doi:10.1093/gigascience/giy097)
Supplement: Reviewer_2_Report_(Original_Submission) -- Denis Murphy — 05/29/2018 Reviewed [file giy097_reviewer_2_report_(original_submission).pdf]

Reviewer Report

Title: **The chromosome-level genome assemblies of two rattans (Calamus simplicifolius and Daemonorops jenkinsiana)**

Version: **Original Submission** **Date:** 5/29/2018

Reviewer name: **Denis Murphy**

Reviewer Comments to Author:

In general, the authors have successfully performed a good if rather basic analysis for a genome paper. However, as outlined below, there are several issues that need to be addressed before the paper can be suitable for publication. Unfortunately, there is a very little about any potential routes for application of the data presented. The rattans discussed in the paper are not really conventional crops in the sense of being grown in plantations and with a history of being bred for specific agronomic traits. Therefore it is not clear how the genomic information presented here can be applied in the real world. The authors can address this point by outlining a more extensive rationale for the work in the Intro and then by discussing possible specific genes/traits that could be of interest for improving rattan species as biological resources.As the authors already have the genome and transcriptome data, it is important for the authors to enlarge and be more specific in highlighting the contribution of this paper in their conclusions - possibly along the lines of "elucidation of this genome should be make it easier to identify genes involved in metabolite pathways that have potential developmental importance" We also suggest that the authors do some basic gene ontology analysis to discover how and which genes involved in key pathways of specific interest.In this paper, the authors attempt to identify gene families for two genomes. But the gene family construction should be described in more detail to explain how the they identified the 29,240 gene families. Also in Figure 5 the authors should define what they mean by "unique paralogs" and also define "other orthologs". In general they should provide data and more detailed discussion for Figure 5.Finally, since there is a P. dactylifera genome available and is in the same family as rattan and oil palm, it will be good to be including these two palm species in the phylogenetic analysis and calculate their possible divergence times.

Level of Interest

Please indicate how interesting you found the manuscript: Choose an item.

Quality of Written English

Please indicate the quality of language in the manuscript: Choose an item.

Declaration of Competing Interests

Please complete a declaration of competing interests, considering the following questions:

- Have you in the past five years received reimbursements, fees, funding, or salary from an organisation that may in any way gain or lose financially from the publication of this manuscript, either now or in the future?
- Do you hold any stocks or shares in an organisation that may in any way gain or lose financially from the publication of this manuscript, either now or in the future?
- Do you hold or are you currently applying for any patents relating to the content of the manuscript?
- Have you received reimbursements, fees, funding, or salary from an organization that holds or has applied for patents relating to the content of the manuscript?
- Do you have any other financial competing interests?
- Do you have any non-financial competing interests in relation to this paper?

If you can answer no to all of the above, write 'I declare that I have no competing interests' below. If your reply is yes to any, please give details below.

'I declare that I have no competing interests'

I agree to the open peer review policy of the journal. I understand that my name will be included on my report to the authors and, if the manuscript is accepted for publication, my named report including any attachments I upload will be posted on the website along with the authors' responses. I agree for my report to be made available under an Open Access Creative Commons CC-BY license (http://creativecommons.org/licenses/by/4.0/). I understand that any comments which I do not wish to be included in my named report can be included as confidential comments to the editors, which will not be published.

I agree to the open peer review policy of the journal

To further support our reviewers, we have joined with Publons, where you can gain additional credit to further highlight your hard work (see: https://publons.com/journal/530/gigascience). On publication of this paper, your review will be automatically added to Publons, you can then choose whether or not to claim your Publons credit. I understand this statement.Yes
